# Corpora Amylacea in the Human Brain Exhibit Neoepitopes of a Carbohydrate Nature

**DOI:** 10.3389/fimmu.2021.618193

**Published:** 2021-06-28

**Authors:** Marta Riba, Elisabet Augé, Iraida Tena, Jaume del Valle, Laura Molina-Porcel, Teresa Ximelis, Jordi Vilaplana, Carme Pelegrí

**Affiliations:** ^1^ Secció de Fisiologia, Departament de Bioquímica i Fisiologia, Universitat de Barcelona, Barcelona, Spain; ^2^ Institut de Neurociències, Universitat de Barcelona, Barcelona, Spain; ^3^ Centros de Biomedicina en Red de Enfermedades Neurodegenerativas (CIBERNED), Madrid, Spain; ^4^ Alzheimer’s Disease and Other Cognitive Disorders Unit, Neurology Service, Hospital Clínic, Institut d’Investigacions Biomèdiques August Pi i Sunyer (IDIBAPS), Universitat de Barcelona, Barcelona, Spain; ^5^ Neurological Tissue Bank, Biobanc-Hospital Clínic-IDIBAPS, Barcelona, Spain

**Keywords:** neoantigen, neoepitope, immunoglobulin M, natural antibodies, corpora amylacea, brain aging, hyperglycemia, diabetes

## Abstract

Corpora amylacea (CA) in the human brain are polyglucosan bodies that accumulate residual substances originated from aging and both neurodegenerative and infectious processes. These structures, which act as waste containers, are released from the brain to the cerebrospinal fluid, reach the cervical lymph nodes *via* the meningeal lymphatic system and may be phagocytosed by macrophages. Recent studies indicate that CA present certain neoepitopes (NEs) that can be recognized by natural antibodies of the IgM class, and although evidence of different kinds suggests that these NEs may be formed by carbohydrate structures, their precise nature is unknown. Here, we adapted standard techniques to examine this question. We observed that the preadsorption of IgMs with specific carbohydrates has inhibitory effects on the interaction between IgMs and CA, and found that the digestion of CA proteins had no effect on this interaction. These findings point to the carbohydrate nature of the NEs located in CA. Moreover, the present study indicates that, *in vitro*, the binding between certain natural IgMs and certain epitopes may be disrupted by certain monosaccharides. We wonder, therefore, whether these inhibitions may also occur *in vivo*. Further studies should now be carried out to assess the possible *in vivo* effect of glycemia on the reactivity of natural IgMs and, by extension, on natural immunity.

## Introduction

*Corpora amylacea* (CA) in the human brain are polyglucosan aggregates that were first described by J.E. Purkinje in 1837 (1). They are intracellular astrocytic bodies that accumulate mainly in the periventricular and subpial regions of the aged brain (2–4), but are also present in large numbers in specific areas of the brain in neurodegenerative conditions (5–8). Different studies postulate that the function of CA is to entrap residual products derived from aging or degenerative processes (2, 4, 9–12). Accordingly, while essentially constituted of polymerized hexoses (2, 3), with glucose the predominant one (3), a wide range of components have been attributed to CA. These products are mainly derived from neurons, astrocytes, oligodendrocytes or the blood (8, 12–17), or even related to viral, fungal or microbial infections (18–20). However, the presence of some of these components remain controversial (10, 21). In 2017, we reported that some positive immunostainings described in CA may have been induced by contaminant immunoglobulins M (IgMs) present in commercial antibodies, giving rise to false positive results (10). Later, in 2018, we reviewed and ruled out some of the components that have been reported to occur in CA. We observed that CA contain glycogen synthase (GS), which is presumed to take part in the formation of the polyglucosan structure, as well as ubiquitin and p62 proteins, which are both involved in the processing of waste products (21). Recently, an immunofluorescence study, which excluded the possible false positive staining caused by IgMs, reported that CA contain tau protein (22). The possible role of CA in trapping residual products is also supported by electron microscopy studies, which have described some cell debris inside the CA, including impaired mitochondria and lysosome-like vesicles, originating from degenerative processes (9, 12, 23). Moreover, we recently revealed that CA are released from the brain into the cerebrospinal fluid (CSF), reaching the cervical lymph nodes *via* the meningeal lymphatic system, and we observed that CA can be phagocytosed by macrophages *in vitro*. All these findings indicate that CA can act as containers that remove waste products from the brain (24).

CA share some features with a type of polyglucosan bodies, referred to as PAS granules, which appear in the mouse brain. Both, PAS granules and CA, are round or oval shaped and have a high polysaccharide content, and therefore become positively stained with periodic acid-Schiff (PAS). Both structures are age-related deposits that entrap residual products and present some neoantigens, or more specifically neoepitopes (NEs), which can be recognized by natural antibodies of the IgM isotype (9, 10, 21, 24–30). NEs are epitopes that originate *de novo* in physiological conditions and also in pathological situations such as ischemia, diabetes, atherosclerosis, cancer and inflammatory events, as well as in aging (31–35). For its part, natural antibodies are generated throughout a lifetime, even during fetal development and before external antigen exposure (36–39). Their repertoire and reactivity pattern have been determined throughout evolution and are remarkably stable within species and even between species (40, 41), explaining their presence as contaminants in a wide range of commercial antibodies. They constitute a first line of immune defense, but also have important functions in the physiology of the organism (42). Some of these natural antibodies are able to recognize the NEs that occur in, for example, cell remnants and senescent or apoptotic cells, taking part in their controlled elimination and, thus, contributing to the maintenance of tissue homeostasis (31, 37, 38, 41–45). Therefore, the presence of NEs in both CA and PAS granules and the existence of natural IgMs that target them reinforce the idea that CA and PAS granules are involved in brain cleaning or in protective processes.

The nature of the NEs present in CA and recognized by natural IgMs remains unknown but several observations suggest that they can be of a carbohydrate nature. For instance, the NEs contained in PAS granules of the mouse brain, recognized by natural IgMs, have a carbohydrate nature (27, 30). In addition, natural IgM antibodies have been described to recognize some carbohydrates, such as advanced glycation end products, whose production is increased with aging (37, 44–48). In fact, many α-glycan antibodies present in human sera belong to the IgM class and are natural antibodies (49). In any case, IgMs often recognize epitopes that act as markers of own residual structures (42) and, as mentioned previously, CA contain some of these residual structures. Therefore, it seemed appropriate to explore whether the NEs of CA do have a carbohydrate nature. However, it has to be taken into account that in living beings glycans show great complexity, greater than that of proteins, nucleic acids and lipids, and currently there are few or at least insufficient tools to study glycans with the same ease and precision as that associated with the study of proteins (50). Thus, in this first attempt we adapted standard techniques to determine the nature of NEs of CA.

Two different approaches were used. First, IgM preadsorptions with increasing concentrations of specific carbohydrates were performed to ascertain if the preadsorption produced a partial or total inhibition of CA staining with the IgMs. As a control, the same preadsorption studies were undertaken by staining the CA with concanavalin A (ConA). ConA staining of CA has been previously described in the literature (24, 51). ConA is a plant lectin that binds to hexoses in which the d-pyranose ring system presents hydroxyl groups at C-3 and C-4 in the equatorial plane, as well as a hydroxyl group at C-6. These types of hexoses include d-mannose (Man) and d-glucose (Glc), but not d-galactose (Gal), in which the hydroxyl group at C-4 is in the axial plane. ConA also binds to certain sugars with a five-membered furanoid ring form, such as ketohexose d-fructose, in which the hydroxyl groups at C-3, C4 and C-6 are oriented similarly to those in the mannopyranosyl ring, and aldopentose d-arabinose, in which the hydroxyl groups at C-2, C-3 and C-5 show a similar orientation (52–54). Thus, if carbohydrate preadsorption works well, CA staining with fluorochrome-labeled ConA will be blocked by Glc, Man, d-fructose (Fru) and N-acetyl-d-glucosamine (GlcNAc), but not by Gal or N-acetyl-d-galactosamine (GalNAc). Another control of the preadsorption with sugars was based on the staining of CA with an α-p62 antibody. As p62 is a protein, the preadsorption of the α-p62 antibody with the different sugars should not interfere with the staining of CA. This control was used to demonstrate that the preadsorption with the sugars did not block the antibodies directed against the proteins in the CA.

The second approach to study the interaction of the IgMs with CA involved staining the CA after digestion with the peptidic enzyme pepsin. If IgM recognizes glycosides that are not linked to proteins, the staining should be maintained after the digestion. As control experiments, AF555 NHS ester probe, α-p62 antibody and fluorescein-labeled ConA were used to stain CA. The AF555 NHS ester probe labels primary amines (R-NH2) of proteins, amine-modified oligonucleotides, and other amine-containing molecules. On the one hand, since AF555 NHS ester probe and α-p62 bind to proteins, pepsin digestion might prevent the staining of CA with them; on the other hand, the staining with ConA should not be affected since ConA binds to carbohydrate structures.

## Material and Methods

### Preadsorption Studies

#### Human Brain Samples

Post-mortem brain samples were obtained from the Banc de Teixits Neurològics (Biobanc-Hospital Clínic-IDIBAPS, Barcelona). Frozen hippocampal sections (6 µm-thick; stored at -80°C) were obtained from three cases of neuropathologically confirmed Alzheimer’s disease, two cases of subcortical vascular encephalopathy and one case of Lewy body dementia. Medical data of these cases are detailed in **Table S1 (Supplementary Material)**. All procedures involving human samples were performed in accordance with appropriate guidelines and regulations. All experiments involving human tissue were approved by the Bioethical Committee of the Universitat de Barcelona.

#### Immunofluorescence and ConA Staining

Frozen hippocampal sections were air dried for 10 min and then fixed with acetone for 10 min at 4°C. After 2 h of drying, sections were rehydrated in phosphate-buffered saline (PBS) and then blocked and permeabilized with 1% bovine serum albumin in PBS (Sigma-Aldrich) (blocking buffer, BB) with 0.1% Triton X-100 (Sigma-Aldrich) for 20 min. They were then washed with PBS and incubated with the primary antibody overnight at 4°C. The slides were then washed and incubated for 1 h at room temperature with the secondary antibody. Nuclear staining was performed with Hoechst (2 μg/mL, H-33258, Fluka, Madrid, Spain) and the slides were washed and coverslipped with Fluoromount (Electron Microscopy Sciences, Hatfeld, PA, USA). Staining controls were performed by incubating with BB instead of the primary antibody before incubation with the secondary antibody.

The following were used as primary antibodies: mouse monoclonal IgG_2a_ against p62 (diluted 1:200; Abcam, ab56416), human IgMs purified from myeloma serum (MS-IgMs) (1:10; AbD Serotec, OBT1524) and human IgMs purified from normal serum (NS-IgMs) (1:25; Sigma, I8260). Previous studies indicated that both pools of IgMs contain IgMs that bind to CA. The following were used as the secondary antibodies: Alexa Fluor (AF) 488 goat α-mouse IgG_2a_ (1:250; Life Technologies, A-21131) and AF488 goat α-human IgM (heavy chain) (1:200; Life Technologies, A-21215).

In some of the experiments, samples were incubated overnight with fluorescein-labeled ConA (1:250; Vector Laboratories) instead of the primary antibody and, as incubation with the secondary antibody was not needed, the slides were immediately washed and coverslipped with Fluoromount.

#### Preadsorption With Carbohydrates

Primary antibodies and ConA were preadsorbed in different assays with the monosaccharides Glc (Scharlab, GL01271000), Man (Sigma Aldrich, M2D69), Fru (Sigma Aldrich, F0127), Gal (Sigma Aldrich, G0750), GlcNAc (Sigma Aldrich, A8625) and GalNAc (Sigma Aldrich, A2795). For each monosaccharide, we first prepared sugar dilutions of 0 (control), 50, 100, 200 and 400 mM in PBS, as well as 800 mM in some of the indicated cases. The preadsorption was then performed by using these sugar solutions to dilute the primary antibodies or ConA at the indicated concentrations, and the mixtures were maintained overnight at 4°C with agitation. The different hippocampal sections were incubated with each mixture instead of the primary antibody. Some preadsorption series of different sugars were performed at the same time and the 0 mM tissue section was shared. Thus, these series show the same fluorescence values and images at 0 mM. To reduce variability, consecutive hippocampal sections were used in each assay, i.e., in the study of the preadsorption of an antibody (or ConA) with a determinate sugar.

#### Image Acquisition

Images were captured with a fluorescence laser and optical microscope (BX41, Olympus, Germany) and saved in tiff format. All the images for each combination of antibody (or ConA) and sugar were taken using the same microscope, laser and software settings. The time of capture was adapted to each staining, but the control or reference images were acquired with the same time of capture as that of the respective set of images.

#### Image Processing and Fluorescence Intensity Measurements

The merging of the images from the different fluorescence channels was performed using the FIJI program (National Institutes of Health, USA). Image analysis and quantifications were also performed with the FIJI program, not using the composite image, but the original image in the appropriate color. All CA of each hippocampal section were analyzed. The degree of staining of each CA was quantified as described below. For each CA, we first defined the region of interest (ROI) manually by tracing the outline of the CA using the elliptical selection tool of the FIJI program. We then obtained the mean gray value of the ROI, which corresponded to the mean fluorescence intensity (in arbitrary units) of the outlined CA. To minimize possible differences in the basal intensity of each image, for each CA, we moved the ROI to an area next to the CA and obtained the mean fluorescence intensity of the background. Thereafter, we obtained the relative intensity (RI) for each CA, which was defined as the difference between the mean fluorescence intensity of the CA and the mean fluorescence intensity of the respective background. To standardize the data obtained from the different preadsorption studies, for each combination of antibody and sugar, we defined the mean value of all the RIs obtained with 0 mM of the monosaccharide as the reference value (RV). The intensity of each CA was expressed as a percentage with respect to the reference value (RI%), i.e., 100*RI/RV. It should be noted that in all the cases, the mean RI% value will be 100% for 0 mM. If the carbohydrate preadsorption interferes with the CA staining, this percentage will decrease with increasing carbohydrate concentrations. The original data of the fluorescence intensities of the CA and their corresponding backgrounds are included in the **Supplementary Materials**.

#### Statistical Analysis

Statistical analysis was performed with the ANOVA module of SPSS Statistics (IBM). For each study, to determine if the sugar concentration influences the RI%, the variable sugar concentration has been defined as the independent variable and the RI% variable as the dependent one. When the ANOVA indicates that the variable sugar concentration influences the RI%, the Scheffe test was used for *post hoc* comparisons in order to compare the values obtained at each sugar concentration to the values obtained at 0 mM. Differences were considered significant when p < 0.01. However, as commented in the Editorial of Nature dated 20th March 2019 (55), using only p values to determine significance can also lead to some analyses being biased, some false positives being overhyped and some genuine effects being overlooked. Logic, background knowledge and experimental design should be considered alongside p values and similar metrics to reach a conclusion and decide on its certainty. Accordingly, in the present study, p values were used alongside other findings to reach conclusions based on a general view.

### Digestion Studies

#### Human CSF Samples Used to Obtain CA

Post-mortem ventricular CSF samples were obtained from three cases of neuropathologically affected patients and one non-neuropathologically affected patient. When extracted, the CSF samples were centrifuged at 4,000 *g* at 4°C for 10 min and the pellets obtained were stored at -80°C until use. All these procedures were performed at the Banc de Teixits Neurològics (Biobanc-Hospital Clínic-IDIBAPS, Barcelona). Medical data about these cases are detailed in **Table S2 (Supplementary Material)**.

#### CA Extraction and Pepsin Digestion

CSF pellets were re-suspended in PBS and filtered through a 35-µm porous filter (Fisher Scientific, 12934257). The samples were then centrifuged at 700 g for 10 min. The supernatants were then rejected and pellets were re-suspended in PBS to obtain 500-µL aliquots. Another centrifugation at 700 g for 10 min was performed, and the pellet was re-suspended in 500 μL of a solution of 4 mg/mL of pepsin (Merck, P6887) in 10 mM HCl (pH 2).The suspension was maintained under agitation overnight at 37°C. Control experiments were performed by incubating the samples overnight with PBS instead of the pepsin solution.

#### Immunofluorescence, AF555 NHS Ester Staining, and ConA Staining

After pepsin digestion, samples were washed 3 times by centrifugation at 700 g for 10 min and the pellet was re-suspended in 1,000 μL of PBS. The samples were then incubated with the primary antibody overnight at 4°C. Thereafter, samples were washed 3 times by centrifugation and incubated for 1 h at room temperature with the secondary antibody. Samples were then washed twice and centrifuged again at 700 g for 10 min to obtain pellets that were then re-suspended in 40 μL of PBS. These 40-μL samples were spread on to slides, air dried and coverslipped with Fluoromount. Staining controls were performed by incubating with PBS instead of the primary antibody.

The following were used as the primary antibodies: mouse monoclonal IgG_2a_ against p62 (diluted 1:200; Abcam, ab56416) and NS-IgMs (1:25; Sigma, I8260). The following were used as the secondary antibodies: AF555 goat α-mouse IgG_2a_ (1:250; Life Technologies, A-21131) and AF488 goat α-human IgM (heavy chain) (1:200; Life Technologies, A-21215).

In some of the immunofluorescence experiments, samples were incubated with fluorescein-labeled ConA (1:250; Vector Laboratories, FL-1001-25) overnight or with AF555 NHS ester probe (1 mg/mL; ThermoFisher Scientific, A37571) 1h at room temperature with agitation, instead of the primary antibodies.

In some cases, sequential double staining was performed with the α-p62 antibody and ConA and with the α-p62 antibody and NS-IgMs. The α-p62 antibody and ConA double staining was carried out in the same way as the simple immunofluorescence procedure, but adding an overnight incubation at 4°C with ConA after the incubation with the secondary antibody. Regarding the α-p62 antibody and NS-IgM double immunostaining, the samples were incubated with the α-p62 antibody overnight at 4°C first and then with NS-IgMs overnight at 4°C, before being incubated with the secondary antibodies simultaneously for 1 h at room temperature.

#### Image Acquisition and Processing

Image acquisition and processing were performed as described above for the sugar preadsorption studies.

## Results

### Sugar Preadsorption Studies

#### Effects of Sugar Preadsorption on ConA Staining of CA

**Figure 1:**
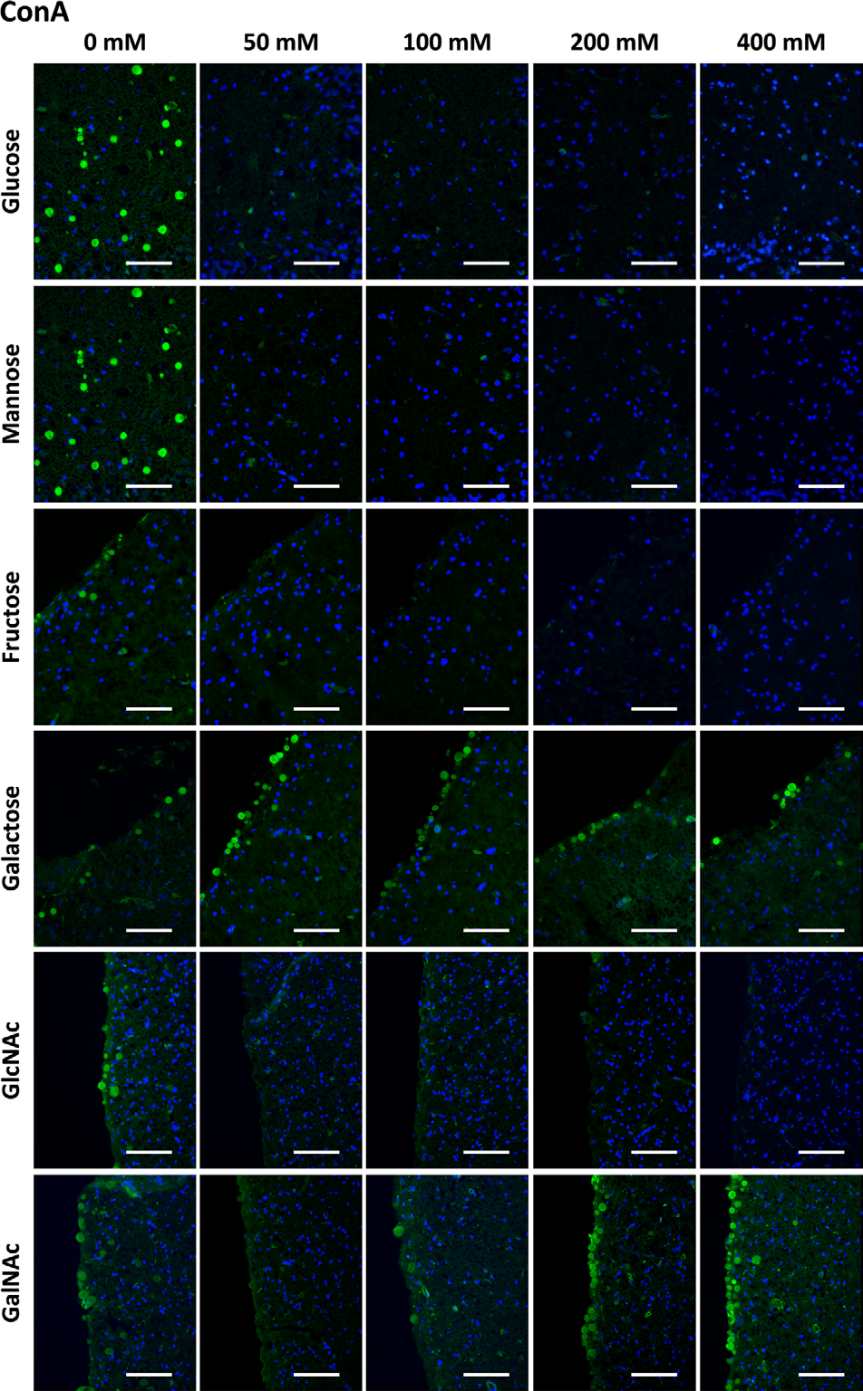
Representative images of hippocampal sections stained with ConA (green) and Hoechst (blue). Each row corresponds to a preadsorption study performed with the indicated carbohydrate. Sugar concentrations are indicated above. The staining of CA (green) disappeared or decreased when the preadsorption was performed with Glc, Man, Fru or GlcNAc, but was not affected by Gal or GalNAc. Some discrepancies in the basal staining can be appreciated between the different preadsorption experiments (see, for example, the first column corresponding to the positive controls); for the statistical analysis, these discrepancies have been corrected in basis of the background intensity, as indicated in the Methods section. Scale bar: 100 μm.

As mentioned before, it is well established that ConA binds to CA and that fluorochrome-labeled ConA can be used to stain CA (24, 51). Thus, we performed the preadsorption studies with the different sugars using fluorochrome-labeled ConA. As shown in the upper row of **Figure 1**, preadsorption with glucose interfered with the staining. While ConA staining of the CA can be clearly observed when the Glc concentration is 0 mM (i.e., positive control), the staining is practically absent in the presence of glucose at all the tested concentrations. The same pattern was observed when the preadsorption was performed with Man, Fru or GlcNAc. However, when the preadsorption was performed with Gal or GalNAc, the staining with ConA was maintained, even at the highest concentration of sugar.

**Table 1** presents the mean RI% values for each carbohydrate experiment and each carbohydrate concentration. This table contains the summary of all the results of the section to allow and facilitate the comparison between them. As can be observed, in the case of ConA staining, statistical analysis of the data from the Glc experiments indicated that the RI% decreased with the preadsorption, being significantly lower than 100% for all the tested concentrations (p < 0.01), i.e., lower than that of the reference value at 0 mM. This indicates that Glc significantly interfered with the ConA staining of CA. For Man, Fru and GlcNAc, there was also a significant decrease in the staining with increasing sugar concentrations. This was not the case for Gal and GalNAc, which did not reduce the staining. There were no significant differences with respect to their controls, except for GalNAc at 800 mM, which resulted in a mean RI% of 168.49 that was higher than the value at 0 mM. This was considered an outlier because of the absence of any clear trend at the other concentrations (see below). In any case, GalNAc did not decrease the staining when used to preadsorb ConA.

**Table 1:** Fluorescence intensity of CA staining expressed as mean RI% (± s.e.m.; N).

		0 mM	50 mM	100 mM	200 mM	400 mM	800 mM
**ConA**	Glc	100,00 ( ± 2,94; 20)	0,11 ( ± 0,11; 24)*	0,00 ( ± 0; 14)*	0,04 ( ± 0,04; 16)*	0,00 ( ± 0; 19)*	—
	Fru	100,00 ( ± 6,02; 7)	0,00 ( ± 0; 5)*	0,00 ( ± 0; 5)*	0,00 ( ± 0; 6)*	0,00 ( ± 0; 10)*	—
	Man	100,00 ( ± 2,94; 20)	0,01 ( ± 0,01; 29)*	0,00 ( ± 0; 17)*	0,00 ( ± 0; 13)*	0,00 ( ± 0; 20)*	—
	GlcNAc	100,00 ( ± 5,14; 8)	0,00 ( ± 0; 9)*	0,00 ( ± 0; 10)*	0,00 ( ± 0; 7)*	0,00 ( ± 0; 8)*	—
	Gal	100,00 ( ± 5,02; 20)	113,84 ( ± 7,5; 33)	114,29 ( ± 15,18; 12)	124,53 ( ± 12,1; 10)	85,61 ( ± 5,37; 18)	—
	GalNAc	100,00 ( ± 5,29; 19)	95,68 ( ± 7,8; 15)	135,36 ( ± 7,19; 33)	98,31 ( ± 10,56; 12)	168,49 ( ± 5,87; 57)**	—
**p62**	Glc	100,00 ( ± 5,43; 54)	112,71 ( ± 6,86; 57)	106,29 ( ± 6,04; 48)	110,03 ( ± 6,33; 49)	113,09 ( ± 8,77; 46)	110,43 ( ± 7,79; 47)
	Fru	100,00 ( ± 6,99; 64)	97,17 ( ± 7,76; 59)	83,91 ( ± 5,24; 56)	110,83 ( ± 7,33; 77)	90,7 ( ± 6,06; 54)	73,80 ( ± 5,10; 63)
	Man	100,00 ( ± 6,99; 64)	88,48 ( ± 6,62; 56)	108,27 ( ± 10,24; 36)	74,06 ( ± 5,47; 57)	92,53 ( ± 7,03; 68)	80,05 (6,76; 43)
	GlcNAc	100,00 ( ± 25; 12)	114,79 ( ± 34,35; 11)	54,88 ( ± 9,95; 16)	73,42 ( ± 14,22; 24)	126,59 ( ± 21,72; 14)	69,38 ( ± 10,03; 32)
	Gal	100,00 ( ± 8,06; 61)	65,22 ( ± 5,88; 32)	117,3 ( ± 7,51; 55)	123,98 ( ± 8,55; 55)	53,63 ( ± 11,47; 14)	80,99 ( ± 9,53; 34)
	GalNAc	100,00 ( ± 25; 12)	61,71 ( ± 5,46; 31)	81,42 ( ± 28,07; 11)	80,39 ( ± 17,28; 13)	57,94 ( ± 7,51; 24)	64,16 ( ± 9,74; 12)
**MS-IgM (S1)**	Glc	100,00 ( ± 7,66; 29)	80,28 ( ± 7,65; 20)	46,97 ( ± 3,64; 34)*	14,39 ( ± 1,68; 21)*	0,33 ( ± 0,23; 9)*	—
	Fru	100,00 ( ± 8,9; 19)	78,02 ( ± 15,23; 7)	69,59 ( ± 5,67; 6)	37,15 ( ± 5,17; 5)*	5,15 ( ± 1,21; 7)*	—
	Man	100,00 ( ± 7,66; 29)	69,57 ( ± 6,04; 30)*	27,45 ( ± 3,18; 14)*	25,32 ( ± 3,04; 13)*	0,65 ( ± 0,2; 7)*	—
	Gal	100,00 ( ± 8,9; 19)	66,21 ( ± 4,92; 8)	44,15 ( ± 2,9; 12)*	0,6 ( ± 0,47; 13)*	0,78 ( ± 0,36; 13)*	—
**MS-IgM (S2)**	Glc	100,00 ( ± 4,54; 26)	91,14 ( ± 4,81; 11)	41,77 ( ± 4,01; 9)*	35,61 ( ± 9,67; 2)*	4,3 ( ± 2,45; 10)*	—
	Fru	100,00 ( ± 3,14; 79)	48,69 ( ± 5,13; 27)*	37,08 ( ± 3,81; 34)*	28,51 ( ± 2,38; 31)*	0,41 ( ± 0,21; 28)*	—
	Man	100,00 ( ± 8,42; 39)	95,45 ( ± 6,98; 34)	90,21 ( ± 9,23; 15)	31,73 ( ± 8,21; 16)*	4,17 ( ± 1,36; 10)*	—
	Gal	100,00 ( ± 3,55; 66)	59,79 ( ± 3,24; 43)*	43,12 ( ± 2,15; 33)*	7,7 ( ± 0,7; 24)*	0,19 ( ± 0,12; 7)*	—
**NS-IgM (S1)**	Glc	100,00 ( ± 8,14; 53)	0,26 ( ± 0,26; 18)*	0,00 ( ± 0; 36)*	0,00 ( ± 0; 36)*	0,00 ( ± 0; 32)*	—
	Fru	100,00 ( ± 9,31; 9)	84,46 ( ± 7,61; 37)	65,51 ( ± 9,14; 11)	43,83 ( ± 2,39; 60)*	35,72 ( ± 4,95; 20)*	—
	Man	100,00 ( ± 9,31; 9)	63,4 ( ± 2,78; 21)*	57,41 ( ± 3,44; 43)*	44,69 ( ± 2,96; 17)*	42,07 ( ± 2,93; 22)*	—
	GlcNAc	100,00 ( ± 8,14; 53)	50,43 ( ± 6,85; 37)*	53,23 ( ± 6,97; 40)*	45,88 ( ± 7,42; 35)*	32,62 ( ± 4,86; 39)*	—
	Gal	100,00 ( ± 6,04; 36)	108,54 ( ± 5,23; 30)	134,42 ( ± 8,75; 16)*	61,68 ( ± 3,46; 9)*	**93,08 ( ± 7,95; 15)**	—
	GalNAc	100,00 ( ± 6,04; 36)	91,01 ( ± 5,12; 24)	87,39 ( ± 5,05; 13)	120,64 ( ± 12,25; 15)	80,13 ( ± 3,41; 29)	—
**NS-IgM (S2)**	Glc	100,00 ( ± 4,6; 67)	—	—	26,16 ( ± 5,28; 31)*	0,52 ( ± 0,28; 30)*	0,00 ( ± 0; 31)*
	Fru	100,00 ( ± 4,87; 42)	—	—	63,71 ( ± 4,89; 30)*	62,85 ( ± 3,29; 29)*	19,28 ( ± 4,25; 23)*
	Man	100,00 ( ± 5,57; 44)	—	—	91,43 ( ± 3,76; 46)	46,98 ( ± 3,79; 36)*	13,63 ( ± 1,42; 58)*
	GlcNAc	100,00 ( ± 4,6; 67)	—	—	61,76 ( ± 4,89; 25)*	48,41 ( ± 2,47; 25)*	28,41 ( ± 4,9; 18)*
	Gal	100,00 ( ± 4,59; 86)	—	—	85,13 ( ± 3,71; 64)	84,09 ( ± 3,07; 52)	58,62 ( ± 2,78; 56)*
	GalNAc	100,00 ( ± 4,59; 86)	—	—	92,33 ( ± 4,94; 77)	86,33 ( ± 5,03; 55)	68,45 ( ± 4,8; 58)*

**Table 1**. Mean values of CA fluorescence when stained with ConA, α-p62, MS-IgM and NS-IgM and previously preadsorbed with the indicated sugars at the indicated concentrations. For each sugar assay, the values are expressed as RI%, i.e. percentage respect to the mean value of fluorescence obtained in 0 mM, ( ± s.e.m.; N). * indicates significant differences respect to 0 mM (p < 0,01). ** indicates a significant value considered outlier (see text). — indicates not tested. **S1** and **S2** indicate different series. Note that in the case of ConA, all sugars produce a decrease of the CA staining, except Gal and GalNAc. For α-p62, none of the tested sugars interfere with the staining, indicating that the staining of this protein is not affected by the sugar preadsorption. Contrarily, all tested sugars interfere with the staining with the MS-IgM, reaching the mean RI% values near to 0% at 400 mM. In the case of NS-IgM, Glc is the sugar that produces the highest decrease, while Gal and GalNAc only produce a significant decrease at 800 mM, producing a lower than 42% reduction in RI%. Bold values are the significant ones, to improve visualization.

**Figure 7A** presents a graphical view of these data, showing the different dynamics for Gal and GalNAc with respect to the other sugars. These results were expected according to the carbohydrate-binding domain of ConA, indicating that sugar preadsorption could be an optimal method for studying the nature of the interaction. This figure contains the summary of all the results of the section, and is located at the end of the section.

#### Effects of Sugar Preadsorption on α-p62 Staining of CA

Before using the preadsorption with sugars to study the interaction between IgMs and CA, we applied the preadsorption method to analyze the interaction between the α-p62 antibody and CA. As the α-p62 antibody stains the protein p62, it was expected that preadsorption with the different sugars would not interfere with this immunostaining and, thus, α-p62 antibody staining would be used as a negative control to validate the sugar preadsorption method.

**Figure 2:**
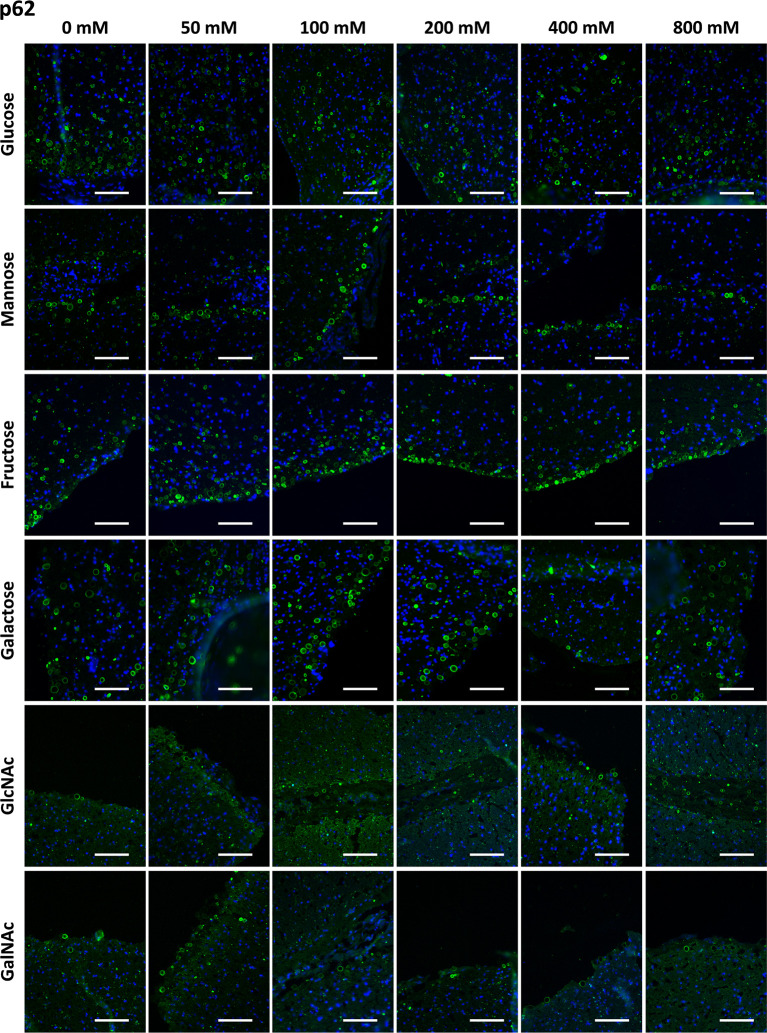
Representative images of hippocampal sections stained with the α-p62 antibody (green) and Hoechst (blue). Each row corresponds to a preadsorption study performed with the indicated carbohydrate. Sugar concentrations are indicated above. The staining of CA (green) is always present for all the sugars tested and at all the sugar concentrations. Scale bar: 100 μm.

**Figure 2** shows representative images of CA stained with the α-p62 antibody, previously preadsorbed with Glc, Man, Fru, Gal, GlcNAc and GalNAc at different concentrations ranging from 0 mM to 800 mM. As can be observed, the staining was maintained at all the sugar concentrations tested, indicating that the presence of the different sugars did not affect the α-p62 antibody staining.

**Table 1** presents the mean RI% values obtained from the different p62 assays. The values ranged from 53.63% to 127%, with no significant differences compared to the values obtained at 0 mM (p < 0.01 in all cases). Moreover, the graphical view of these data (**Figure 7B**) shows no downward trends in relation to the sugar concentration for any of the four sugars tested.

#### Effects of Sugar Preadsorption on IgMs Staining of CA

As the preadsorption method used was reliable in describing the sugar-based interaction between ConA and CA and given that the staining of CA with antibodies directed against proteins was not affected by the sugar preadsorption, we applied the preadsorption method to analyze if sugars interfered with the interaction between IgMs and CA. We used two different sources of IgMs: those obtained from myeloma serum (MS-IgMs) and those obtained from normal human serum (NS-IgMs).

In the case of MS-IgMs, we tested the preadsorption of IgMs with Glc, Gal, Fru and Man at concentrations ranging from 0 mM to 400 mM. As it was necessary to replicate the assay with the NS-IgMs (see below), the experiment with MS-IgMs was also replicated in order to analyze the robustness of the results. Thus, the preadsorption of MS-IgMs included two series, **S1** and **S2**.

**Figure 3:**
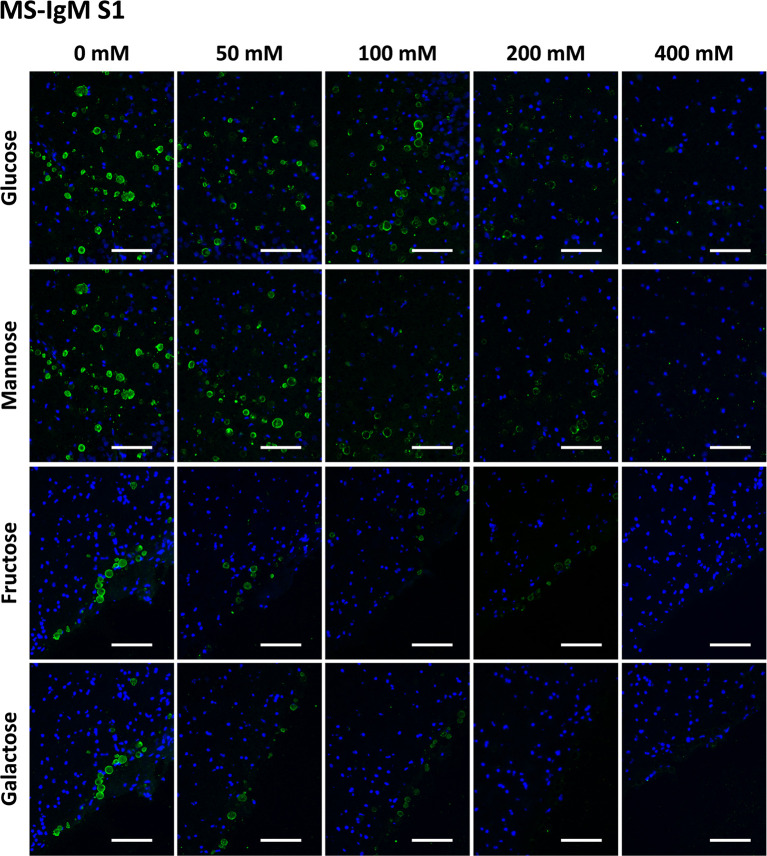
Representative images of hippocampal sections stained with MS-IgMs and Hoechst (blue) obtained in the first series (**S1**). Each row corresponds to a preadsorption study performed with the indicated carbohydrate. Sugar concentrations are indicated above. Note that the staining of CA becomes weaker with increasing sugar concentrations and even disappears. Scale bar: 100 μm.

**Figure 4:**
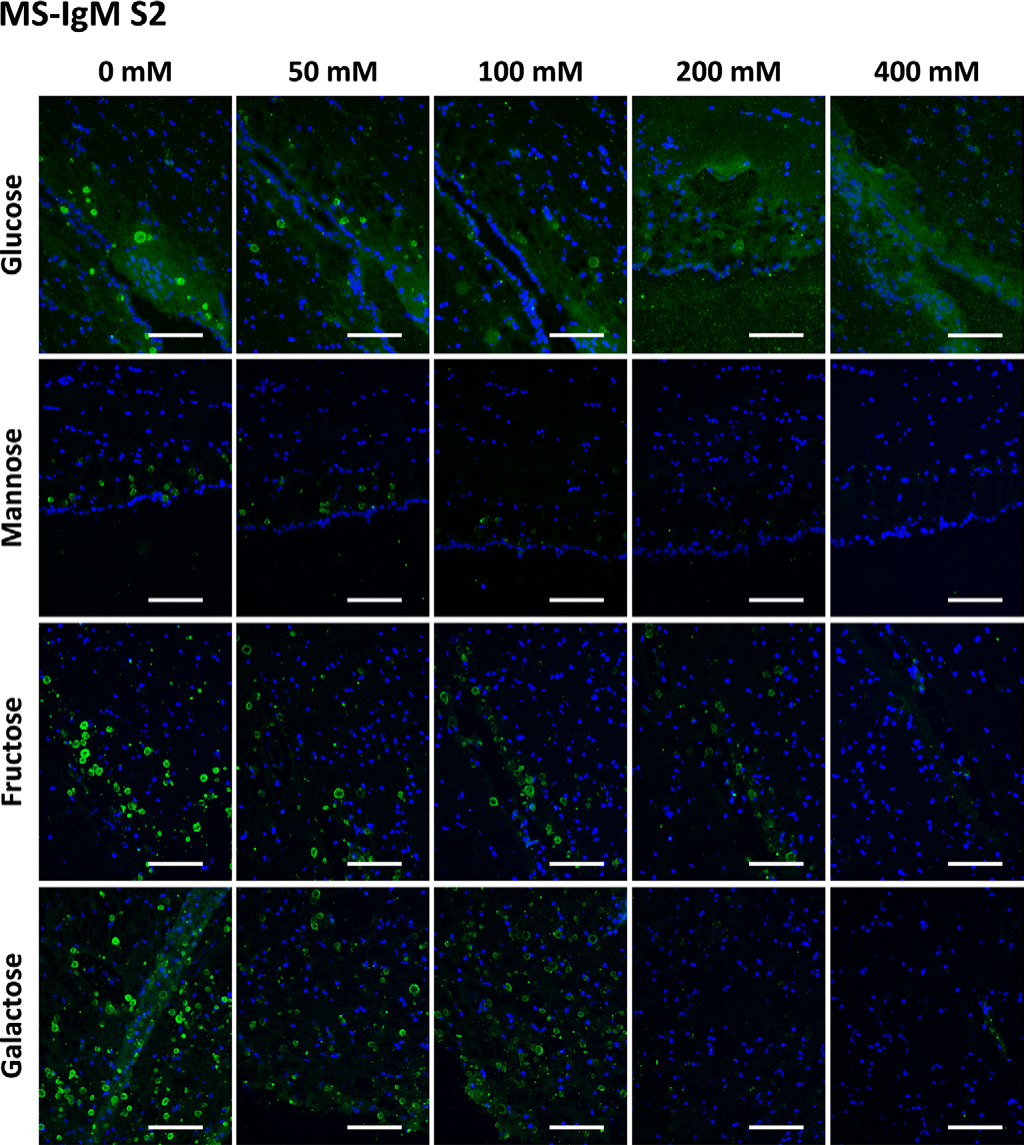
Representative images of hippocampal sections stained with MS-IgMs and Hoechst (blue) obtained in the second series (**S2**). Each row corresponds to a preadsorption study performed with the indicated carbohydrate. Sugar concentrations are indicated above. As observed in **Figure S3**, the staining of CA becomes weaker with increasing sugar concentrations and even disappears. Scale bar: 100 μm.

**Figures 3** and **4** show representative images of the staining of CA with MS-IgMs for each sugar at each concentration, corresponding to **S1** and **S2**, respectively. In the absence of sugars, i.e. the positive controls of the staining, the staining of CA can be appreciated. When increasing the sugar concentration, the staining becomes weaker or even disappears.

**Table 1** shows the mean RI% values for each experimental condition, including the results from **S1** and **S2**. Statistical significances with respect to the values obtained at 0 mM are indicated. As can be observed, all the sugars interfered with the staining, with the sugar concentrations of 200 mM and 400 mM producing a significant decrease. Moreover, as can be observed in the graphical view of these data (**Figures 7C, D** corresponding to **S1** and **S2**, respectively), there was a gradual decrease in staining when the sugar concentration increased. Although there were some discrepancies when comparing **S1** and **S2** (see, for example, Man at 50 and 100 mM), the general trends observed in **S1** were the same as those observed in **S2**. Furthermore, the downward trends were similar for the different sugars, with the RI% values at 400 mM close to 0%. Thus, the results obtained with MS-IgMs indicated that the interaction between the MS-IgMs and CA was affected by all the sugars tested.

**Figure 5:**
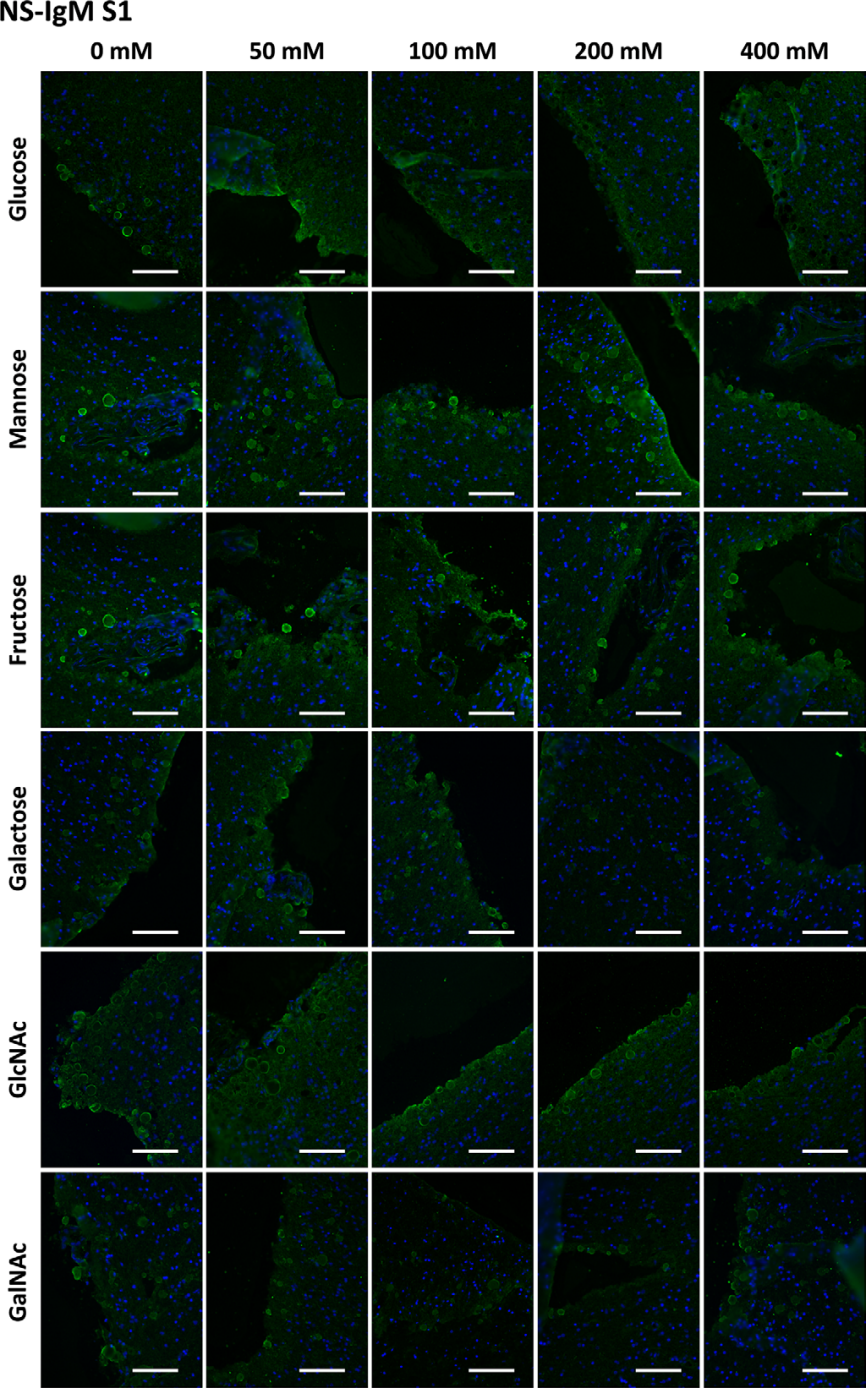
Representative images of hippocampal sections stained with NS-IgMs and Hoechst (blue) obtained in the first series (**S1**). Each row corresponds to a preadsorption study performed with the indicated carbohydrate. Sugar concentrations are indicated above. Scale bar: 100 μm.

**Figure 6:**
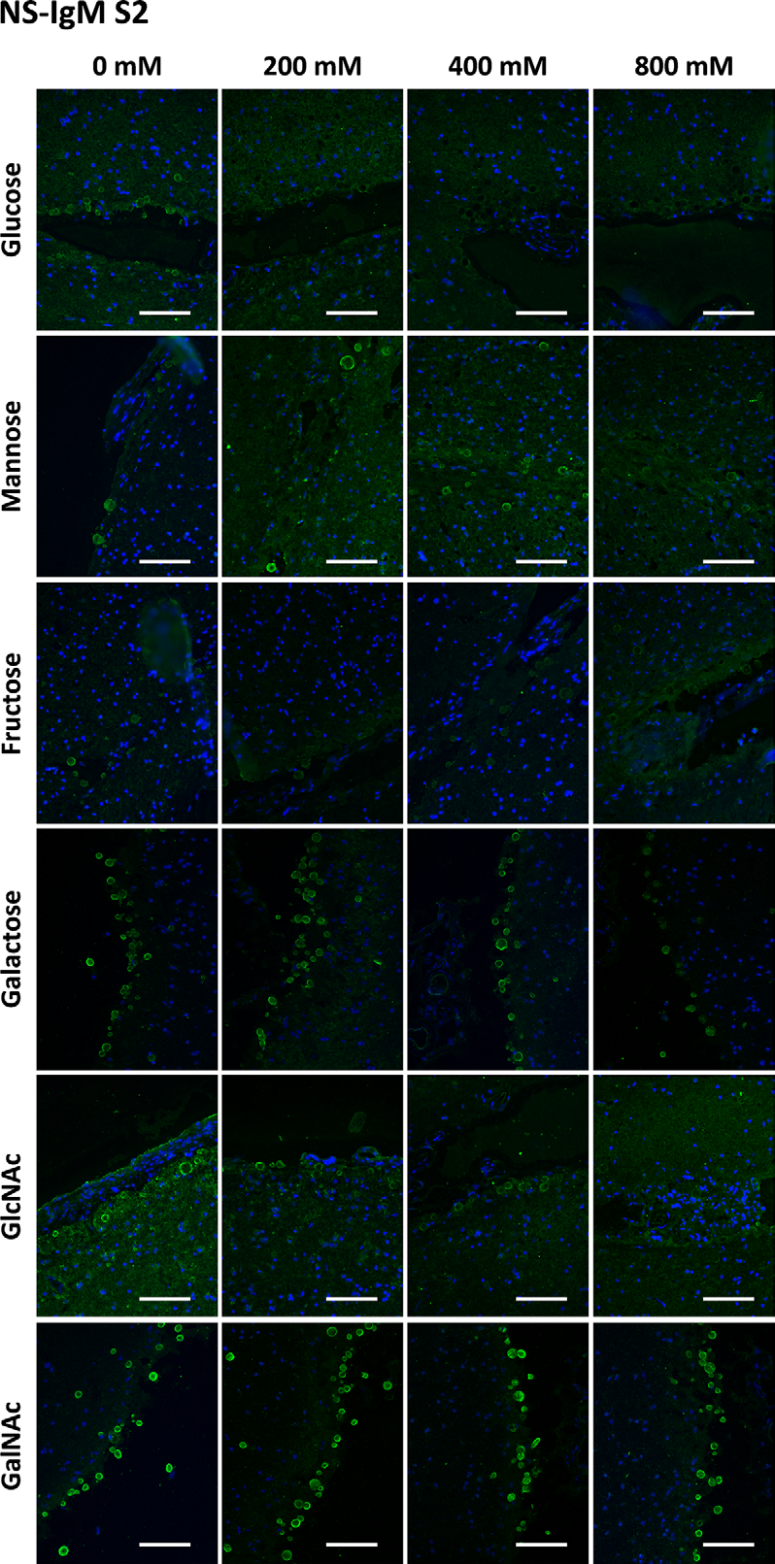
Representative images of hippocampal sections stained with NS-IgMs and Hoechst (blue) obtained in the second series (**S2**). Each row corresponds to a preadsorption study performed with the indicated carbohydrate. Sugar concentrations are indicated above. Scale bar: 100 μm.

In the case of NS-IgMs, we first tested the preadsorption with Glc, Gal, Fru, Man, GlcNAc and GalNAc at concentrations ranging from 0 mM to 400 mM (**S1**). After considering the need to include the concentration of 800 mM, we performed a second series (**S2**) with all the sugars at concentrations of 0, 200, 400 and 800 mM. Representative images corresponding to **S1** and **S2** can be found in **Figures 5** and **6**.

**Figure 7:**
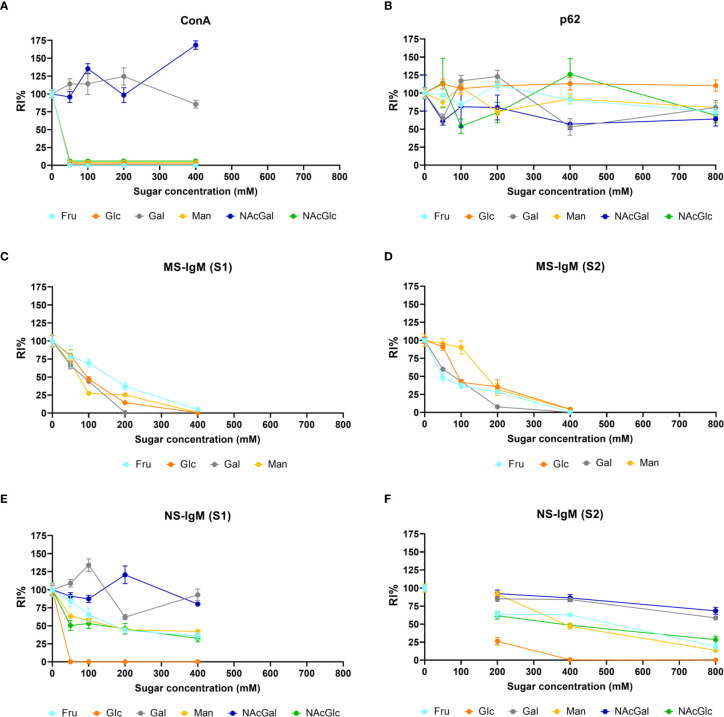
Graphical view of the RI% mean values (± s.e.m.) of fluorescence resulting from the staining of CA with ConA, α-p62 antibody, MS‐IgM or NS-IgM that have been preadsorbed with the indicated sugars at the indicated concentrations. **S1** and **S2** correspond to data from the different series. The data and significant differences are detailed in **Table 1**. Note in **(A)** the decrease produced by Glc, Fru, Man and GlcNAc, but not by Gal and GalNAc, in the ConA staining of CA. In **(B)** (corresponding to α-p62 antibody staining), note that the lines do not show the downward trend in relation to the sugar concentration for any of the four sugars tested. On the contrary, **(C, D)** show that staining with MS‐IgMs (**S1** and **S2**) is affected by the presence of the sugars. Staining with NS-IgMs **(E, F)** is also affected by the presence of the sugars. See text for details.

**Table 1** presents the mean RI% values from **S1** and **S2** and the statistical significances with respect to the values obtained at 0 mM. The graphical view of these data is presented in **Figures 7E, F**. As can be observed, the results from **S1** and **S2** were very similar. The different sugars seem to interfere in different ways with the staining of CA with NS-IgM. Glc had the largest effect on the staining, with the RI% close to 0% for practically all the tested concentrations, being significantly lower than the control values. Man, Fru and GlcNAc also decreased the RI%. However, this decrease seems to be not as pronounced as that produced by Glc, with values at 800 mM being around 20%. The results from the experiments with Gal and GalNAc appear to be different to those with the other sugars, with no clear trend observed. **Table 1** shows that significant differences compared to the control values were observed only for 800 mM of Gal or GalNAc. At this concentration, the RI% values were 58.62 for Gal and 68.45 for GalNAc, i.e., far from 0% and far from the values obtained with the other sugars at 800 mM. Thus, Glc showed the largest effect on the interaction between the NS-IgMs and CA, while Fru, Man and GlcNAc had an intermediate effect, and Gal and GalNAc had a minor effect. This indicates that there are some differences in the way in which MS-IgMs and NS-IgMs interact with CA, in terms of immunoreactivity. However, generally, the interaction between IgMs and CA seems to involve carbohydrates, suggesting that the NEs have a carbohydrate nature.

### Digestion Studies

As explained before, to exclude the possible peptidic composition of the NEs recognized by the IgMs, we checked the IgM staining of CA obtained from the CSF after their digestion with the peptidic enzyme pepsin. Staining with AF555 NHS ester probe, α-p62 antibody and fluorescein-labeled ConA were used as controls. After the digestion with pepsin, the AF555 NHS ester and α-p62 staining should not appear if the proteins have been digested, but the staining with ConA should not be affected, as ConA binds to carbohydrate structures.

**Figure 8:**
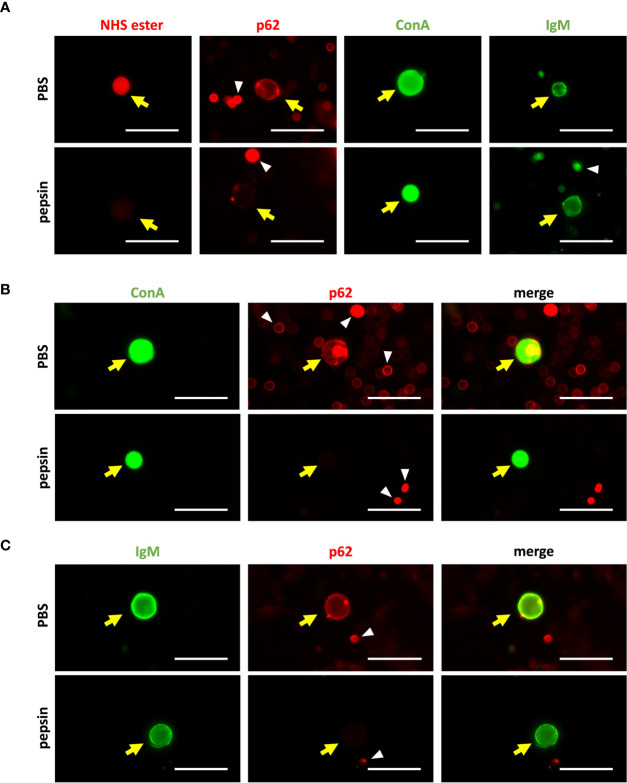
**(A)** Representative images of single staining of CA from CSF with AF555 NHS ester probe, α-p62 antibody, ConA or NS-IgM (left to right). The staining has been performed after the incubation with pepsin (upper row), or after the incubation with PBS (lower row; controls). The digestion prevented the staining with the AF555 NHS ester probe and the α-p62 antibody, but not the staining with ConA or NS-IgM. CA are indicated by yellow arrows, while some artifacts or cell debris are pointed with white arrowheads. **(B)** Double staining of CA with ConA and the α-p62 antibody. The staining has been performed after the incubation with pepsin (upper row), or after the incubation with PBS (lower row; controls). Left: green channel (ConA); center: red channel (p62); right: merged images. Pepsin eliminated the staining of CA with the α-p62 antibody, but not that with ConA. **(C)**: Double staining of CA with NS-IgM and the α-p62 antibody, without and with the pepsin digestion (upper and lower rows, respectively). Left: green channel (IgM); center: red channel (p62); right: merged images. Pepsin eliminated the staining of CA with the α-p62 antibody, but not that with NS-IgM. Scale bar: 50 μm.

First of all, we performed single staining of CA with NHS ester probe, α-p62, ConA and NS-IgM (**Figure 8A**). For each product, we used matching aliquots, one of them digested with pepsin and the other simply diluted in PBS. As expected, CA were stained with the NHS ester probe in the absence of pepsin digestion, but not when CA have been previously digested with pepsin, indicating that the digestion with pepsin, eliminated all the peptidic components of the CA. Accordingly, CA were stained with the α-p62 in absence of pepsin digestion, but not when CA have been previously digested. Staining with ConA was not affected by the pepsin digestion, indicating that the glucidic structure recognized by ConA was not affected by the peptidic enzyme. Finally, staining with NS-IgMs occurred with and without the pepsin digestion, indicating that the targets of the IgMs were not affected by this digestion and, thus, they do not have a peptidic nature.

Two double staining procedures were performed, using ConA and the α-p62 antibody in one case and NS-IgMs and α-p62 antibodies in the other. In the first case, CA were stained with both ConA and the α-p62 antibody in the absence of the pepsin digestion, while after the pepsin digestion the ConA staining can be observed but not that of p62 (**Figure 8B**). Similarly, pepsin digestion led to the disappearance of the α-p62 antibody staining, but did not affect NS-IgM staining in the double immunostaining with the α-p62 antibody and NS-IgMs (**Figure 8C**), indicating that the NEs recognized by the IgMs are not of a peptidic nature.

## Discussion

In previous studies, we had determined that CA in the human brain exhibit NEs that are recognized by plasma IgMs, which are in fact natural IgMs (10). The present study aimed to clarify the nature of these NEs, and we adapted standard techniques to this purpose.

Since the preadsorption of ConA with specific sugars interferes with the interaction between ConA and CA, and given that the sugar preadsorption of antibodies directed against proteins do not disrupt their interaction with CA, we used this strategy to study the interaction between IgMs and CA. We observed that the preadsorption of IgMs with specific carbohydrates had inhibitory effects. On the other hand, we found that the digestion of CA with pepsin, that eliminates their proteinaceous components, removes the staining with NHS ester probe and α-p62 while that of ConA is maintained, indicating that the sugar target of ConA has not been altered. We also observed that the pepsin digestion has no effect on IgM staining, thus excluding the peptidic nature of the NEs. Altogether, all these results strongly support that the NEs contained in CA have a carbohydrate nature.

Glycans have multiple complex functions, including acting as “eat-me” signals to induce phagocytosis by macrophages or dendritic cells, either directly or indirectly, for example, *via* opsonization with natural IgMs (46, 50). Recently, Xia and Gildersleeve, analyzing α-glycan IgM repertoires in newborn human cord blood, reported that many α-glycan antibodies present in human sera belong to the IgM class and are natural antibodies (49), with natural IgMs estimated to constitute between 80% and 90% of natural antibodies (46). Membrane-derived epitopes are among the NEs recognized by these antibodies (56). Such epitopes include carbohydrate determinants generally anchored to intracellular membranes that act as “eat-me” signals when exposed to the outside of the cell, for example, when apoptosis occurs or in cell debris (56). In fact, under physiological conditions, there are multiple elements inside cells that can act as “eat-me” signals when externalized. This is an energy-saving process for the body because in the case of functional or structural alterations, it is not necessary to generate these signals *de novo*, but it is enough to relocate them to the outside of the cell (56). For all these reasons, given that CA accumulate different residual elements and that they can act as waste containers that are part of the brain cleaning system, it would not be surprising that different species of NEs with a carbohydrate nature occur in CA. Some of these could participate in the phagocytosis of CA by macrophages, a process that has been observed in nerve tissue samples from cases of neuromyelitis optica (17) and in *in vitro* studies (24).

In the present study, we used two different pools of IgMs (NS-IgMs and MS-IgMs). The binding of the NS-IgMs to CA was affected to varying levels by the different sugars, with Glc having the largest effect, reducing the RI% to 0% at a concentration of 50 mM. Gal and GalNAc had the smallest effects, producing a slight decrease in staining only at the highest concentration tested (800 mM), when the RI% value was around 60%. This suggests that the equatorial arrangement of the hydroxyl group at C-4 in the monosaccharide is critical for its interaction with IgMs. Moreover, the equatorial or axial arrangement of the hydroxyl group at C-2 might also have some importance. This is illustrated by the lower inhibition exerted by Man compared to Glc and also in the comparison of the inhibitions produced by Glc and GlcNAc, in which the hydroxyl group at C-2 is substituted with NAc. The effects of the changes at C-2, however, are not as strong as those at C-4.

The four monosaccharides tested interfered with the binding of the MS-IgMs to CA, with no significant differences observed among the sugars. This different behavior of MS-IgMs compared to NS-IgMs could be due to the fact that the former come from an extract of myeloma serum and, therefore, this pool of IgMs is restricted and possibly altered with respect to the pool of IgMs found in normal serum (NS-IgMs). However, it should be noted that in both cases, the interaction between the IgMs and CA was inhibited by the sugars, further supporting the carbohydrate nature of the NEs of CA that were recognized by these IgMs. We do not know whether there is only one type of NE in CA or whether there is a collection of different NEs. In any case, the binding of the IgMs to these NEs of CA can be completely blocked by certain monosaccharides.

The present work indicates that the binding between certain human IgMs and certain antigens or epitopes present in human structures can be inhibited *in vitro* by certain monosaccharides, especially glucose. Therefore, we wondered whether these inhibitions also occur *in vivo*. In human sera, the IgM concentration in early adulthood is 1.46 ± 0.12 mg/ml (57), while the normal plasma glucose two hours after eating is about 7.8 mM (58). In our study, the NS-IgM concentration was 0.044 mg/ml, while the lowest glucose concentration used to preadsorb the NS-IgM was 50 mM or, more precisely, 48.1 mM if we take into account the addition of the antibody during the antibody dilution. Therefore, the Glc/IgM ratio in our experimental conditions was about 205 times higher than that in physiological conditions. In any case, taking into account that in our experimental conditions the RI% decreases to about 0% at 48.1 mM of Glc concentration, we may consider that physiological levels of Glc could also induce some inhibitory effects on IgM. In this sense, it must be pointed that short-term hyperglycemia influence the innate immune system (59). Furthermore, diabetes mellitus increases susceptibility to viral, bacterial and fungal infections through modulating the immune system (59–61). Although different mechanisms have been described to explain this, including the activation of protein kinase C and the glycosylation of some proteins (59), the possible inhibition of IgM reactivity by glucose or other sugars has not been investigated to date. Thus, the present work introduces a new focus of interest.

In summary, the present work indicates that the NEs in CA have a carbohydrate nature. Furthermore, it indicates that the natural IgMs that recognize these NEs can be blocked *in vitro* by preadsorption with different sugars, particularly glucose. Finally, our findings reveal the need to study the effects that glycemia produce *in vivo* on the reactivity of natural IgMs and, consequently, on natural immunity.

## Data Availability Statement

The original contributions presented in the study are included in the article/**Supplementary Material**. Further inquiries can be directed to the corresponding author.

## Ethics Statement

The studies involving human samples were reviewed and approved by Bioethical Committee of the Universitat de Barcelona. The ethics committee waived the requirement of written informed consent for participation.

## Author Contributions

MR, EA, CP, and JV designed research. MR, EA, IT, JDV, LM-P, TX, CP, and JV performed research. MR, EA, JDV, CP, and JV analyzed data. MR, EA, JDV, CP, and JV wrote the paper. All authors contributed to the article and approved the submitted version.

## Conflict of Interest

The authors declare that the research was conducted in the absence of any commercial or financial relationships that could be construed as a potential conflict of interest.
