# Supplementary material for: Corpora Amylacea in the Human Brain Exhibit Neoepitopes of a Carbohydrate Nature
Source: Front Immunol. 2021 Jun 28;12:618193. doi: 10.3389/fimmu.2021.618193 (PMC8273382; doi:10.3389/fimmu.2021.618193)
Supplement: Supplementary file 1 [file DataSheet_1.pdf]

Table S1

Gender	Age of death (years)	Post-mortem delay (hh:mm)	Neuropathological diagnosis[a]	Braak stage - CERAD
Female	85	5:00	AD	V - C
Male	88	3:00	AD	V - C
Male	91	4:30	AD + LBD + SVD	VI - C
Male	70	4:30	SVE	None
Male	80	10:00	SVE	None
Male	86	10:15	LBD	None

Medical data of the brain donors. [a]AD: Alzheimer's disease with a high level of neuropathological change (A3B3C3); LBD: Lewy body dementia; SVD: small vessel disease; SVE: subcortical vascular encephalopathy.

Table S2

Gender	Age of death (years)	Post-mortem delay (hh:mm)	Neuropathological diagnosis[a]
Female	65	6:15	HD, III
Male	74	10:34	LBD, PD
Male	92	6:20	Not affected

Medical data of the CSF donors. [a]HD: Huntington's disease; LBD: Lewy body dementia; PD: Parkinson's disease
