# Supplementary material for: Corpora Amylacea in the Human Brain Exhibit Neoepitopes of a Carbohydrate Nature
Source: Front Immunol. 2021 Jun 28;12:618193. doi: 10.3389/fimmu.2021.618193 (PMC8273382; doi:10.3389/fimmu.2021.618193)
Supplement: Supplementary file 2 [file DataSheet_2.pdf]

ConA

Glc	IM CA	IM BKG	INT-BKG	RI%
0	66,131	5,692	60,439	99,99628
0	60,83	8,418	52,412	86,71561
0	81,087	7,581	73,506	121,6156
0	62,966	7,542	55,424	91,69896
0	84,011	8,788	75,223	124,4564
0	66,32	7,485	58,835	97,34246
0	78,131	10,086	68,045	112,5804
0	70,457	4,881	65,576	108,4954
0	79,463	9,367	70,096	115,9738
0	59,685	5,814	53,871	89,12953
0	61,038	7,299	53,739	88,91113
0	71,541	8,78	62,761	103,838
0	74,574	6,179	68,395	113,1595
0	63,626	8,482	55,144	91,2357
0	76,49	9,972	66,518	110,054
0	60,72	7,585	53,135	87,91182
0	65,598	7,728	57,87	95,74587
0	64,89	7,813	57,077	94,43385
0	63,307	8,013	55,294	91,48388
0	54,553	9,088	45,465	75,22181
50	0,208	4,213	0	0
50	1,187	5,097	0	0
50	3,06	3,297	0	0
50	3,233	6,348	0	0
50	0,621	5,054	0	0
50	0,099	4,286	0	0
50	0,398	5,409	0	0
50	1,186	3,955	0	0
50	4,719	3,187	1,532	2,534693
50	0,17	3,543	0	0
50	2,77	3,636	0	0
50	2,595	5,219	0	0
50	0,727	4,267	0	0
50	2,502	3,591	0	0
50	1,309	4,197	0	0
50	2,46	5,435	0	0
50	3,296	5,95	0	0
50	3,386	5,244	0	0
50	3,159	4,987	0	0
50	0,475	6,332	0	0
50	3,719	4,8	0	0
50	2,463	5,877	0	0
50	3,806	6,847	0	0
50	0,194	4,312	0	0
100	0,155	4,637	0	0
100	1,36	3,867	0	0
100	0,105	4,026	0	0
100	1,629	4,263	0	0
100	1,642	3,323	0	0
100	0,595	3,603	0	0
100	0,584	4,657	0	0
100	1,182	3,201	0	0
100	1,07	3,705	0	0
100	1,006	2,203	0	0
100	2,054	4,851	0	0
100	1,119	5,46	0	0
100	1,354	3,669	0	0
100	1,44	4,904	0	0
200	0,14	1,717	0	0
200	0,211	1,676	0	0
200	1,136	2,549	0	0
200	0,052	2,547	0	0
200	0,887	2,883	0	0
200	1,044	2,486	0	0
200	0,838	2,603	0	0
200	0,086	2,192	0	0

p62

Glc	IM CA	IM BKG	CA-BKG	RI%
0	10,768	2,036	8,73	72,89
0	14,514	2,142	12,37	103,27
0	8,931	1,933	7,00	58,42
0	5,178	2,069	3,11	25,95
0	24,677	0,974	23,70	197,86
0	18,406	1,494	16,91	141,17
0	7,88	0,537	7,34	61,29
0	13,999	1,057	12,94	108,03
0	15,425	1,329	14,10	117,66
0	13,752	1,151	12,60	105,19
0	18,234	1,708	16,53	137,95
0	13,939	1,371	12,57	104,91
0	9,429	2,01	7,42	61,93
0	6,869	1,53	5,34	44,57
0	9,618	0,939	8,68	72,45
0	12,793	1,145	11,65	97,23
0	14,843	0,612	14,23	118,79
0	12,081	0,615	11,47	95,71
0	22,384	1,431	20,95	174,90
0	22,74	1,432	21,31	177,87
0	8,987	0,884	8,10	67,64
0	9,855	0,992	8,86	73,98
0	13,126	1,538	11,59	96,73
0	9,079	1,326	7,75	64,72
0	16,934	1,389	15,55	129,76
0	15,733	1,099	14,63	122,16
0	8,607	1,527	7,08	59,10
0	9,943	2,301	7,64	63,79
0	11,099	1,077	10,02	83,66
0	13,572	1,042	12,53	104,59
0	17,209	1,05	16,16	134,89
0	19,235	1,541	17,69	147,70
0	12,197	1,313	10,88	90,85
0	14,697	2,203	12,49	104,29
0	17,589	1,407	16,18	135,08
0	12,539	1,244	11,30	94,28
0	16,909	1,052	15,86	132,36
0	12,985	0,299	12,69	105,90
0	12,638	0,897	11,74	98,01
0	8,912	0,735	8,18	68,26
0	15,347	0,881	14,47	120,75
0	10,375	0,939	9,44	78,77
0	8,323	1,16	7,16	59,79
0	8,747	0,894	7,85	65,55
0	7,355	0,555	6,80	56,76
0	11,6	0,856	10,74	89,68
0	10,636	0,618	10,02	83,62
0	7,171	1,067	6,10	50,95
0	16,862	1,147	15,72	131,18
0	7,419	1,266	6,15	51,36
0	8,367	0,956	7,41	61,86
0	26,837	0,96	25,88	216,01
0	16,546	1,071	15,48	129,18
0	18,617	0,798	17,82	148,74
50	19,526	0,946	18,58	155,09
50	12,063	0,887	11,18	93,29
50	15,017	0,791	14,23	118,75
50	16,717	1,043	15,67	130,84
50	15,139	0,957	14,18	118,38
50	13,458	0,836	12,62	105,36
50	7,061	0,842	6,22	51,91
50	16,742	0,913	15,83	132,13
50	24,795	1,186	23,61	197,07
50	13,532	0,613	12,92	107,84
50	21,812	1,034	20,78	173,44
50	7,275	0,844	6,43	53,68

MS-IgM S1

Glc	IM CA	IM BKG	IM-BKG	RI%
0	53,691	2,576	51,115	136,163
0	46,883	2,241	44,642	118,919
0	11,116	4,290	6,826	18,183
0	49,494	2,778	46,716	124,444
0	49,184	2,282	46,902	124,940
0	30,876	2,465	28,411	75,683
0	46,194	2,055	44,139	117,580
0	58,717	2,136	56,581	150,723
0	18,682	2,332	16,350	43,554
0	55,152	1,797	53,355	142,130
0	57,327	2,805	54,522	145,238
0	39,978	3,213	36,765	97,936
0	46,261	1,781	44,480	118,488
0	58,868	2,657	56,211	149,738
0	41,717	2,531	39,186	104,386
0	40,281	2,464	37,817	100,739
0	47,342	2,512	44,830	119,420
0	32,643	2,360	30,283	80,669
0	29,814	1,748	28,066	74,764
0	52,459	1,743	50,716	135,100
0	42,216	2,491	39,725	105,821
0	14,467	2,723	11,744	31,284
0	16,367	1,922	14,445	38,479
0	11,444	3,010	8,434	22,467
0	37,952	2,610	35,342	94,146
0	51,358	2,043	49,315	131,368
0	55,903	2,178	53,725	143,115
0	47,875	2,763	45,112	120,171
0	15,278	2,382	12,896	34,353
50	23,482	2,700	20,782	55,360
50	34,889	2,560	32,329	86,120
50	20,164	1,729	18,435	49,108
50	32,812	2,736	30,076	80,118
50	53,798	2,601	51,197	136,381
50	18,574	3,239	15,335	40,850
50	50,883	2,749	48,134	128,222
50	11,250	2,390	8,860	23,602
50	21,074	2,706	18,368	48,930
50	33,617	1,994	31,623	84,239
50	38,299	2,305	35,994	95,883
50	56,380	2,365	54,015	143,888
50	46,511	2,022	44,489	118,512
50	36,281	2,738	33,543	89,353
50	38,323	2,061	36,262	96,596
50	34,575	2,516	32,059	85,400
50	24,317	2,273	22,044	58,722
50	35,417	1,392	34,025	90,637
50	24,997	1,595	23,402	62,339
50	13,505	1,764	11,741	31,276
100	12,536	2,459	10,077	26,844
100	33,181	2,772	30,409	81,005
100	21,419	4,745	16,674	44,417
100	27,408	3,188	24,220	64,518
100	16,528	2,975	13,553	36,103
100	26,635	2,230	24,405	65,011
100	17,580	2,596	14,984	39,915
100	29,737	2,802	26,935	71,751
100	10,864	2,441	8,423	22,438
100	12,936	3,174	9,762	26,004
100	21,923	2,689	19,234	51,236
100	13,160	2,661	10,499	27,968
100	13,067	3,102	9,965	26,545
100	27,809	2,966	24,843	66,178
100	30,818	2,882	27,936	74,417
100	22,673	3,556	19,117	50,925
100	35,206	2,915	32,291	86,018

MS-IgM S2

Glc	IM CA	IM BKG	IF-BKG	RI%
0	42,89	20,818	22,07	53,04
0	66,848	19,011	47,84	114,96
0	65,844	22,485	43,36	104,20
0	58,793	21,336	37,46	90,02
0	58,983	18,518	40,47	97,24
0	62,812	18,23	44,58	107,14
0	42,706	14,627	28,08	67,48
0	64,092	9,5	54,59	131,19
0	68,442	11,805	56,64	136,11
0	57,845	9,712	48,13	115,67
0	57,457	8,147	49,31	118,50
0	57,423	12,102	45,32	108,91
0	49,986	20,586	29,40	70,65
0	49,264	15,638	33,63	80,81
0	61,153	12,804	48,35	116,19
0	55,8	14,363	41,44	99,58
0	65,206	20,836	44,37	106,63
0	65,883	14,614	51,27	123,21
0	69,468	16,476	52,99	127,35
0	67,859	14,387	53,47	128,50
0	51,098	18,884	32,21	77,42
0	51,849	23,973	27,88	66,99
0	63,535	20,51	43,03	103,40
0	44,872	19,259	25,61	61,55
0	58,737	21,045	37,69	90,58
0	64,185	21,46	42,73	102,68
50	35,196	5,282	29,91	71,89
50	40,582	8,801	31,78	76,38
50	52,044	11,232	40,81	98,08
50	47,489	16,174	31,32	75,26
50	55,228	12,642	42,59	102,34
50	44,514	15,234	29,28	70,36
50	57,525	16,59	40,94	98,37
50	59,843	20,05	39,79	95,63
50	62,097	13,481	48,62	116,83
50	63,635	17,886	45,75	109,94
50	54,894	18,481	36,41	87,51
100	30,832	9,647	21,19	50,91
100	27,244	10,372	16,87	40,55
100	38,833	18,757	20,08	48,25
100	26,086	9,784	16,30	39,18
100	26,49	17,328	9,16	22,02
100	32,685	20,099	12,59	30,25
100	40,027	20,902	19,13	45,96
100	24,601	9,66	14,94	35,91
100	48,796	22,614	26,18	62,92
200	33,939	15,096	18,84	45,28
200	26,956	16,164	10,79	25,94
400	24,788	21,887	2,90	6,97
400	24,025	21,497	2,53	6,08
400	21,689	23,707	0,00	0,00
400	20,414	24,031	0,00	0,00
400	23,441	13,136	10,31	24,76
400	20,643	20,734	0,00	0,00
400	17,07	24,018	0,00	0,00
400	12,716	15,225	0,00	0,00
400	14,179	12,029	2,15	5,17
400	12,165	14,355	0,00	0,00

Man	IM CA	IM BKG	IF-BKG	RI%
0	31,312	1,98	29,33	140,14
0	28,721	2,381	26,34	125,85
0	14,761	3,453	11,31	54,03
0	11,482	3,374	8,11	38,74
0	39,089	2,142	36,95	176,53
0	25,708	1,967	23,74	113,43

NS-IgM S1

Man	RI	BKG	RI-BKG	RI%
0	30,211	9,508	20,703	85,377
0	43,788	8,866	34,922	144,015
0	30,246	12,723	17,523	72,263
0	40,556	9,824	30,732	126,736
0	39,992	8,340	31,652	130,530
0	32,353	10,294	22,059	90,969
0	32,061	12,021	20,040	82,643
0	29,637	14,066	15,571	64,213
0	51,488	26,450	25,038	103,254
50	25,330	10,741	14,589	60,164
50	20,269	10,409	9,860	40,662
50	21,077	8,382	12,695	52,353
50	22,540	7,518	15,022	61,949
50	14,944	6,684	8,260	34,063
50	21,183	8,174	13,009	53,648
50	21,343	7,781	13,562	55,928
50	24,215	8,360	15,855	65,384
50	23,646	6,933	16,713	68,923
50	24,332	9,129	15,203	62,696
50	25,158	7,076	18,082	74,568
50	22,238	7,996	14,242	58,733
50	23,944	7,202	16,742	69,042
50	23,685	6,953	16,732	69,001
50	20,385	7,828	12,557	51,784
50	22,820	7,843	14,977	61,764
50	25,296	8,064	17,232	71,063
50	34,922	15,869	19,053	78,573
50	39,862	19,105	20,757	85,600
50	36,719	17,976	18,743	77,294
50	32,677	13,728	18,949	78,144
100	24,874	9,467	15,407	63,537
100	29,576	16,540	13,036	53,759
100	25,581	11,715	13,866	57,182
100	25,859	16,038	9,821	40,501
100	34,133	12,249	21,884	90,247
100	29,194	10,753	18,441	76,049
100	32,269	15,642	16,627	68,568
100	24,429	9,951	14,478	59,706
100	24,482	11,681	12,801	52,790
100	25,083	13,047	12,036	49,635
100	23,949	10,395	13,554	55,895
100	20,409	8,074	12,335	50,868
100	24,948	14,207	10,741	44,295
100	23,935	8,884	15,051	62,069
100	25,803	10,525	15,278	63,005
100	20,262	11,966	8,296	34,212
100	19,494	9,395	10,099	41,647
100	37,050	21,007	16,043	66,160
100	24,399	15,648	8,751	36,088
100	18,538	7,816	10,722	44,216
100	16,855	7,252	9,603	39,602
100	15,475	8,346	7,129	29,399
100	18,581	8,691	9,890	40,785
100	20,496	7,950	12,546	51,738
100	19,078	11,833	7,245	29,878
100	24,222	8,921	15,301	63,100
100	21,308	11,462	9,846	40,604
100	17,176	11,314	5,862	24,174
100	19,960	16,033	3,927	16,195
100	23,978	16,597	7,381	30,439
100	27,042	10,858	16,184	66,741
100	27,588	10,427	17,161	70,770
100	19,279	9,244	10,035	41,383
100	24,305	13,986	10,319	42,555
100	26,910	9,903	17,007	70,135
100	21,407	11,558	9,849	40,616

NS-IgM S2

Man	RI	BKG	RI-BKG	RI%
0	23,014	4,847	18,167	71,162
0	20,818	3,972	16,846	65,988
0	25,101	5,012	20,089	78,691
0	26,126	3,174	22,952	89,906
0	32,477	3,785	28,692	112,390
0	23,048	4,869	18,179	71,209
0	25,058	3,146	21,912	85,832
0	28,458	4,573	23,885	93,560
0	17,537	4,257	13,280	52,019
0	35,078	5,991	29,087	113,937
0	29,407	4,844	24,563	96,216
0	42,262	6,686	35,576	139,355
0	32,736	5,096	27,640	108,269
0	20,168	5,144	15,024	58,851
0	24,270	6,363	17,907	70,144
0	22,138	4,940	17,198	67,367
0	31,672	2,565	29,107	114,016
0	29,749	4,347	25,402	99,503
0	25,648	2,527	23,121	90,568
0	46,430	6,274	40,156	157,296
0	36,569	5,448	31,121	121,905
0	22,688	5,328	17,360	68,001
0	30,057	6,141	23,916	93,682
0	28,182	5,327	22,855	89,526
0	26,035	5,640	20,395	79,890
0	15,328	5,060	10,268	40,221
0	35,057	6,293	28,764	112,672
0	23,106	5,557	17,549	68,741
0	33,003	5,912	27,091	106,119
0	41,746	7,551	34,195	133,946
0	32,642	7,974	24,668	96,627
0	51,114	5,613	45,501	178,233
0	33,227	6,052	27,175	106,448
0	53,937	5,525	48,412	189,635
0	18,563	4,743	13,820	54,135
0	28,165	4,358	23,807	93,255
0	22,631	3,808	18,823	73,732
0	24,531	6,642	17,889	70,073
0	30,564	6,890	23,674	92,734
0	43,895	6,225	37,670	147,558
0	24,028	6,785	17,243	67,543
0	59,750	6,799	52,951	207,415
0	38,596	7,013	31,583	123,714
0	44,359	6,597	37,762	147,918
200	31,183	12,708	18,475	72,369
200	46,786	14,391	32,395	126,895
200	35,965	16,799	19,166	75,075
200	26,911	13,564	13,347	52,282
200	56,454	20,618	35,836	140,374
200	52,766	18,579	34,187	133,914
200	50,266	23,616	26,650	104,391
200	52,788	16,099	36,689	143,715
200	45,681	15,179	30,502	119,480
200	45,057	18,899	26,158	102,464
200	53,574	19,844	33,730	132,124
200	42,457	14,005	28,452	111,450
200	45,104	14,592	30,512	119,519
200	42,799	9,200	33,599	131,611
200	31,900	13,724	18,176	71,198
200	35,931	15,949	19,982	78,272
200	36,969	11,612	25,357	99,326
200	33,672	17,334	16,338	63,998
200	30,163	16,243	13,920	54,526
200	40,752	13,034	27,718	108,575
200	36,880	13,512	23,368	91,535
200	36,611	10,260	26,351	103,220

200	0,006	2,775	0	0
200	0,224	2,973	0	0
200	0,035	2,042	0	0
200	0,313	2,477	0	0
200	0,003	2,165	0	0
200	0,176	2,736	0	0
200	0,033	2,115	0	0
200	2,359	1,956	0,403	0,666763
400	0,062	2,269	0	0
400	1,366	2,535	0	0
400	0,514	2,733	0	0
400	0,626	2,086	0	0
400	0,176	3,11	0	0
400	0,027	3,288	0	0
400	1,565	2,656	0	0
400	0,97	3,024	0	0
400	0,939	1,681	0	0
400	1,913	3,137	0	0
400	0,514	3,167	0	0
400	0,335	3,052	0	0
400	0,551	3,634	0	0
400	0,101	3,479	0	0
400	0,61	2,62	0	0
400	1,083	3,175	0	0
400	0,819	2,905	0	0
400	1,125	3,799	0	0
400	0,925	2,934	0	0

Man	IM CA	IM BKG	INT-BKG	RI%
0	66,131	5,692	60,439	99,99628
0	60,83	8,418	52,412	86,71561
0	81,087	7,581	73,506	121,6156
0	62,966	7,542	55,424	91,69896
0	84,011	8,788	75,223	124,4564
0	66,32	7,485	58,835	97,34246
0	78,131	10,086	68,045	112,5804
0	70,457	4,881	65,576	108,4954
0	79,463	9,367	70,096	115,9738
0	59,685	5,814	53,871	89,12953
0	61,038	7,299	53,739	88,91113
0	71,541	8,78	62,761	103,838
0	74,574	6,179	68,395	113,1595
0	63,626	8,482	55,144	91,2357
0	76,49	9,972	66,518	110,054
0	60,72	7,585	53,135	87,91182
0	65,598	7,728	57,87	95,74587
0	64,89	7,813	57,077	94,43385
0	63,307	8,013	55,294	91,48388
0	54,553	9,088	45,465	75,22181
50	1	3,704	0	0
50	0,125	2,873	0	0
50	0,698	3,206	0	0
50	1,297	2,513	0	0
50	0,131	3,279	0	0
50	2,565	3,196	0	0
50	3,701	3,491	0,21	0,347445
50	0,411	3,147	0	0
50	0,027	3,702	0	0
50	1,088	3,444	0	0
50	0,345	3,258	0	0
50	1,613	4,172	0	0
50	1,398	3,86	0	0
50	1,121	4,665	0	0
50	0,338	4,172	0	0
50	1,196	3,275	0	0
50	0,013	3,771	0	0
50	0,434	5,042	0	0
50	1,006	5,313	0	0
50	0,947	3,241	0	0
50	1,026	3,229	0	0
50	0,621	2,767	0	0

50	8,748	0,873	7,88	65,74
50	10,187	0,872	9,32	77,76
50	29,089	0,745	28,34	236,60
50	10,838	0,082	10,76	89,78
50	9,811	0,759	9,05	75,56
50	25,424	0,766	24,66	205,83
50	11,044	0,956	10,09	84,21
50	16,032	0,907	15,13	126,25
50	13,914	0,758	13,16	109,82
50	16,664	1,019	15,65	130,60
50	11,395	0,906	10,49	87,56
50	13,037	0,949	12,09	100,90
50	11,982	1,011	10,97	91,58
50	17,609	1,067	16,54	138,08
50	28,509	1,079	27,43	228,97
50	14,704	0,949	13,76	114,82
50	10,934	0,961	9,97	83,25
50	13,962	1,053	12,91	107,76
50	28,094	1,355	26,74	223,20
50	18,301	0,924	17,38	145,05
50	12,662	0,913	11,75	98,07
50	10,622	0,897	9,73	81,18
50	18,735	1,16	17,58	146,71
50	26,511	2,612	23,90	199,49
50	10,322	0,937	9,39	78,34
50	25,917	1,017	24,90	207,85
50	13,395	1,03	12,37	103,22
50	26,949	1,239	25,71	214,61
50	11,192	1,392	9,80	81,80
50	10,751	0,982	9,77	81,55
50	8,393	0,725	7,67	64,01
50	16,35	0,94	15,41	128,63
50	8,919	1,052	7,87	65,67
50	8,718	0,668	8,05	67,20
50	5,976	1,073	4,90	40,93
50	13,932	0,856	13,08	109,15
50	8,161	0,95	7,21	60,19
50	8,927	1,045	7,88	65,79
50	7,726	0,944	6,78	56,61
50	6,634	0,677	5,96	49,73
50	5,773	0,885	4,89	40,80
50	9,357	0,953	8,40	70,15
50	18,557	0,918	17,64	147,24
50	7,678	0,769	6,91	57,67
50	18,415	0,823	17,59	146,85
100	17,278	1,063	16,22	135,35
100	13,796	1,048	12,75	106,41
100	9,009	1,02	7,99	66,69
100	6,192	0,837	5,36	44,70
100	12,97	0,752	12,22	101,99
100	11,607	0,736	10,87	90,74
100	16,653	0,977	15,68	130,85
100	11,905	0,761	11,14	93,02
100	22,676	0,879	21,80	181,95
100	15,356	0,963	14,39	120,14
100	17,682	0,93	16,75	139,84
100	10,996	0,953	10,04	83,83
100	15,468	0,99	14,48	120,85
100	20,521	0,998	19,52	162,97
100	17,955	1,216	16,74	139,73
100	18,925	1,191	17,73	148,03
100	17,046	1,04	16,01	133,61
100	5,46	1,142	4,32	36,04
100	25,381	2,418	22,96	191,68
100	13,798	2,224	11,57	96,61
100	26,84	2,627	24,21	202,12
100	22,641	3,144	19,50	162,75
100	13,466	1,733	11,73	97,94
100	11,008	1,469	9,54	79,63
100	7,406	0,983	6,42	53,62
100	9,057	1,195	7,86	65,63

100	15,156	3,218	11,938	31,801
100	22,585	3,245	19,340	51,519
100	10,562	3,100	7,462	19,878
100	13,625	2,485	11,140	29,675
100	17,329	1,995	15,334	40,847
100	20,533	3,000	17,533	46,705
100	14,164	3,158	11,006	29,318
100	23,852	2,602	21,250	56,607
100	38,183	2,530	35,653	94,974
100	33,442	2,175	31,267	83,291
100	19,976	3,290	16,686	44,449
100	22,691	2,616	20,075	53,477
100	18,931	2,730	16,201	43,157
100	17,501	1,278	16,223	43,216
100	13,676	2,658	11,018	29,350
100	9,079	2,825	6,254	16,660
100	10,713	2,964	7,749	20,642
200	9,442	3,970	5,472	14,577
200	13,095	2,223	10,872	28,961
200	9,924	2,179	7,745	20,631
200	6,562	2,101	4,461	11,883
200	11,197	2,083	9,114	24,278
200	12,470	1,993	10,477	27,909
200	10,102	1,982	8,120	21,630
200	5,544	2,545	2,999	7,989
200	6,722	2,576	4,146	11,044
200	8,262	2,295	5,967	15,895
200	7,672	2,658	5,014	13,357
200	5,927	2,566	3,361	8,953
200	7,965	2,701	5,264	14,022
200	4,604	2,565	2,039	5,432
200	12,795	2,554	10,241	27,280
200	4,920	2,384	2,536	6,756
200	4,082	2,423	1,659	4,419
200	6,953	3,037	3,916	10,432
200	5,066	2,200	2,866	7,635
200	5,858	1,917	3,941	10,498
200	4,886	1,634	3,252	8,663
400	1,879	1,992	0,000	0,000
400	1,680	2,121	0,000	0,000
400	2,359	1,962	0,397	1,058
400	1,657	1,990	0,000	0,000
400	1,984	2,971	0,000	0,000
400	2,898	2,174	0,724	1,929
400	1,774	2,098	0,000	0,000
400	1,702	2,410	0,000	0,000
400	1,632	1,639	0,000	0,000

Man	IM CA	IM BKG	IM-BKG	RI%
0	53,691	2,576	51,115	136,163
0	46,883	2,241	44,642	118,919
0	11,116	4,290	6,826	18,183
0	49,494	2,778	46,716	124,444
0	49,184	2,282	46,902	124,940
0	30,876	2,465	28,411	75,683
0	46,194	2,055	44,139	117,580
0	58,717	2,136	56,581	150,723
0	18,682	2,332	16,350	43,554
0	55,152	1,797	53,355	142,130
0	57,327	2,805	54,522	145,238
0	39,978	3,213	36,765	97,936
0	46,261	1,781	44,480	118,488
0	58,868	2,657	56,211	149,738
0	41,717	2,531	39,186	104,386
0	40,281	2,464	37,817	100,739
0	47,342	2,512	44,830	119,420
0	32,643	2,360	30,283	80,669
0	29,814	1,748	28,066	74,764
0	52,459	1,743	50,716	135,100
0	42,216	2,491	39,725	105,821
0	14,467	2,723	11,744	31,284

0	36,566	1,457	35,11	167,75
0	8,718	3,523	5,20	24,82
0	22,297	3,136	19,16	91,55
0	34,058	3,084	30,97	147,99
0	6,87	2,897	3,97	18,98
0	9,191	3,16	6,03	28,82
0	10,777	6,512	4,27	20,38
0	11,264	7,559	3,71	17,70
0	14,58	4,917	9,66	46,17
0	22,105	4,168	17,94	85,70
0	11,257	5,537	5,72	27,33
0	35,597	3,332	32,27	154,16
0	35,706	5,28	30,43	145,37
0	26,754	5,974	20,78	99,28
0	37,045	6,372	30,67	146,55
0	12,217	1,986	10,23	48,88
0	27,479	7,37	20,11	96,08
0	22,077	4,413	17,66	84,40
0	17,86	3,33	14,53	69,42
0	50,111	3,274	46,84	223,78
0	27,562	2,551	25,01	119,50
0	17,771	3,467	14,30	68,34
0	39,926	3,64	36,29	173,37
0	31,216	3,468	27,75	132,58
0	25,203	3,557	21,65	103,42
0	28,188	3,093	25,10	119,90
0	24,363	3,264	21,10	100,81
0	34,563	3,893	30,67	146,54
0	36,76	3,89	32,87	157,05
0	25,377	2,272	23,11	110,39
0	16,494	4,166	12,33	58,90
0	34,937	3,786	31,15	148,83
0	15,947	2,016	13,93	66,56
50	44,056	5,601	38,46	183,73
50	23,769	7,055	16,71	79,86
50	46,364	9,217	37,15	177,48
50	29,222	5,853	23,37	111,65
50	26,315	5,923	20,39	97,43
50	32,416	6,835	25,58	122,22
50	25,453	7,018	18,44	88,08
50	23,454	4,844	18,61	88,92
50	34,599	4,572	30,03	143,46
50	16,342	4,585	11,76	56,17
50	26,315	4,333	21,98	105,03
50	9,57	5,447	4,12	19,70
50	19,522	5,079	14,44	69,01
50	20,539	3,463	17,08	81,59
50	25,468	3,987	21,48	102,63
50	11,766	3,473	8,29	39,62
50	12,374	6,087	6,29	30,04
50	8,652	4,974	3,68	17,57
50	13,31	3,611	9,70	46,34
50	33,04	1,357	31,68	151,38
50	15,714	3,093	12,62	60,30
50	26,635	2,414	24,22	115,72
50	14,052	2,011	12,04	57,53
50	29,183	4,107	25,08	119,81
50	28,714	2,463	26,25	125,42
50	27,341	2,55	24,79	118,45
50	30,531	2,998	27,53	131,55
50	25,011	3,159	21,85	104,41
50	23,349	3,217	20,13	96,19
50	32,801	3,18	29,62	141,52
50	22,281	2,854	19,43	92,82
50	24,923	2,394	22,53	107,64
50	19,57	2,261	17,31	82,70
50	19,856	3,261	16,60	79,29
100	18,59	1,033	17,56	83,88
100	13,784	2,718	11,07	52,87
100	18,477	2,433	16,04	76,66
100	25,821	2,505	23,32	111,40

100	39,656	12,774	26,882	110,859
100	39,116	13,067	26,049	107,423
100	32,666	14,051	18,615	76,766
100	35,547	14,177	21,370	88,128
100	33,221	9,665	23,556	97,143
100	32,519	12,578	19,941	82,235
100	35,368	11,741	23,627	97,435
200	34,561	23,031	11,530	47,549
200	26,455	12,008	14,447	59,578
200	25,249	11,969	13,280	54,765
200	24,699	14,886	9,813	40,468
200	12,858	6,663	6,195	25,548
200	24,907	14,641	10,266	42,336
200	14,554	9,573	4,981	20,541
200	19,097	11,774	7,323	30,199
200	21,510	11,969	9,541	39,346
200	28,407	16,951	11,456	47,243
200	21,182	9,287	11,895	49,054
200	29,839	16,667	13,172	54,320
200	20,301	10,923	9,378	38,674
200	19,647	10,154	9,493	39,148
200	24,007	11,252	12,755	52,600
200	28,479	16,379	12,100	49,899
200	25,851	9,257	16,594	68,432
400	26,524	10,429	16,095	66,374
400	18,581	6,190	12,391	51,099
400	19,570	10,149	9,421	38,851
400	17,292	7,152	10,140	41,816
400	20,692	9,352	11,340	46,765
400	21,663	9,312	12,351	50,934
400	17,768	4,516	13,252	54,650
400	20,510	8,055	12,455	51,363
400	20,347	6,102	14,245	58,745
400	15,979	7,952	8,027	33,103
400	16,793	9,745	7,048	29,065
400	12,640	7,584	5,056	20,850
400	12,467	7,079	5,388	22,220
400	12,983	6,976	6,007	24,772
400	15,959	7,738	8,221	33,903
400	17,345	7,288	10,057	41,474
400	12,477	8,558	3,919	16,162
400	23,262	11,992	11,270	46,476
400	24,580	11,499	13,081	53,945
400	23,813	11,622	12,191	50,274
400	19,736	11,262	8,474	34,946
400	29,098	15,069	14,029	57,854

Fru	RI	BKG	RI-BKG	RI%
0	30,211	9,508	20,703	85,377
0	43,788	8,866	34,922	144,015
0	30,246	12,723	17,523	72,263
0	40,556	9,824	30,732	126,736
0	39,992	8,340	31,652	130,530
0	32,353	10,294	22,059	90,969
0	32,061	12,021	20,040	82,643
0	29,637	14,066	15,571	64,213
0	51,488	26,450	25,038	103,254
50	56,422	9,568	46,854	193,221
50	38,289	9,542	28,747	118,550
50	23,376	10,921	12,455	51,363
50	24,077	12,260	11,817	48,732
50	40,418	8,204	32,214	132,847
50	15,117	13,038	2,079	8,574
50	24,108	11,791	12,317	50,794
50	20,252	10,164	10,088	41,602
50	19,568	8,880	10,688	44,076
50	16,558	10,107	6,451	26,603
50	38,379	8,529	29,850	123,098
50	21,883	10,760	11,123	45,870
50	18,817	8,212	10,605	43,734
50	22,162	7,729	14,433	59,520

200	34,663	11,364	23,299	91,265
200	29,025	10,249	18,776	73,548
200	33,330	13,812	19,518	76,454
200	33,282	16,794	16,488	64,585
200	37,416	15,640	21,776	85,299
200	39,571	17,796	21,775	85,295
200	50,288	21,418	28,870	113,087
200	30,810	17,993	12,817	50,206
200	44,654	19,056	25,598	100,270
200	34,813	21,130	13,683	53,598
200	48,547	24,482	24,065	94,265
200	37,817	20,702	17,115	67,041
200	46,242	26,556	19,686	77,112
200	41,113	22,802	18,311	71,726
200	32,841	20,619	12,222	47,875
200	36,516	22,033	14,483	56,732
200	47,503	22,472	25,031	98,049
200	44,983	21,392	23,591	92,409
200	39,973	16,566	23,407	91,688
200	35,167	15,452	19,715	77,226
200	41,412	15,443	25,969	101,724
200	42,699	20,805	21,894	85,761
200	48,629	20,113	28,516	111,701
200	45,571	19,390	26,181	102,554
400	29,407	25,571	3,836	15,026
400	20,403	13,317	7,086	27,757
400	23,238	21,242	1,996	7,819
400	17,729	11,904	5,825	22,817
400	28,449	19,921	8,528	33,405
400	16,938	15,880	1,058	4,144
400	36,182	26,107	10,075	39,465
400	34,808	19,960	14,848	58,161
400	20,633	9,876	10,757	42,136
400	30,879	17,344	13,535	53,018
400	31,285	19,967	11,318	44,334
400	42,721	18,377	24,344	95,358
400	33,800	16,902	16,898	66,191
400	30,920	16,660	14,260	55,858
400	24,570	6,579	17,991	70,473
400	23,777	10,342	13,435	52,626
400	37,196	15,315	21,881	85,710
400	19,627	10,465	9,162	35,889
400	25,189	12,817	12,372	48,463
400	18,214	9,284	8,930	34,980
400	17,156	12,925	4,231	16,573
400	27,923	8,775	19,148	75,005
400	20,110	16,851	3,259	12,766
400	24,835	12,552	12,283	48,114
400	28,771	11,791	16,980	66,513
400	27,463	10,294	17,169	67,253
400	27,786	14,640	13,146	51,494
400	25,531	10,502	15,029	58,870
400	29,681	10,665	19,016	74,488
400	21,403	10,649	10,754	42,125
400	26,865	11,788	15,077	59,058
400	12,282	10,327	1,955	7,658
400	27,272	10,708	16,564	64,883
400	22,558	13,742	8,816	34,533
400	27,572	13,799	13,773	53,950
400	29,060	12,656	16,404	64,256
800	8,152	9,224	0,000	0,000
800	16,279	13,839	2,440	9,558
800	16,582	8,957	7,625	29,868
800	16,771	14,898	1,873	7,337
800	14,288	8,108	6,180	24,208
800	16,908	16,047	0,861	3,373
800	14,856	9,524	5,332	20,886
800	17,135	17,359	0,000	0,000
800	6,841	12,817	0,000	0,000
800	17,058	16,043	1,015	3,976
800	11,016	9,625	1,391	5,449

50	0,039	3,494	0	0
50	1,003	3,288	0	0
50	1,495	2,696	0	0
50	1,054	2,93	0	0
50	2,02	3,328	0	0
50	2,408	3,443	0	0
50	0,558	3,714	0	0
100	0,786	2,444	0	0
100	0,677	2,484	0	0
100	0,83	1,363	0	0
100	0,005	2,136	0	0
100	1,003	3,445	0	0
100	0,676	2,1	0	0
100	0,396	1,755	0	0
100	0,589	1,882	0	0
100	1,113	2,501	0	0
100	0,943	2,923	0	0
100	1,074	3,059	0	0
100	0,037	3,04	0	0
100	1,002	1,814	0	0
100	0,998	1,74	0	0
100	0,837	2,916	0	0
100	1,127	1,578	0	0
100	0,931	2,853	0	0
200	0,539	2,126	0	0
200	0,576	2,945	0	0
200	1,17	2,347	0	0
200	0,539	2,807	0	0
200	0,216	2,55	0	0
200	0,993	3,013	0	0
200	1,059	2,806	0	0
200	0,571	3,236	0	0
200	1,041	1,965	0	0
200	0,048	2,308	0	0
200	0,12	1,375	0	0
200	0,6	2,32	0	0
200	0,045	2,377	0	0
400	0	1,323	0	0
400	0,042	1,186	0	0
400	0,003	0,997	0	0
400	0,065	0,679	0	0
400	0	1,073	0	0
400	0,004	1,27	0	0
400	0	0,976	0	0
400	0	1,163	0	0
400	0,082	1,019	0	0
400	0	1,52	0	0
400	0	1,516	0	0
400	0,005	1,021	0	0
400	0	0,985	0	0
400	0	1,019	0	0
400	0,237	0,977	0	0
400	0,022	0,903	0	0
400	0,024	0,898	0	0
400	0,019	0,981	0	0
400	0	1,17	0	0
400	0,003	1,042	0	0

Fru	IM CA	IM BKG	INT-BKG	RI%
0	35,966	6,14	29,826	119,3749
0	28,19	4,32	23,87	95,53678
0	37,483	9,578	27,905	111,6864
0	28,901	2,071	26,83	107,3838
0	31,767	6,088	25,679	102,7771
0	28,923	5,719	23,204	92,87119
0	23,989	6,407	17,582	70,36982
50	2,348	7,197	0	0
50	4,253	5,894	0	0
50	1,723	7,15	0	0
50	2,9	6,09	0	0
50	1,518	3,466	0	0

100	9,797	0,917	8,88	74,12
100	15,178	2,083	13,10	109,31
100	8,924	0,948	7,98	66,58
100	7,564	1,115	6,45	53,83
100	13,869	1,031	12,84	107,16
100	18,304	1,337	16,97	141,63
100	13,7	1,814	11,89	99,22
100	6,913	1,366	5,55	46,30
100	7,984	1,227	6,76	56,40
100	20,484	1,629	18,86	157,39
100	19,573	1,66	17,91	149,53
100	12,987	1,499	11,49	95,89
100	14,276	1,644	12,63	105,44
100	20,379	1,691	18,69	156,00
100	11,459	1,282	10,18	84,95
100	18,755	1,423	17,33	144,68
100	9,86	1,877	7,98	66,64
100	8,53	1,64	6,89	57,51
100	15,698	1,948	13,75	114,78
100	6,334	1,726	4,61	38,46
100	13,817	1,778	12,04	100,49
100	12,622	1,995	10,63	88,71
200	20,466	0,916	19,55	163,19
200	19,906	0,905	19,00	158,61
200	13,007	0,899	12,11	101,07
200	10,082	1,349	8,73	72,90
200	16,833	0,873	15,96	133,22
200	12,558	0,857	11,70	97,67
200	9,779	1,109	8,67	72,37
200	9,101	1,087	8,01	66,90
200	24,574	0,977	23,60	196,97
200	27,702	0,988	26,71	222,99
200	6,052	1,032	5,02	41,90
200	11,891	0,942	10,95	91,40
200	10,936	1,012	9,92	82,84
200	16,099	1,066	15,03	125,49
200	20,517	1,04	19,48	162,58
200	12,136	1,02	11,12	92,79
200	9,141	0,995	8,15	68,00
200	15,522	1,014	14,51	121,10
200	14,559	1,457	13,10	109,37
200	11,848	1,297	10,55	88,07
200	15	0,546	14,45	120,65
200	7,873	0,659	7,21	60,22
200	10,667	0,885	9,78	81,65
200	12,246	0,783	11,46	95,69
200	6,713	0,965	5,75	47,98
200	23,275	1,146	22,13	184,72
200	11,055	1,05	10,01	83,52
200	12,871	1,023	11,85	98,90
200	19,146	0,878	18,27	152,49
200	20,215	1,743	18,47	154,19
200	13,878	1,481	12,40	103,48
200	7,983	1,596	6,39	53,31
200	17,815	1,185	16,63	138,82
200	20,161	1,345	18,82	157,06
200	15,886	1,682	14,20	118,57
200	12,971	1,293	11,68	97,48
200	18,694	1,111	17,58	146,77
200	15,635	1,571	14,06	117,40
200	22,045	1,859	20,19	168,50
200	7,681	1,633	6,05	50,49
200	23,793	0,993	22,80	190,32
200	13,879	3,235	10,64	88,85
200	5,112	1,294	3,82	31,87
200	14,818	1,244	13,57	113,31
200	15,356	0,887	14,47	120,78
200	9,613	0,933	8,68	72,46
200	17,62	1,05	16,57	138,32
200	7,318	0,952	6,37	53,14
200	10,865	1,147	9,72	81,12

0	16,367	1,922	14,445	38,479
0	11,444	3,010	8,434	22,467
0	37,952	2,610	35,342	94,146
0	51,358	2,043	49,315	131,368
0	55,903	2,178	53,725	143,115
0	47,875	2,763	45,112	120,171
0	15,278	2,382	12,896	34,353
50	26,034	2,387	23,647	62,992
50	21,153	3,440	17,713	47,185
50	54,792	3,403	51,389	136,892
50	47,506	2,877	44,629	118,885
50	19,552	2,925	16,627	44,292
50	37,420	2,985	34,435	91,730
50	27,706	2,578	25,128	66,937
50	43,611	2,094	41,517	110,595
50	37,053	2,190	34,863	92,870
50	39,508	2,741	36,767	97,942
50	39,730	2,757	36,973	98,490
50	40,870	2,991	37,879	100,904
50	18,773	2,211	16,562	44,119
50	47,999	3,430	44,569	118,725
50	40,188	2,672	37,516	99,937
50	29,056	2,986	26,070	69,446
50	21,593	2,918	18,675	49,747
50	27,264	2,537	24,727	65,869
50	8,560	2,879	5,681	15,133
50	24,268	2,573	21,695	57,792
50	33,394	2,825	30,569	81,431
50	13,605	2,102	11,503	30,642
50	27,619	2,210	25,409	67,686
50	19,076	2,932	16,144	43,005
50	20,659	2,848	17,811	47,446
50	34,674	2,318	32,356	86,191
50	11,878	2,328	9,550	25,440
50	7,001	2,771	4,230	11,268
50	33,162	3,000	30,162	80,347
50	11,087	2,337	8,750	23,309
100	15,573	15,462	0,111	0,296
100	18,335	2,772	15,563	41,457
100	14,594	2,633	11,961	31,862
100	16,142	2,603	13,539	36,066
100	14,723	2,437	12,286	32,728
100	7,194	3,088	4,106	10,938
100	15,722	3,139	12,583	33,519
100	14,372	2,506	11,866	31,609
100	14,385	3,517	10,868	28,951
100	20,036	3,457	16,579	44,164
100	11,809	2,871	8,938	23,809
100	14,541	3,184	11,357	30,253
100	11,526	3,072	8,454	22,520
100	9,240	3,207	6,033	16,071
200	5,782	1,725	4,057	10,807
200	12,067	1,879	10,188	27,139
200	15,425	1,962	13,463	35,863
200	7,970	1,925	6,045	16,103
200	16,388	1,832	14,556	38,775
200	16,131	2,538	13,593	36,210
200	10,184	1,902	8,282	22,062
200	16,270	2,172	14,098	37,555
200	14,233	2,319	11,914	31,737
200	12,901	1,370	11,531	30,717
200	9,958	1,373	8,585	22,869
200	4,767	1,419	3,348	8,919
200	5,313	1,425	3,888	10,357
400	1,581	1,441	0,140	0,373
400	1,309	1,051	0,258	0,687
400	1,133	2,202	0,000	0,000
400	1,038	1,360	0,000	0,000
400	2,035	1,684	0,351	0,935
400	1,973	1,491	0,482	1,284
400	1,903	1,437	0,466	1,241

100	19,253	0,687	18,57	88,71
100	30,051	1,6	28,45	135,93
100	29,518	3,475	26,04	124,43
100	28,115	2,032	26,08	124,62
100	21,156	2,693	18,46	88,21
100	26,795	1,766	25,03	119,58
100	31,789	2,812	28,98	138,45
100	7,226	2,566	4,66	22,26
100	18,526	2,038	16,49	78,78
100	17,338	2,038	15,30	73,10
100	10,232	3,063	7,17	34,25
200	15,971	2,612	13,36	63,83
200	17,796	2,109	15,69	74,95
200	12,304	1,883	10,42	49,79
200	5,051	1,993	3,06	14,61
200	3,709	1,75	1,96	9,36
200	2,889	1,797	1,09	5,22
200	25,179	2,502	22,68	108,35
200	3,216	2,675	0,54	2,58
200	18,205	3,42	14,79	70,64
200	7,978	1,107	6,87	32,83
200	7,953	1,963	5,99	28,62
200	4,012	1,937	2,08	9,91
200	2,229	1,787	0,44	2,11
200	2,586	3,346	0,00	0,00
200	3,362	2,834	0,53	2,52
200	9,129	2,346	6,78	32,41
400	3,035	1,901	1,13	5,42
400	4,4	1,823	2,58	12,31
400	2,946	2,435	0,51	2,44
400	1,516	3,261	0,00	0,00
400	4,098	2,085	2,01	9,62
400	4,169	2,926	1,24	5,94
400	3,345	2,297	1,05	5,01
400	1,869	3,04	0,00	0,00
400	2,621	2,945	0,00	0,00
400	2,508	2,299	0,21	1,00

Fru	IM CA	IM BKG	IF-BKG	RI%
0	41,403	0,807	40,60	87,51
0	49,78	1,013	48,77	105,12
0	32,299	1,05	31,25	67,36
0	30,702	1,009	29,69	64,01
0	38,534	1,264	37,27	80,34
0	42,174	1,375	40,80	87,95
0	36,423	1,319	35,10	75,67
0	63,978	1,063	62,92	135,62
0	53,389	1,16	52,23	112,59
0	59,37	1,019	58,35	125,78
0	58,97	1,148	57,82	124,64
0	61,226	1,85	59,38	127,99
0	22,948	1,395	21,55	46,46
0	67,519	1,027	66,49	143,33
0	41,905	1,315	40,59	87,50
0	48,942	1,062	47,88	103,21
0	64,425	1,037	63,39	136,64
0	46,933	1,658	45,28	97,60
0	53,454	1,15	52,30	112,75
0	58,032	1,082	56,95	122,76
0	25,781	1,15	24,63	53,10
0	51,06	1,17	49,89	107,54
0	40,013	1,279	38,73	83,50
0	49,64	1,641	48,00	103,47
0	53,212	1,166	52,05	112,19
0	48,332	1,068	47,26	101,88
0	26,393	1,111	25,28	54,50
0	60,692	1,085	59,61	128,49
0	56,318	1,562	54,76	118,03
0	42,968	1,057	41,91	90,34
0	36,374	1,098	35,28	76,04
0	54,248	1,344	52,90	114,04

50	19,124	6,299	12,825	52,889
50	21,186	7,547	13,639	56,246
50	21,456	5,878	15,578	64,242
50	13,518	6,372	7,146	29,469
50	21,614	9,956	11,658	48,076
50	24,532	14,459	10,073	41,540
50	42,827	11,896	30,931	127,556
50	44,041	14,324	29,717	122,550
50	26,426	14,160	12,266	50,584
50	37,943	10,966	26,977	111,250
50	62,805	22,284	40,521	167,105
50	29,553	13,538	16,015	66,044
50	64,031	21,722	42,309	174,478
50	35,423	15,213	20,210	83,344
50	45,802	19,771	26,031	107,349
50	37,030	16,288	20,742	85,538
50	43,877	21,221	22,656	93,431
50	46,822	7,476	39,346	162,259
50	45,091	7,771	37,320	153,904
50	31,081	11,191	19,890	82,024
50	32,491	11,108	21,383	88,181
50	27,484	8,596	18,888	77,892
50	30,569	8,593	21,976	90,627
100	32,112	12,927	19,185	79,117
100	37,806	10,794	27,012	111,395
100	28,125	13,284	14,841	61,203
100	29,696	10,348	19,348	79,789
100	20,132	11,000	9,132	37,659
100	27,823	10,056	17,767	73,269
100	32,716	11,820	20,896	86,173
100	11,959	10,678	1,281	5,283
100	18,860	9,885	8,975	37,012
100	31,285	8,256	23,029	94,969
100	21,615	8,347	13,268	54,716
200	26,719	10,725	15,994	65,958
200	24,080	12,676	11,404	47,029
200	17,395	8,571	8,824	36,389
200	18,967	8,130	10,837	44,691
200	17,175	7,828	9,347	38,546
200	32,174	14,890	17,284	71,277
200	26,211	9,972	16,239	66,968
200	19,188	9,723	9,465	39,033
200	24,882	9,851	15,031	61,986
200	25,984	8,711	17,273	71,232
200	14,637	10,238	4,399	18,141
200	14,247	8,666	5,581	23,015
200	27,344	7,381	19,963	82,325
200	14,535	7,258	7,277	30,010
200	14,099	8,049	6,050	24,950
200	13,985	10,642	3,343	13,786
200	13,470	6,945	6,525	26,908
200	16,281	9,926	6,355	26,207
200	16,053	7,959	8,094	33,379
200	14,910	7,906	7,004	28,884
200	15,284	8,433	6,851	28,253
200	20,775	6,474	14,301	58,976
200	22,745	10,193	12,552	51,763
200	26,221	10,979	15,242	62,856
200	18,106	8,723	9,383	38,695
200	17,323	8,776	8,547	35,247
200	15,411	5,704	9,707	40,031
200	16,886	6,260	10,626	43,821
200	17,576	6,219	11,357	46,835
200	16,422	4,915	11,507	47,454
200	16,335	5,970	10,365	42,744
200	27,139	5,307	21,832	90,033
200	12,782	3,600	9,182	37,866
200	15,315	7,328	7,987	32,938
200	20,346	5,115	15,231	62,811
200	14,205	7,419	6,786	27,985
200	13,650	9,926	3,724	15,357

800	14,193	16,512	0,000	0,000
800	13,465	12,197	1,268	4,967
800	12,957	17,258	0,000	0,000
800	16,457	8,046	8,411	32,947
800	13,690	13,276	0,414	1,622
800	12,000	8,862	3,138	12,292
800	11,040	17,905	0,000	0,000
800	11,712	11,151	0,561	2,198
800	13,759	15,636	0,000	0,000
800	13,989	10,775	3,214	12,590
800	15,420	14,988	0,432	1,692
800	14,502	13,088	1,414	5,539
800	10,887	5,860	5,027	19,691
800	14,431	12,022	2,409	9,436
800	18,995	13,377	5,618	22,006
800	10,771	6,173	4,598	18,011
800	18,914	13,053	5,861	22,958
800	11,878	8,104	3,774	14,783
800	18,330	11,853	6,477	25,371
800	9,588	8,158	1,430	5,601
800	12,372	15,138	0,000	0,000
800	16,586	11,287	5,299	20,757
800	17,284	8,483	8,801	34,475
800	15,319	10,201	5,118	20,048
800	10,963	12,019	0,000	0,000
800	14,044	10,264	3,780	14,807
800	9,198	9,687	0,000	0,000
800	11,970	6,269	5,701	22,331
800	16,469	9,415	7,054	27,631
800	19,643	9,140	10,503	41,141
800	14,088	9,925	4,163	16,307
800	16,366	8,964	7,402	28,995
800	16,855	10,146	6,709	26,280
800	14,803	7,648	7,155	28,027
800	11,586	9,814	1,772	6,941
800	7,366	5,121	2,245	8,794
800	16,962	12,010	4,952	19,398
800	13,563	8,525	5,038	19,734
800	17,335	10,916	6,419	25,144
800	13,121	8,995	4,126	16,162
800	13,962	9,837	4,125	16,158
800	14,326	8,915	5,411	21,196
800	17,438	12,916	4,522	17,713
800	15,535	12,361	3,174	12,433
800	11,850	11,320	0,530	2,076
800	14,655	10,216	4,439	17,388
800	11,541	8,968	2,573	10,079

Fru	RI	BKG	RI-BKG	RI%
0	13,635	4,847	8,788	88,832
0	13,540	3,972	9,568	96,716
0	12,152	5,012	7,140	72,173
0	15,908	3,174	12,734	128,719
0	13,621	3,785	9,836	99,426
0	13,104	4,869	8,235	83,242
0	14,494	3,146	11,348	114,709
0	17,895	4,573	13,322	134,663
0	10,100	4,257	5,843	59,063
0	14,919	5,991	8,928	90,247
0	12,472	4,844	7,628	77,106
0	18,707	6,686	12,021	121,512
0	16,489	5,096	11,393	115,164
0	10,306	5,144	5,162	52,179
0	11,981	6,363	5,618	56,789
0	13,971	4,940	9,031	91,288
0	14,290	2,565	11,725	118,520
0	16,373	4,347	12,026	121,563
0	15,072	2,527	12,545	126,809
0	19,822	6,274	13,548	136,948
0	17,784	5,448	12,336	124,696
0	14,086	5,328	8,758	88,529

100	0,855	1,935	0	0
100	0,579	1,963	0	0
100	0,833	3,014	0	0
100	1	3,72	0	0
100	1	3,85	0	0
200	0,006	2,925	0	0
200	0	1,452	0	0
200	0	1,3	0	0
200	0,216	2,395	0	0
200	0,367	3,12	0	0
200	0,088	1,615	0	0
400	1	1,868	0	0
400	0,803	1,045	0	0
400	0,221	1,567	0	0
400	0,176	1,926	0	0
400	0,271	1,584	0	0
400	0,242	0,819	0	0
400	0,422	1,574	0	0
400	0,11	1,97	0	0
400	0,946	1,169	0	0
400	1,024	2,386	0	0

Gal	IM CA	IM BKG	INT-BKG	RI%
0	18,9	6,301	12,599	61,02097
0	24,977	7,978	16,999	82,33157
0	23,415	7,177	16,238	78,64581
0	23,767	5,255	18,512	89,65951
0	29,3	3,87	25,43	123,1656
0	27,653	5,517	22,136	107,2117
0	28,779	4,586	24,193	117,1744
0	29,748	5,912	23,836	115,4453
0	29,56	3,763	25,797	124,9431
0	31,519	5,25	26,269	127,2291
0	26,651	6,252	20,399	98,79886
0	26,074	5,42	20,654	100,0339
0	18,899	4,663	14,236	68,94948
0	28,69	5,339	23,351	113,0963
0	27,249	6,693	20,556	99,55926
0	27,302	7,085	20,217	97,91737
0	35,133	5,629	29,504	142,8973
0	25,815	5,153	20,662	100,0726
0	23,88	4,765	19,115	92,58004
0	17,367	5,13	12,237	59,26769
50	36,239	7,669	28,57	138,3736
50	37,163	7,002	30,161	146,0793
50	23,629	7,7	15,929	77,14922
50	24,452	8,054	16,398	79,42074
50	33,69	9,94	23,75	115,0288
50	33,684	9,77	23,914	115,8231
50	35,354	5,364	29,99	145,2511
50	38,582	5,79	32,792	158,8221
50	38,402	5,893	32,509	157,4514
50	35,581	7,748	27,833	134,8041
50	38,132	6,363	31,769	153,8674
50	47,536	6,259	41,277	199,9177
50	48,791	9,06	39,731	192,4299
50	42,551	6,431	36,12	174,9407
50	41,618	7,202	34,416	166,6877
50	27,857	5,644	22,213	107,5846
50	23,078	8,003	15,075	73,01303
50	45,997	7,78	38,217	185,0971
50	30,914	5,273	25,641	124,1875
50	15,431	5,164	10,267	49,72635
50	20,447	4,562	15,885	76,93612
50	20,781	3,467	17,314	83,85722
50	19,518	5,545	13,973	67,67569
50	18,101	6,774	11,327	54,86027
50	20,764	5,933	14,831	71,83126
50	15,237	4,324	10,913	52,85514
50	26,449	6,124	20,325	98,44045
50	28,552	5,73	22,822	110,5342

400	7,09	0,7	6,39	53,34
400	5,861	0,848	5,01	41,85
400	10,219	0,995	9,22	77,00
400	14,427	1,022	13,41	111,90
400	5,398	1,002	4,40	36,70
400	16,804	0,608	16,20	135,19
400	14,741	1,001	13,74	114,69
400	29,229	0,886	28,34	236,59
400	11,409	0,837	10,57	88,25
400	6,854	0,867	5,99	49,98
400	12,438	0,857	11,58	96,67
400	6,849	0,75	6,10	50,91
400	24,131	0,998	23,13	193,10
400	16,804	0,77	16,03	133,84
400	15,141	0,867	14,27	119,15
400	24,964	0,653	24,31	202,93
400	16,043	0,894	15,15	126,45
400	14,607	0,941	13,67	114,08
400	9,35	0,948	8,40	70,13
400	8,14	1,152	6,99	58,33
400	9,603	1,067	8,54	71,25
400	16,854	1,028	15,83	132,11
400	16,151	0,968	15,18	126,74
400	16,872	0,982	15,89	132,64
400	14,615	1,058	13,56	113,17
400	16,739	0,851	15,89	132,62
400	27,283	0,975	26,31	219,60
400	24,766	0,971	23,80	198,63
400	12,147	0,801	11,35	94,71
400	27,819	0,613	27,21	227,10
400	12,466	0,857	11,61	96,90
400	29,176	0,743	28,43	237,34
400	24,464	1,018	23,45	195,71
400	23,611	0,772	22,84	190,65
400	12,228	0,711	11,52	96,14
400	21,151	0,974	20,18	168,43
400	7,332	0,833	6,50	54,25
400	5,764	0,617	5,15	42,96
400	10,999	0,824	10,18	84,93
400	12,342	0,544	11,80	98,48
400	7,365	0,71	6,66	55,55
400	5,254	0,898	4,36	36,36
400	5,492	0,653	4,84	40,39
400	6,615	0,778	5,84	48,72
400	17,233	0,594	16,64	138,89
400	7,261	0,448	6,81	56,87
800	5,864	0,89	4,97	41,52
800	9,318	1,014	8,30	69,32
800	6,501	0,937	5,56	46,44
800	13,959	1,007	12,95	108,12
800	9,513	0,94	8,57	71,56
800	4,016	0,782	3,23	27,00
800	6,854	0,634	6,22	51,92
800	6,242	0,946	5,30	44,21
800	6	1	5,00	41,74
800	11,084	0,803	10,28	85,82
800	10,724	0,535	10,19	85,05
800	11,043	0,816	10,23	85,37
800	9,009	0,797	8,21	68,55
800	13,588	1,115	12,47	104,12
800	17,252	0,748	16,50	137,77
800	24,569	0,865	23,70	197,87
800	13,382	0,925	12,46	103,98
800	18,239	0,918	17,32	144,59
800	11,082	0,915	10,17	84,87
800	12,462	1,809	10,65	88,92
800	13,923	0,965	12,96	108,17
800	20,133	0,704	19,43	162,18
800	10,768	0,868	9,90	82,64
800	27,767	1,254	26,51	221,31
800	28,426	1,117	27,31	227,96

Fru	IM CA	IM BKG	IM-BKG	RI%
0	27,530	1,611	25,919	83,135
0	48,973	2,793	46,180	148,122
0	36,648	3,151	33,497	107,441
0	41,238	3,978	37,260	119,511
0	42,574	2,819	39,755	127,514
0	38,813	2,718	36,095	115,774
0	31,945	3,170	28,775	92,295
0	34,684	3,195	31,489	101,001
0	38,382	3,325	35,057	112,445
0	40,198	3,914	36,284	116,380
0	52,811	3,940	48,871	156,753
0	47,066	2,905	44,161	141,646
0	7,532	4,019	3,513	11,268
0	11,173	4,005	7,168	22,991
0	27,635	4,080	23,555	75,552
0	46,070	5,284	40,786	130,821
0	34,659	2,953	31,706	101,697
0	20,124	2,145	17,979	57,667
0	26,636	2,322	24,314	77,987
50	23,899	0,923	22,976	73,695
50	23,982	1,154	22,828	73,221
50	21,297	1,142	20,155	64,647
50	24,352	0,884	23,468	75,273
50	14,035	1,035	13,000	41,697
50	52,698	1,272	51,426	164,948
50	18,085	1,660	16,425	52,683
100	21,159	1,036	20,123	64,544
100	27,782	0,965	26,817	86,015
100	22,342	1,036	21,306	68,339
100	28,181	1,208	26,973	86,516
100	19,526	1,167	18,359	58,886
100	17,400	0,802	16,598	53,238
200	11,353	1,137	10,216	32,768
200	16,725	1,252	15,473	49,629
200	15,785	1,260	14,525	46,589
200	7,665	1,205	6,460	20,720
200	12,267	1,034	11,233	36,030
400	2,780	1,743	1,037	3,326
400	2,854	1,554	1,300	4,170
400	3,906	1,335	2,571	8,246
400	4,938	1,989	2,949	9,459
400	2,147	1,771	0,376	1,206
400	3,622	1,306	2,316	7,429
400	2,667	1,980	0,687	2,204

Gal	IM CA	IM BKG	IM-BKG	RI%
0	27,530	1,611	25,919	83,135
0	48,973	2,793	46,180	148,122
0	36,648	3,151	33,497	107,441
0	41,238	3,978	37,260	119,511
0	42,574	2,819	39,755	127,514
0	38,813	2,718	36,095	115,774
0	31,945	3,170	28,775	92,295
0	34,684	3,195	31,489	101,001
0	38,382	3,325	35,057	112,445
0	40,198	3,914	36,284	116,380
0	52,811	3,940	48,871	156,753
0	47,066	2,905	44,161	141,646
0	7,532	4,019	3,513	11,268
0	11,173	4,005	7,168	22,991
0	27,635	4,080	23,555	75,552
0	46,070	5,284	40,786	130,821
0	34,659	2,953	31,706	101,697
0	20,124	2,145	17,979	57,667
0	26,636	2,322	24,314	77,987
50	19,757	1,198	18,559	59,528
50	14,652	1,699	12,953	41,547
50	22,071	2,278	19,793	63,486
50	20,658	2,950	17,708	56,798

0	40,751	1,148	39,60	85,37
0	51,05	1,11	49,94	107,65
0	41,06	1,342	39,72	85,62
0	21,669	1,023	20,65	44,51
0	31,839	1,004	30,84	66,47
0	43,552	2,123	41,43	89,31
0	44,039	1,03	43,01	92,71
0	38,387	1,178	37,21	80,21
0	28,168	1,174	26,99	58,19
0	31,92	1,047	30,87	66,55
0	35,317	1,231	34,09	73,48
0	57,423	1,019	56,40	121,59
0	47,749	1,035	46,71	100,70
0	65,686	1,491	64,20	138,38
0	26,187	1	25,19	54,29
0	31,42	0,995	30,43	65,58
0	36,989	1,653	35,34	76,17
0	44,031	1,444	42,59	91,80
0	68,65	1,433	67,22	144,89
0	57,759	1,716	56,04	120,81
0	48,323	1,983	46,34	99,89
0	46,893	2,587	44,31	95,51
0	71,171	2,079	69,09	148,94
0	62,083	2,266	59,82	128,94
0	49,446	2,093	47,35	102,08
0	59,679	1,483	58,20	125,45
0	49,238	2,201	47,04	101,39
0	57,101	2,157	54,94	118,44
0	59,581	1,873	57,71	124,40
0	27,34	1,568	25,77	55,55
0	54,808	1,957	52,85	113,93
0	38,599	1,822	36,78	79,28
0	56,859	1,11	55,75	120,17
0	74,336	4,894	69,44	149,69
0	53,369	1,547	51,82	111,71
0	52,894	1,907	50,99	109,91
0	33,482	1,674	31,81	68,57
0	70,702	2,45	68,25	147,13
0	61,903	1,371	60,53	130,48
0	55,466	1,278	54,19	116,81
0	57,421	1,791	55,63	119,92
0	68,262	1,952	66,31	142,94
0	33,501	2,495	31,01	66,84
0	27,896	1,5	26,40	56,90
0	52,648	1,082	51,57	111,16
0	31,399	1,497	29,90	64,46
0	64,66	0,968	63,69	137,30
50	19,994	0,791	19,20	41,39
50	16,966	1,679	15,29	32,95
50	14,835	1,486	13,35	28,78
50	24,819	0,488	24,33	52,45
50	26,468	1,183	25,29	54,51
50	11,877	2,074	9,80	21,13
50	20,838	1,672	19,17	41,31
50	34,813	1,512	33,30	71,78
50	28,659	1,638	27,02	58,25
50	14,843	1,82	13,02	28,07
50	13,205	2,948	10,26	22,11
50	19,901	2,871	17,03	36,71
50	12,116	3,134	8,98	19,36
50	6,842	2,519	4,32	9,32
50	6,577	2,273	4,30	9,28
50	20,687	1,536	19,15	41,28
50	36,851	2,344	34,51	74,38
50	24,894	2,899	22,00	47,41
50	42,254	1,708	40,55	87,40
50	41,349	1,276	40,07	86,38
50	12,762	1,951	10,81	23,30
50	30,238	2,715	27,52	59,33
50	46,698	3,731	42,97	92,62
50	53,813	2,367	51,45	110,90

200	15,791	8,505	7,286	30,047
200	14,742	8,455	6,287	25,927
200	16,157	5,935	10,222	42,155
200	16,648	6,608	10,040	41,404
200	18,047	7,850	10,197	42,051
200	16,718	6,532	10,186	42,006
200	15,032	7,974	7,058	29,106
200	12,565	8,370	4,195	17,300
200	21,226	8,114	13,112	54,073
200	14,262	6,410	7,852	32,381
200	12,121	5,714	6,407	26,422
200	17,863	7,079	10,784	44,472
200	32,225	12,049	20,176	83,204
200	25,904	12,660	13,244	54,617
200	10,785	6,956	3,829	15,790
200	13,180	6,769	6,411	26,438
200	27,447	8,118	19,329	79,711
200	21,899	6,347	15,552	64,135
200	17,760	6,926	10,834	44,678
200	28,105	15,286	12,819	52,864
200	16,444	7,838	8,606	35,490
200	23,746	9,153	14,593	60,180
200	26,708	9,454	17,254	71,154
400	18,497	11,673	6,824	28,141
400	17,570	12,495	5,075	20,929
400	12,710	9,885	2,825	11,650
400	27,870	15,487	12,383	51,066
400	21,273	12,286	8,987	37,061
400	15,647	10,762	4,885	20,145
400	25,142	13,102	12,040	49,652
400	31,623	13,890	17,733	73,129
400	31,821	13,620	18,201	75,059
400	35,183	16,551	18,632	76,837
400	20,490	11,961	8,529	35,173
400	19,422	6,469	12,953	53,417
400	22,201	12,440	9,761	40,253
400	23,297	12,799	10,498	43,293
400	14,790	13,884	0,906	3,736
400	14,822	12,173	2,649	10,924
400	14,778	12,409	2,369	9,770
400	17,300	12,814	4,486	18,500
400	20,279	12,901	7,378	30,426
400	15,288	9,145	6,143	25,333

Glc	RI	BKG	RI-BKG	RI%
0	12,001	4,397	7,604	128,450
0	14,977	3,007	11,970	202,202
0	13,404	2,842	10,562	178,417
0	9,149	3,229	5,920	100,003
0	11,472	4,955	6,517	110,088
0	11,219	4,022	7,197	121,574
0	7,439	6,311	1,128	19,055
0	13,475	4,436	9,039	152,690
0	11,883	4,256	7,627	128,838
0	10,272	4,445	5,827	98,432
0	11,599	3,638	7,961	134,480
0	11,272	2,822	8,450	142,741
0	7,599	3,401	4,198	70,914
0	9,404	3,600	5,804	98,043
0	11,910	4,217	7,693	129,953
0	19,761	5,489	14,272	241,088
0	17,384	4,551	12,833	216,780
0	16,511	6,115	10,396	175,613
0	11,892	4,407	7,485	126,439
0	5,816	3,465	2,351	39,714
0	13,606	4,018	9,588	161,964
0	7,697	2,745	4,952	83,651
0	5,439	3,267	2,172	36,690
0	4,918	3,228	1,690	28,548
0	4,075	2,826	1,249	21,099
0	4,477	2,750	1,727	29,173

0	14,845	6,141	8,704	87,983
0	13,838	5,327	8,511	86,032
0	11,697	5,640	6,057	61,226
0	9,936	5,060	4,876	49,288
0	20,276	6,293	13,983	141,345
0	15,358	5,557	9,801	99,072
0	17,706	5,912	11,794	119,218
0	22,777	7,551	15,226	153,909
0	16,654	7,974	8,680	87,740
0	21,679	5,613	16,066	162,400
0	15,776	6,052	9,724	98,293
0	23,856	5,525	18,331	185,296
0	10,614	4,743	5,871	59,346
0	11,272	4,358	6,914	69,889
0	11,046	3,808	7,238	73,164
0	12,074	6,642	5,432	54,908
0	16,378	6,890	9,488	95,908
0	15,753	6,785	8,968	90,651
0	17,544	7,013	10,531	106,451
0	18,368	6,597	11,771	118,985
200	14,830	5,303	9,527	96,302
200	12,376	6,017	6,359	64,279
200	15,113	6,929	8,184	82,727
200	12,621	6,300	6,321	63,895
200	12,930	9,491	3,439	34,763
200	11,493	6,076	5,417	54,757
200	12,299	7,837	4,462	45,103
200	15,637	7,771	7,866	79,512
200	11,807	6,051	5,756	58,184
200	12,902	6,582	6,320	63,885
200	15,872	5,560	10,312	104,237
200	18,638	6,419	12,219	123,514
200	19,101	7,035	12,066	121,967
200	13,097	8,228	4,869	49,217
200	8,893	6,471	2,422	24,482
200	8,681	4,977	3,704	37,441
200	10,401	7,515	2,886	29,173
200	11,346	4,224	7,122	71,992
200	10,995	5,826	5,169	52,250
200	9,159	5,099	4,060	41,040
200	9,884	5,336	4,548	45,973
200	7,759	6,264	1,495	15,112
200	8,598	4,001	4,597	46,468
200	11,578	4,980	6,598	66,695
200	11,483	7,239	4,244	42,900
200	16,536	7,759	8,777	88,721
200	14,724	6,643	8,081	81,685
200	14,923	6,866	8,057	81,443
200	14,059	7,383	6,676	67,483
200	13,225	5,698	7,527	76,085
400	10,887	5,305	5,582	56,425
400	11,488	5,785	5,703	57,648
400	10,304	6,244	4,060	41,040
400	12,831	6,249	6,582	66,533
400	11,752	5,926	5,826	58,891
400	13,772	6,134	7,638	77,207
400	11,732	5,728	6,004	60,690
400	12,996	4,374	8,622	87,154
400	14,442	5,535	8,907	90,035
400	13,260	5,546	7,714	77,976
400	12,804	5,739	7,065	71,415
400	13,771	5,517	8,254	83,434
400	12,898	7,848	5,050	51,047
400	10,131	7,028	3,103	31,366
400	10,823	5,947	4,876	49,288
400	10,398	4,703	5,695	57,567
400	11,539	5,203	6,336	64,046
400	13,015	6,819	6,196	62,631
400	12,366	5,674	6,692	67,645
400	9,971	6,182	3,789	38,300
400	7,606	3,929	3,677	37,168

50	25,905	5,383	20,522	99,39459
50	27,23	8,067	19,163	92,81252
50	23,546	7,229	16,317	79,02843
50	28,453	8,257	20,196	97,81566
50	22,189	6,693	15,496	75,05207
100	40,579	8,947	31,632	153,2039
100	37,912	6,542	31,37	151,9349
100	19,359	8,339	11,02	53,37337
100	31,063	5,868	25,195	122,0274
100	35,706	11,086	24,62	119,2425
100	27,549	9,183	18,366	88,95239
100	28,443	21,004	7,439	36,02945
100	36,784	7,294	29,49	142,8295
100	24,127	7,798	16,329	79,08655
100	30,981	20,147	10,834	52,47251
100	46,422	4,111	42,311	204,9257
100	39,82	5,268	34,552	167,3463
200	41,755	6,169	35,586	172,3543
200	37,623	6,709	30,914	149,7264
200	26,177	6,942	19,235	93,16123
200	41,642	5,959	35,683	172,8241
200	36,005	5,854	30,151	146,0309
200	33,452	4,42	29,032	140,6112
200	24,776	6,949	17,827	86,34184
200	29,685	5,663	24,022	116,3462
200	28,938	6,149	22,789	110,3744
200	18,434	6,558	11,876	57,51925
400	30,234	5,538	24,696	119,6106
400	21,56	6,298	15,262	73,91873
400	24,659	6,597	18,062	87,48002
400	28,617	6,55	22,067	106,8775
400	24,227	7,277	16,95	82,09425
400	23,187	6,444	16,743	81,09168
400	27,484	6,545	20,939	101,4142
400	28,268	6,199	22,069	106,8872
400	23,797	6,937	16,86	81,65835
400	30,539	8,47	22,069	106,8872
400	29,597	5,916	23,681	114,6946
400	26,716	5,121	21,595	104,5915
400	23,44	5,281	18,159	87,94982
400	20,936	5,706	15,23	73,76374
400	16,837	5,169	11,668	56,51184
400	18,143	5,569	12,574	60,89989
400	17,117	5,6	11,517	55,7805
400	12,819	4,777	8,042	38,94997

NAcGlc	IM CA	IM BKG	INT-BKG	RI%
0	35,791	11,034	24,757	83,50592
0	48,24	10,097	38,143	128,6572
0	36,411	8,635	27,776	93,68907
0	40,438	14,485	25,953	87,54005
0	42,122	9,029	33,093	111,6234
0	36,537	8,553	27,984	94,39066
0	42,146	11,385	30,761	103,7575
0	40,506	11,797	28,709	96,8361
50	2,67	5,387	0	0
50	2,286	5,321	0	0
50	2,209	6,621	0	0
50	2,716	6,916	0	0
50	2,819	5,197	0	0
50	2,242	6,333	0	0
50	2,311	6,746	0	0
50	2,021	4,494	0	0
50	1,669	5,569	0	0
100	2,029	5,076	0	0
100	2,499	7,92	0	0
100	3,266	9,024	0	0
100	2,677	9,688	0	0
100	2,75	5,868	0	0
100	3,185	7,518	0	0
100	3,326	8,348	0	0

800	13,523	1,097	12,43	103,72
800	21,087	1,009	20,08	167,60
800	14,366	1,09	13,28	110,82
800	24,996	1,063	23,93	199,78
800	10,799	1,229	9,57	79,88
800	21,428	1,011	20,42	170,43
800	7,571	1,003	6,57	54,83
800	25,836	1,119	24,72	206,32
800	17,215	1,115	16,10	134,39
800	13,495	1,122	12,37	103,28
800	20,037	1,035	19,00	158,62
800	16,062	1,145	14,92	124,52
800	24,25	0,974	23,28	194,29
800	20,841	0,726	20,12	167,91
800	14,665	1,296	13,37	111,60
800	8,079	1,266	6,81	56,87
800	20,529	1,005	19,52	162,97
800	15,963	0,902	15,06	125,72
800	8,374	1,191	7,18	59,96
800	9,982	1,331	8,65	72,21
800	7,649	1,093	6,56	54,73
800	10,459	0,999	9,46	78,97

Gal	IM CA	IM BKG	CA-BKG	RI%
0	9,074	2,143	6,93	39,65
0	15,992	1,051	14,94	85,48
0	6,965	1,208	5,76	32,94
0	15,654	1,654	14,00	80,10
0	21,842	1,33	20,51	117,35
0	8,599	1,866	6,73	38,52
0	5,943	0,991	4,95	28,33
0	9,214	1,412	7,80	44,64
0	15,417	1,401	14,02	80,19
0	17,258	1,554	15,70	89,85
0	14,105	1,376	12,73	72,83
0	25,585	1,294	24,29	138,97
0	11,421	1,521	9,90	56,64
0	18,426	1,478	16,95	96,96
0	15,141	1,238	13,90	79,54
0	25,558	1,488	24,07	137,71
0	5,917	1,707	4,21	24,09
0	7,58	1,748	5,83	33,37
0	38,069	1,215	36,85	210,85
0	28,066	1,516	26,55	151,90
0	9,157	1,789	7,37	42,15
0	24,691	1,695	23,00	131,57
0	16,985	1,508	15,48	88,55
0	36,283	1,212	35,07	200,65
0	14,347	1,225	13,12	75,07
0	29,562	1,531	28,03	160,37
0	9,573	1,63	7,94	45,44
0	12,876	1,417	11,46	65,56
0	17,351	1,693	15,66	89,58
0	39,859	1,454	38,41	219,72
0	8,039	1,66	6,38	36,50
0	8,007	1,232	6,78	38,76
0	39,109	1,315	37,79	216,23
0	9,191	0,819	8,37	47,90
0	46,19	1,216	44,97	257,31
0	10,28	1,658	8,62	49,33
0	6,731	1,094	5,64	32,25
0	5,998	1,189	4,81	27,51
0	16,297	1,768	14,53	83,12
0	34,425	1,094	33,33	190,69
0	34,921	1,327	33,59	192,20
0	19,075	1,537	17,54	100,34
0	13,743	1,223	12,52	71,63
0	20,495	1,177	19,32	110,52
0	26,845	1,64	25,21	144,20
0	27,486	1,017	26,47	151,44
0	14,928	1,234	13,69	78,35

50	25,250	2,004	23,246	74,561
50	28,903	2,106	26,797	85,951
50	25,923	1,722	24,201	77,624
50	23,332	1,445	21,887	70,202
100	12,775	1,343	11,432	36,668
100	12,303	1,327	10,976	35,205
100	19,098	1,455	17,643	56,590
100	14,821	1,919	12,902	41,383
100	19,221	2,328	16,893	54,184
100	19,905	2,014	17,891	57,385
100	16,221	2,064	14,157	45,408
100	11,967	2,643	9,324	29,907
100	16,110	2,410	13,700	43,943
100	17,986	1,858	16,128	51,730
100	16,909	1,582	15,327	49,161
100	10,138	1,318	8,820	28,290
200	0,832	1,120	0,000	0,000
200	1,030	1,287	0,000	0,000
200	1,288	1,089	0,199	0,638
200	0,669	1,259	0,000	0,000
200	1,074	1,721	0,000	0,000
200	0,925	1,374	0,000	0,000
200	0,878	1,117	0,000	0,000
200	1,038	1,477	0,000	0,000
200	0,454	1,241	0,000	0,000
200	0,874	1,268	0,000	0,000
200	1,495	1,193	0,302	0,969
200	1,139	1,841	0,000	0,000
200	2,998	1,068	1,930	6,190
400	1,734	2,511	0,000	0,000
400	1,949	2,188	0,000	0,000
400	1,379	0,929	0,450	1,443
400	3,034	2,118	0,916	2,938
400	1,600	1,727	0,000	0,000
400	1,971	2,060	0,000	0,000
400	1,644	3,084	0,000	0,000
400	2,266	3,341	0,000	0,000
400	1,694	1,284	0,410	1,315
400	1,526	1,376	0,150	0,481
400	1,227	1,442	0,000	0,000
400	1,000	1,174	0,000	0,000
400	1,725	0,482	1,243	3,987

50	18,336	3,107	15,23	32,83
50	33,708	2,312	31,40	67,68
50	31,799	2,266	29,53	63,66
100	16,97	5,737	11,23	24,21
100	13,699	5,882	7,82	16,85
100	17,592	4,764	12,83	27,65
100	8,348	5,165	3,18	6,86
100	24,249	3,149	21,10	45,48
100	18,813	1,95	16,86	36,35
100	24,43	2,394	22,04	47,50
100	22,04	2,039	20,00	43,11
100	12,61	2,4	10,21	22,01
100	10,307	3,139	7,17	15,45
100	7,658	3,222	4,44	9,56
100	13,571	3,956	9,62	20,73
100	13,176	3,372	9,80	21,13
100	22,912	3,37	19,54	42,13
100	8,946	3,573	5,37	11,58
100	10,606	3,314	7,29	15,72
100	16,676	2,578	14,10	30,39
100	21,279	3,293	17,99	38,77
100	25,653	3,794	21,86	47,12
100	31,322	2,142	29,18	62,90
100	10,778	3,768	7,01	15,11
100	38,689	3,5	35,19	75,85
100	38,197	2,837	35,36	76,22
100	35,577	2,747	32,83	70,77
100	15,885	2,547	13,34	28,75
100	36,45	2,668	33,78	72,82
100	18,429	2,686	15,74	33,94
100	15,163	2,562	12,60	27,16
100	19,417	2,055	17,36	37,43
100	45,826	3,509	42,32	91,22
100	32,356	3,826	28,53	61,50
100	25,281	1,985	23,30	50,22
100	8,268	1,078	7,19	15,50
100	11,897	3,247	8,65	18,65
200	21,439	2,222	19,22	41,42
200	10,304	2,169	8,14	17,54
200	17,729	1,936	15,79	34,04
200	10,413	2,846	7,57	16,31
200	20,24	2,24	18,00	38,80
200	12,629	2,812	9,82	21,16
200	12,094	1,844	10,25	22,10
200	15,266	3,524	11,74	25,31
200	13,489	3,135	10,35	22,32
200	19,561	2,685	16,88	36,38
200	17,789	4,397	13,39	28,87
200	9,35	4,136	5,21	11,24
200	15,132	1,01	14,12	30,44
200	8,886	3,093	5,79	12,49
200	9,5	2,003	7,50	16,16
200	14,111	2,993	11,12	23,97
200	21,797	0,628	21,17	45,63
200	18,937	2,549	16,39	35,33
200	22,66	2,655	20,01	43,12
200	14,789	2,129	12,66	27,29
200	13,714	1,528	12,19	26,27
200	25,504	2,164	23,34	50,31
200	25,915	4,48	21,44	46,21
200	8,462	2,408	6,05	13,05
200	18,962	0,912	18,05	38,91
200	26,61	3,647	22,96	49,50
200	28,719	3,532	25,19	54,29
200	13,123	4,28	8,84	19,06
200	12,855	4,593	8,26	17,81
200	6,811	2,092	4,72	10,17
200	5,763	1,962	3,80	8,19
400	2,241	4,737	0,00	0,00
400	2,574	4,036	0,00	0,00
400	2,422	3,967	0,00	0,00

0	4,993	2,659	2,334	39,427
0	7,370	3,412	3,958	66,860
0	6,597	4,004	2,593	43,802
0	9,215	3,693	5,522	93,280
0	13,530	4,658	8,872	149,869
0	10,342	4,099	6,243	105,459
0	8,511	3,153	5,358	90,509
0	10,214	3,549	6,665	112,588
0	7,646	3,173	4,473	75,560
0	16,707	3,135	13,572	229,263
0	14,734	10,453	4,281	72,316
0	16,876	5,440	11,436	193,181
0	12,109	3,587	8,522	143,957
0	8,672	4,423	4,249	71,776
0	8,475	4,081	4,394	74,225
0	6,461	4,773	1,688	28,514
0	6,097	4,506	1,591	26,876
0	9,965	5,307	4,658	78,685
0	6,705	4,760	1,945	32,856
0	7,373	4,383	2,990	50,508
0	9,084	5,840	3,244	54,799
0	9,037	6,389	2,648	44,731
0	21,924	9,830	12,094	204,296
0	9,093	5,126	3,967	67,012
0	8,623	4,563	4,060	68,583
0	8,054	4,422	3,632	61,353
0	7,123	4,573	2,550	43,076
50	4,857	8,226	0,000	0,000
50	5,107	14,844	0,000	0,000
50	4,156	8,995	0,000	0,000
50	7,200	9,450	0,000	0,000
50	2,944	6,297	0,000	0,000
50	1,310	4,988	0,000	0,000
50	5,223	7,380	0,000	0,000
50	4,055	4,714	0,000	0,000
50	5,279	5,001	0,278	4,696
50	5,749	7,555	0,000	0,000
50	2,452	5,128	0,000	0,000
50	6,435	8,596	0,000	0,000
50	9,314	10,246	0,000	0,000
50	7,981	11,101	0,000	0,000
50	14,403	20,239	0,000	0,000
50	8,168	13,350	0,000	0,000
50	5,764	13,138	0,000	0,000
50	5,317	7,359	0,000	0,000
100	3,011	6,291	0,000	0,000
100	3,509	6,014	0,000	0,000
100	2,800	6,724	0,000	0,000
100	4,077	12,095	0,000	0,000
100	6,162	8,602	0,000	0,000
100	2,357	7,405	0,000	0,000
100	5,270	13,082	0,000	0,000
100	2,622	8,295	0,000	0,000
100	2,493	9,018	0,000	0,000
100	2,221	5,576	0,000	0,000
100	2,667	6,043	0,000	0,000
100	2,357	5,957	0,000	0,000
100	2,763	5,260	0,000	0,000
100	1,549	5,281	0,000	0,000
100	2,934	5,556	0,000	0,000
100	4,359	8,791	0,000	0,000
100	4,724	8,378	0,000	0,000
100	4,076	5,766	0,000	0,000
100	4,251	9,909	0,000	0,000
100	4,962	13,301	0,000	0,000
100	4,046	10,835	0,000	0,000
100	3,399	9,866	0,000	0,000
100	3,614	7,872	0,000	0,000
100	4,250	14,106	0,000	0,000
100	3,776	14,282	0,000	0,000
100	4,586	8,468	0,000	0,000

400	10,614	5,680	4,934	49,874
400	14,304	6,143	8,161	82,494
400	16,025	6,692	9,333	94,341
400	14,434	8,208	6,226	62,934
400	19,362	9,694	9,668	97,727
400	13,243	8,644	4,599	46,488
400	12,305	7,412	4,893	49,460
400	14,146	9,010	5,136	51,916
800	14,086	13,320	0,766	7,743
800	11,900	13,126	0,000	0,000
800	13,381	12,885	0,496	5,014
800	9,558	14,917	0,000	0,000
800	11,208	11,620	0,000	0,000
800	8,327	10,821	0,000	0,000
800	12,749	8,960	3,789	38,300
800	11,390	13,923	0,000	0,000
800	13,002	12,385	0,617	6,237
800	16,131	11,943	4,188	42,334
800	15,072	12,341	2,731	27,606
800	12,059	17,424	0,000	0,000
800	14,185	15,136	0,000	0,000
800	16,558	12,142	4,416	44,638
800	15,373	15,605	0,000	0,000
800	15,039	12,401	2,638	26,666
800	12,554	7,855	4,699	47,499
800	10,705	5,253	5,452	55,111
800	10,496	6,734	3,762	38,028
800	13,329	10,992	2,337	23,623
800	16,913	14,100	2,813	28,435
800	9,960	12,100	0,000	0,000
800	11,059	5,888	5,171	52,270

Glc	RI	BKG	RI-BKG	RI%
0	37,085	11,212	25,873	152,080
0	40,619	17,761	22,858	134,358
0	24,342	12,682	11,660	68,537
0	28,129	13,010	15,119	88,869
0	32,940	17,394	15,546	91,379
0	39,134	15,251	23,883	140,383
0	39,859	10,508	29,351	172,524
0	48,173	15,260	32,913	193,461
0	43,781	13,878	29,903	175,768
0	36,749	12,114	24,635	144,803
0	29,593	13,444	16,149	94,923
0	33,525	9,269	24,256	142,575
0	23,402	17,292	6,110	35,914
0	38,473	13,591	24,882	146,255
0	26,359	15,425	10,934	64,269
0	23,400	13,134	10,266	60,343
0	24,061	13,557	10,504	61,742
0	19,514	13,685	5,829	34,263
0	31,667	12,474	19,193	112,815
0	25,754	10,718	15,036	88,381
0	27,857	10,324	17,533	103,058
0	29,745	16,240	13,505	79,382
0	35,528	11,916	23,612	138,790
0	20,954	13,927	7,027	41,304
0	26,458	11,345	15,113	88,833
0	23,126	10,543	12,583	73,962
0	28,813	8,090	20,723	121,809
0	23,001	10,673	12,328	72,463
0	19,632	10,200	9,432	55,441
0	27,145	12,248	14,897	87,564
0	25,824	12,803	13,021	76,537
0	21,857	10,246	11,611	68,249
0	22,820	11,032	11,788	69,289
0	27,721	8,890	18,831	110,688
0	21,960	8,853	13,107	77,042
0	23,953	8,440	15,513	91,185
0	24,447	11,717	12,730	74,826
0	25,818	12,247	13,571	79,770

100	2,743	7,373	0	0
100	2,423	8,486	0	0
100	3,734	6,477	0	0
200	0,185	2,962	0	0
200	1,424	4,116	0	0
200	0,143	4,242	0	0
200	0,867	5,263	0	0
200	1,038	3,634	0	0
200	0,574	4,007	0	0
200	0,292	3,986	0	0
400	0,053	2,244	0	0
400	0,027	2,099	0	0
400	0,017	1,985	0	0
400	0,113	2,43	0	0
400	0,028	2,589	0	0
400	0,202	2,529	0	0
400	0,222	3,758	0	0
400	0,036	2,449	0	0

NAcGal	IM CA	IM BKG	INT-BKG	RI%
0	18,9	6,301	12,599	61,00682
0	24,977	7,978	16,999	82,31248
0	23,415	7,177	16,238	78,62757
0	23,767	5,255	18,512	89,63872
0	29,3	3,87	25,43	123,137
0	27,653	5,517	22,136	107,1868
0	28,779	4,586	24,193	117,1472
0	29,748	5,912	23,836	115,4186
0	29,56	3,763	25,797	124,9141
0	31,519	5,25	26,269	127,1996
0	26,651	6,252	20,399	98,77594
0	26,074	5,42	20,654	100,0107
0	18,899	4,663	14,236	68,93349
0	28,69	5,339	23,351	113,0701
0	27,302	7,085	20,217	97,89466
0	35,133	5,629	29,504	142,8641
0	25,815	5,153	20,662	100,0494
0	23,88	4,765	19,115	92,55857
0	17,367	5,13	12,237	59,25395
50	18,149	7,001	11,148	53,98079
50	23,954	5,291	18,663	90,36989
50	22,741	5,076	17,665	85,53738
50	30,316	6,579	23,737	114,9392
50	28,829	4,623	24,206	117,2102
50	25,464	4,758	20,706	100,2625
50	28,795	7,118	21,677	104,9643
50	33,928	5,511	28,417	137,6007
50	37,253	4,957	32,296	156,3835
50	25,362	6,985	18,377	88,98502
50	17,505	8,127	9,378	45,41011
50	27,866	6,121	21,745	105,2935
50	17,606	6,332	11,274	54,59091
50	25,905	5,796	20,109	97,37171
50	22,175	5,173	17,002	82,32701
100	58,414	16,467	41,947	203,1156
100	63,021	22,295	40,726	197,2032
100	28,665	8,74	19,925	96,48074
100	55,06	8,21	46,85	226,8569
100	40,876	9,588	31,288	151,5026
100	31,748	7,666	24,082	116,6097
100	43,859	8,098	35,761	173,1617
100	49,304	9,407	39,897	193,1891
100	31,077	5,863	25,214	122,0911
100	38,835	21,853	16,982	82,23016
100	45,596	18,655	26,941	130,4536
100	38,224	8,932	29,292	141,8376
100	51,284	7,853	43,431	210,3014
100	35,781	7,607	28,174	136,424
100	40,919	9,185	31,734	153,6622
100	48,561	11,102	37,459	181,3838
100	36,601	10,381	26,22	126,9624

0	44,674	1,655	43,02	246,12
0	20,772	1,376	19,40	110,97
0	25,273	1,876	23,40	133,86
0	15,345	1,332	14,01	80,17
0	12,231	1,253	10,98	62,81
0	21,868	1,473	20,40	116,68
0	12,854	1,466	11,39	65,15
0	46,264	1,638	44,63	255,32
0	10,548	1,739	8,81	50,40
0	26,457	1,649	24,81	141,93
0	7,258	1,516	5,74	32,85
0	10,778	1,482	9,30	53,18
0	10,548	1,492	9,06	51,81
0	20,722	1,168	19,55	111,87
50	9,233	4,653	4,58	26,20
50	10,891	4,651	6,24	35,70
50	13,199	5,069	8,13	46,51
50	26,888	4,291	22,60	129,28
50	9,742	2,335	7,41	42,38
50	29,721	3,955	25,77	147,41
50	15,492	4,083	11,41	65,27
50	13,332	4,333	9,00	51,49
50	11,587	4,338	7,25	41,47
50	8,871	1,894	6,98	39,92
50	9,307	3,54	5,77	32,99
50	15,201	3,151	12,05	68,94
50	25,633	2,179	23,45	134,19
50	9,761	2,023	7,74	44,27
50	10,09	2,747	7,34	42,01
50	24,132	2,98	21,15	121,02
50	12,101	1,833	10,27	58,75
50	17,474	2,882	14,59	83,48
50	16,998	2,243	14,76	84,42
50	14,013	3,406	10,61	60,69
50	21,741	2,496	19,25	110,11
50	10,29	2,823	7,47	42,72
50	12,698	1,695	11,00	62,95
50	8,321	2,142	6,18	35,35
50	11,914	2,178	9,74	55,70
50	15	1,813	13,19	75,45
50	14,567	2,615	11,95	68,38
50	10,584	1,687	8,90	50,90
50	8,02	1,718	6,30	36,06
50	11,658	2,522	9,14	52,27
50	21,184	1,877	19,31	110,46
50	6,821	1,507	5,31	30,40
100	9,38	1,329	8,05	46,06
100	7,217	2,096	5,12	29,30
100	23,929	1,631	22,30	127,57
100	39,831	1,249	38,58	220,74
100	21,502	1,358	20,14	115,25
100	14,451	1,446	13,01	74,40
100	24,716	1,86	22,86	130,76
100	16,982	2,309	14,67	83,95
100	14,038	1,96	12,08	69,10
100	11,167	1,654	9,51	54,43
100	23,606	1,655	21,95	125,59
100	14,143	1,614	12,53	71,68
100	6,502	1,318	5,18	29,66
100	21,042	1,395	19,65	112,41
100	26,593	1,323	25,27	144,58
100	34,531	1,167	33,36	190,88
100	23,551	1,429	22,12	126,57
100	30,49	2,524	27,97	160,00
100	23,92	1,876	22,04	126,12
100	10,157	1,736	8,42	48,18
100	26,618	1,852	24,77	141,69
100	20,477	1,123	19,35	110,73
100	14,73	1,072	13,66	78,14
100	11,112	1,149	9,96	57,00
100	27,447	1,816	25,63	146,64

400	2,472	3,476	0,00	0,00
400	1,712	1,962	0,00	0,00
400	1,555	2,974	0,00	0,00
400	1,234	2,523	0,00	0,00
400	2,258	2,963	0,00	0,00
400	2,174	3,757	0,00	0,00
400	2,124	3,747	0,00	0,00
400	2,409	4,082	0,00	0,00
400	2,7	3,04	0,00	0,00
400	1,366	1,979	0,00	0,00
400	1,756	1,72	0,04	0,08
400	2,796	4,123	0,00	0,00
400	4,401	4,178	0,22	0,48
400	4,803	3,774	1,03	2,22
400	2,366	3,527	0,00	0,00
400	2,077	3,261	0,00	0,00
400	3,628	4,091	0,00	0,00
400	1,523	2,748	0,00	0,00
400	2,847	3,621	0,00	0,00
400	2,55	5,593	0,00	0,00
400	5,605	3,27	2,34	5,03
400	3,598	2,851	0,75	1,61
400	3,174	2,172	1,00	2,16
400	3,19	3,446	0,00	0,00
400	3,204	3,64	0,00	0,00

Gal	IM CA	IM BKG	IF-BKG	RI%
0	18,346	2,366	15,98	34,71
0	55,343	4,255	51,09	110,97
0	43,043	4,383	38,66	83,98
0	72,615	3,074	69,54	151,06
0	54,025	8,149	45,88	99,65
0	52,485	7,134	45,35	98,51
0	69,846	6,26	63,59	138,12
0	56,416	4,972	51,44	111,75
0	70,676	6,689	63,99	138,99
0	56,566	6,156	50,41	109,50
0	64,541	6,014	58,53	127,13
0	46,541	6,386	40,16	87,22
0	65,802	5,826	59,98	130,28
0	35,69	6,798	28,89	62,76
0	72,03	4,869	67,16	145,89
0	61,376	4,496	56,88	123,55
0	65,224	4,281	60,94	132,38
0	58,765	4,696	54,07	117,45
0	58,682	6,197	52,49	114,01
0	50,252	13,669	36,58	79,47
0	48,731	13,99	34,74	75,46
0	54,438	13,682	40,76	88,53
0	49,751	13,222	36,53	79,35
0	64,603	4,36	60,24	130,86
0	59,4	5,024	54,38	118,12
0	50,073	6,829	43,24	93,93
0	54,279	5,415	48,86	106,14
0	38,971	6,148	32,82	71,30
0	66,953	16,77	50,18	109,01
0	39,41	3,985	35,43	76,95
0	33,479	5,988	27,49	59,72
0	37,765	5,324	32,44	70,47
0	49,868	4,33	45,54	98,92
0	42,411	8,289	34,12	74,12
0	36,809	5,271	31,54	68,51
0	43,464	5,848	37,62	81,71
0	30,797	5,973	24,82	53,92
0	44,696	5,692	39,00	84,72
0	26,595	10,726	15,87	34,47
0	30,109	6,801	23,31	50,63
0	46,666	7,503	39,16	85,07
0	51,226	1,801	49,43	107,36
0	36,036	1,809	34,23	74,35
0	53,29	2,002	51,29	111,41

100	4,303	9,239	0,000	0,000
100	3,644	7,819	0,000	0,000
100	4,533	8,342	0,000	0,000
100	3,369	7,227	0,000	0,000
100	3,473	7,818	0,000	0,000
100	4,106	4,290	0,000	0,000
100	3,170	4,925	0,000	0,000
100	1,517	4,479	0,000	0,000
100	1,428	4,416	0,000	0,000
100	1,276	4,680	0,000	0,000
200	3,538	7,810	0,000	0,000
200	2,908	7,322	0,000	0,000
200	2,565	5,994	0,000	0,000
200	3,569	11,775	0,000	0,000
200	3,225	7,909	0,000	0,000
200	4,167	8,936	0,000	0,000
200	3,303	7,052	0,000	0,000
200	4,040	8,671	0,000	0,000
200	3,385	8,716	0,000	0,000
200	3,146	9,157	0,000	0,000
200	2,435	7,467	0,000	0,000
200	1,758	8,631	0,000	0,000
200	2,545	10,445	0,000	0,000
200	2,312	9,435	0,000	0,000
200	3,017	7,684	0,000	0,000
200	2,434	6,264	0,000	0,000
200	3,377	5,914	0,000	0,000
200	3,129	5,999	0,000	0,000
200	3,160	4,993	0,000	0,000
200	3,038	8,473	0,000	0,000
200	2,911	6,097	0,000	0,000
200	2,717	5,374	0,000	0,000
200	3,744	9,019	0,000	0,000
200	3,087	7,481	0,000	0,000
200	6,161	7,673	0,000	0,000
200	2,611	6,817	0,000	0,000
200	3,524	6,474	0,000	0,000
200	3,304	6,976	0,000	0,000
200	2,376	7,121	0,000	0,000
200	3,326	9,457	0,000	0,000
200	3,530	5,760	0,000	0,000
200	3,586	4,495	0,000	0,000
200	3,007	4,149	0,000	0,000
200	2,826	6,862	0,000	0,000
200	2,281	4,487	0,000	0,000
200	3,359	5,641	0,000	0,000
400	3,287	12,183	0,000	0,000
400	4,485	10,866	0,000	0,000
400	3,088	10,262	0,000	0,000
400	4,596	13,260	0,000	0,000
400	5,265	13,167	0,000	0,000
400	5,923	16,973	0,000	0,000
400	3,685	11,217	0,000	0,000
400	3,226	10,207	0,000	0,000
400	3,593	7,830	0,000	0,000
400	2,015	4,456	0,000	0,000
400	3,954	7,593	0,000	0,000
400	3,682	9,175	0,000	0,000
400	0,696	5,064	0,000	0,000
400	1,071	4,361	0,000	0,000
400	1,197	4,769	0,000	0,000
400	0,626	5,094	0,000	0,000
400	1,269	6,476	0,000	0,000
400	2,270	5,608	0,000	0,000
400	2,011	6,540	0,000	0,000
400	2,188	5,239	0,000	0,000
400	0,546	4,729	0,000	0,000
400	0,381	6,425	0,000	0,000
400	1,345	6,287	0,000	0,000
400	2,559	11,865	0,000	0,000
400	1,614	5,586	0,000	0,000

0	13,369	6,564	6,805	39,999
0	25,669	6,735	18,934	111,293
0	26,077	6,704	19,373	113,873
0	21,843	8,980	12,863	75,608
0	21,544	9,416	12,128	71,288
0	18,200	7,997	10,203	59,973
0	22,247	10,144	12,103	71,141
0	16,027	9,573	6,454	37,936
0	21,812	7,360	14,452	84,948
0	24,194	7,204	16,990	99,866
0	19,008	6,704	12,304	72,322
0	25,090	5,960	19,130	112,445
0	22,995	10,920	12,075	70,976
0	30,730	7,656	23,074	135,628
0	27,018	7,545	19,473	114,461
0	27,082	8,613	18,469	108,560
0	31,690	9,522	22,168	130,302
0	41,976	10,661	31,315	184,068
0	19,917	8,016	11,901	69,953
0	29,828	8,987	20,841	122,502
0	42,151	11,738	30,413	178,766
0	33,542	11,045	22,497	132,236
0	36,308	13,054	23,254	136,686
0	29,012	9,760	19,252	113,162
0	29,909	11,213	18,696	109,894
0	27,159	10,875	16,284	95,716
0	30,385	8,713	21,672	127,387
0	24,817	7,977	16,840	98,985
0	26,729	8,663	18,066	106,191
200	13,145	11,335	1,810	10,639
200	12,840	11,939	0,901	5,296
200	19,369	12,990	6,379	37,495
200	7,128	12,866	0,000	0,000
200	9,385	14,984	0,000	0,000
200	8,944	10,322	0,000	0,000
200	12,594	19,159	0,000	0,000
200	21,837	13,436	8,401	49,381
200	14,114	10,319	3,795	22,307
200	18,629	12,309	6,320	37,149
200	22,059	8,733	13,326	78,330
200	19,755	12,026	7,729	45,431
200	22,440	11,161	11,279	66,297
200	21,965	13,518	8,447	49,651
200	9,765	11,247	0,000	0,000
200	21,200	14,212	6,988	41,075
200	21,501	12,635	8,866	52,114
200	28,149	10,403	17,746	104,310
200	14,524	11,094	3,430	20,161
200	26,174	10,769	15,405	90,550
200	19,346	13,707	5,639	33,146
200	13,264	11,340	1,924	11,309
200	14,675	9,665	5,010	29,449
200	10,268	10,996	0,000	0,000
200	13,305	11,669	1,636	9,616
200	11,118	9,329	1,789	10,516
200	10,435	9,299	1,136	6,677
200	9,323	10,995	0,000	0,000
200	7,375	9,814	0,000	0,000
200	8,880	10,049	0,000	0,000
200	6,621	7,098	0,000	0,000
400	6,476	8,792	0,000	0,000
400	10,041	8,867	1,174	6,901
400	7,368	10,546	0,000	0,000
400	7,693	11,562	0,000	0,000
400	8,879	8,538	0,341	2,004
400	10,006	9,480	0,526	3,092
400	8,062	10,348	0,000	0,000
400	9,599	10,040	0,000	0,000
400	3,330	7,587	0,000	0,000
400	4,439	7,757	0,000	0,000
400	3,837	8,517	0,000	0,000

100	43,816	11,285	32,531	157,5215
100	27,064	5,338	21,726	105,2015
100	28,193	9,697	18,496	89,56125
100	34,749	5,411	29,338	142,0603
100	39,128	6,801	32,327	156,5337
100	33,403	3,207	30,196	146,2149
100	25,032	4,373	20,659	100,0349
100	28,777	6,071	22,706	109,9469
100	35,665	8,191	27,474	133,0345
100	28,353	14,622	13,731	66,48819
100	31,109	13,103	18,006	87,18857
100	36,034	9,074	26,96	130,5456
100	30,086	8,25	21,836	105,7342
100	34,227	9,524	24,703	119,6168
100	34,979	12,768	22,211	107,55
100	23,3	9,622	13,678	66,23155
200	23,465	5,169	18,296	88,59281
200	19,687	4,01	15,677	75,9111
200	17,199	7,934	9,265	44,86294
200	28,896	8,456	20,44	98,97447
200	30,458	15,127	15,331	74,2357
200	30,344	14,332	16,012	77,53323
200	28,861	17,481	11,38	55,10418
200	37,975	9,823	28,152	136,3175
200	36,956	7,079	29,877	144,6703
200	40,52	8,499	32,021	155,0519
200	34,745	6,367	28,378	137,4118
200	23,636	4,841	18,795	91,00906
400	51,958	9,48	42,478	205,6868
400	51,64	11,344	40,296	195,1211
400	61,423	11,468	49,955	241,8919
400	42,736	13,854	28,882	139,8523
400	31,387	14,874	16,513	79,95917
400	36,797	15,288	21,509	104,1508
400	63,389	19,265	44,124	213,657
400	51,049	21,682	29,367	142,2008
400	38,066	21,804	16,262	78,74378
400	34,607	11,636	22,971	111,2301
400	41,312	7,912	33,4	161,7293
400	47,368	6,447	40,921	198,1475
400	26,711	7,136	19,575	94,78597
400	56,071	8,121	47,95	232,1833
400	50,179	8,81	41,369	200,3168
400	55,1	6,185	48,915	236,856
400	52,839	9,215	43,624	211,2359
400	43,05	10,306	32,744	158,5528
400	60,463	9,557	50,906	246,4968
400	52,073	7,347	44,726	216,572
400	46,976	10,895	36,081	174,7113
400	55,14	13,654	41,486	200,8833
400	50,331	7,603	42,728	206,8973
400	46,613	6,879	39,734	192,3998
400	35,553	7,876	27,677	134,0174
400	49,17	9,444	39,726	192,3611
400	41,41	9,291	32,119	155,5265
400	45,957	8,827	37,13	179,7907
400	36,712	9,897	26,815	129,8435
400	36,019	8,052	27,967	135,4217
400	47,1	14,477	32,623	157,9669
400	41,958	7,777	34,181	165,5111
400	50,503	9,949	40,554	196,3704
400	40,853	10,429	30,424	147,319
400	38,777	10,797	27,98	135,4846
400	39,04	9,108	29,932	144,9366
400	51,007	11,122	39,885	193,131
400	44,433	7,42	37,013	179,2242
400	43,726	10,011	33,715	163,2546
400	37,393	6,05	31,343	151,7689
400	41,529	6,793	34,736	168,1985
400	44,977	5,868	39,109	189,3734
400	44,502	6,275	38,227	185,1026

100	23,612	1,457	22,16	126,75
100	9,122	1,218	7,90	45,22
100	6,73	1,26	5,47	31,30
100	11,798	1,545	10,25	58,66
100	31,018	1,497	29,52	168,90
100	8,103	1,343	6,76	38,68
100	44,246	1,664	42,58	243,62
100	35,722	1,348	34,37	196,66
100	16,94	1,324	15,62	89,34
100	25,034	1,197	23,84	136,38
100	27,037	1,2	25,84	147,82
100	12,972	1,22	11,75	67,24
100	20,453	1,143	19,31	110,48
100	22,329	0,999	21,33	122,03
100	28,776	1,071	27,71	158,51
100	21,083	1,261	19,82	113,41
100	25,103	1,496	23,61	135,06
100	13,911	1,39	12,52	71,64
100	25,175	1,212	23,96	137,10
100	28,397	1,261	27,14	155,25
100	41,49	1,281	40,21	230,05
100	27,342	1,239	26,10	149,34
100	34,775	1,312	33,46	191,45
100	20,124	1,164	18,96	108,47
100	40,546	1,373	39,17	224,12
100	25,585	1,282	24,30	139,04
100	18,637	1,513	17,12	97,97
100	13,279	1,541	11,74	67,16
100	38,833	1,556	37,28	213,27
100	10,678	1,055	9,62	55,06
200	21,518	1,304	20,21	115,65
200	7,042	1,738	5,30	30,35
200	20,467	1,458	19,01	108,76
200	14,499	1,388	13,11	75,01
200	19,774	1,88	17,89	102,38
200	18,273	1,603	16,67	95,37
200	18,675	1,512	17,16	98,19
200	29,766	1,639	28,13	160,92
200	12,807	1,465	11,34	64,89
200	11,992	1,269	10,72	61,35
200	7,841	1,521	6,32	36,16
200	19,697	1,342	18,36	105,01
200	11,632	1,541	10,09	57,73
200	35,744	1,244	34,50	197,38
200	12,161	1,781	10,38	59,39
200	26,216	2,02	24,20	138,43
200	22,51	2,072	20,44	116,93
200	35,213	1,954	33,26	190,28
200	42,99	1,978	41,01	234,64
200	27,744	1,579	26,17	149,70
200	29,501	1,375	28,13	160,92
200	22,167	1,269	20,90	119,56
200	31,634	1,329	30,31	173,38
200	18,52	1,086	17,43	99,74
200	18,819	1,278	17,54	100,36
200	16,717	1,163	15,55	88,99
200	50,415	1,494	48,92	279,89
200	34,522	1,388	33,13	189,57
200	36,231	1,493	34,74	198,74
200	27,961	1,453	26,51	151,66
200	20,489	1,429	19,06	109,05
200	25,645	1,327	24,32	139,13
200	44,162	1,775	42,39	242,51
200	43,722	2,254	41,47	237,25
200	12,987	1,861	11,13	63,65
200	10,3	1,558	8,74	50,02
200	22,51	1,406	21,10	120,74
200	49,284	1,475	47,81	273,53
200	13,172	1,359	11,81	67,59
200	14,323	1,552	12,77	73,07
200	21,666	1,72	19,95	114,12

0	51,437	2,355	49,08	106,62
0	56,593	1,986	54,61	118,62
0	50,351	2,998	47,35	102,86
0	73,306	3,838	69,47	150,90
0	20,552	2,491	18,06	39,23
0	57,541	3,791	53,75	116,76
0	58,46	3,61	54,85	119,15
0	53,75	3,47	50,28	109,22
0	30,678	2,484	28,19	61,24
0	57,175	2,455	54,72	118,86
0	45,484	3,026	42,46	92,23
0	62,21	3,555	58,66	127,41
0	62,203	2,352	59,85	130,01
0	45,876	1,762	44,11	95,82
0	39,289	3,067	36,22	78,68
0	50,356	2,042	48,31	104,95
0	69,865	2,234	67,63	146,91
0	55,471	2,001	53,47	116,15
0	43,056	1,83	41,23	89,55
0	73,207	2,375	70,83	153,86
0	56,509	2,053	54,46	118,29
0	47,945	1,776	46,17	100,29
50	36,828	3,692	33,14	71,98
50	52,012	3,804	48,21	104,72
50	33,487	7,895	25,59	55,59
50	33,132	7,266	25,87	56,19
50	49,101	4,175	44,93	97,59
50	24,812	1,74	23,07	50,12
50	28,624	2,229	26,40	57,34
50	33,671	2,11	31,56	68,56
50	30,559	3,062	27,50	59,73
50	28,038	2,267	25,77	55,98
50	32,428	5,599	26,83	58,28
50	28,495	3,159	25,34	55,03
50	36,517	5,213	31,30	68,00
50	25,1	4,903	20,20	43,87
50	29,459	2,865	26,59	57,77
50	25,064	3,581	21,48	46,67
50	20,707	3,152	17,56	38,13
50	16,306	2,794	13,51	29,35
50	15,639	2,86	12,78	27,76
50	17,464	2,809	14,66	31,83
50	20,811	4,216	16,60	36,05
50	38,67	5,72	32,95	71,57
50	10,056	1,743	8,31	18,06
50	36,174	4,154	32,02	69,55
50	22,944	3,11	19,83	43,08
50	20,135	3,424	16,71	36,30
50	37,66	2,281	35,38	76,85
50	51,099	2,396	48,70	105,79
50	26,66	1,142	25,52	55,43
50	32,963	4,118	28,85	62,66
50	28,815	2,613	26,20	56,92
50	37,555	2,936	34,62	75,20
50	36,14	5,661	30,48	66,21
50	27,565	0,853	26,71	58,02
50	24,53	0,484	24,05	52,23
50	25,939	1,186	24,75	53,77
50	49,773	0,649	49,12	106,71
50	46,002	3,661	42,34	91,97
50	32,173	2,246	29,93	65,01
50	20,987	3,183	17,80	38,67
50	47,139	2,895	44,24	96,11
50	25,075	2,012	23,06	50,10
50	24,945	1,89	23,06	50,08
100	18,106	2,628	15,48	33,62
100	24,448	2,3	22,15	48,11
100	13,778	3,865	9,91	21,53
100	15,086	2,435	12,65	27,48
100	29,731	2,043	27,69	60,14
100	35,841	1,874	33,97	73,78

400	1,044	6,560	0,000	0,000
400	2,869	9,083	0,000	0,000
400	1,248	6,703	0,000	0,000
400	2,244	7,588	0,000	0,000
400	1,230	6,524	0,000	0,000
400	2,880	6,597	0,000	0,000
400	1,492	6,019	0,000	0,000

NAcGlc	RI	BKG	RI-BKG	RI%
0	12,001	4,397	7,604	128,450
0	14,977	3,007	11,970	202,202
0	13,404	2,842	10,562	178,417
0	9,149	3,229	5,920	100,003
0	11,472	4,955	6,517	110,088
0	11,219	4,022	7,197	121,574
0	7,439	6,311	1,128	19,055
0	13,475	4,436	9,039	152,690
0	11,883	4,256	7,627	128,838
0	10,272	4,445	5,827	98,432
0	11,599	3,638	7,961	134,480
0	11,272	2,822	8,450	142,741
0	7,599	3,401	4,198	70,914
0	9,404	3,600	5,804	98,043
0	11,910	4,217	7,693	129,953
0	19,761	5,489	14,272	241,088
0	17,384	4,551	12,833	216,780
0	16,511	6,115	10,396	175,613
0	11,892	4,407	7,485	126,439
0	5,816	3,465	2,351	39,714
0	13,606	4,018	9,588	161,964
0	7,697	2,745	4,952	83,651
0	5,439	3,267	2,172	36,690
0	4,918	3,228	1,690	28,548
0	4,075	2,826	1,249	21,099
0	4,477	2,750	1,727	29,173
0	4,993	2,659	2,334	39,427
0	7,370	3,412	3,958	66,860
0	6,597	4,004	2,593	43,802
0	9,215	3,693	5,522	93,280
0	13,530	4,658	8,872	149,869
0	10,342	4,099	6,243	105,459
0	8,511	3,153	5,358	90,509
0	10,214	3,549	6,665	112,588
0	7,646	3,173	4,473	75,560
0	16,707	3,135	13,572	229,263
0	14,734	10,453	4,281	72,316
0	16,876	5,440	11,436	193,181
0	12,109	3,587	8,522	143,957
0	8,672	4,423	4,249	71,776
0	8,475	4,081	4,394	74,225
0	6,461	4,773	1,688	28,514
0	6,097	4,506	1,591	26,876
0	9,965	5,307	4,658	78,685
0	6,705	4,760	1,945	32,856
0	7,373	4,383	2,990	50,508
0	9,084	5,840	3,244	54,799
0	9,037	6,389	2,648	44,731
0	21,924	9,830	12,094	204,296
0	9,093	5,126	3,967	67,012
0	8,623	4,563	4,060	68,583
0	8,054	4,422	3,632	61,353
0	7,123	4,573	2,550	43,076
50	9,065	6,037	3,028	51,150
50	4,251	3,570	0,681	11,504
50	7,379	3,977	3,402	57,468
50	4,970	2,603	2,367	39,984
50	11,058	4,219	6,839	115,527
50	5,529	3,829	1,700	28,717
50	9,912	5,357	4,555	76,945
50	7,900	6,286	1,614	27,264
50	11,855	6,465	5,390	91,050

400	3,395	5,983	0,000	0,000
400	3,995	10,513	0,000	0,000
400	3,260	9,627	0,000	0,000
400	4,507	6,460	0,000	0,000
400	3,252	8,880	0,000	0,000
400	4,517	7,791	0,000	0,000
400	3,985	9,682	0,000	0,000
400	4,863	10,146	0,000	0,000
400	4,864	8,763	0,000	0,000
400	5,605	8,013	0,000	0,000
400	6,162	8,164	0,000	0,000
400	6,232	7,459	0,000	0,000
400	6,014	7,577	0,000	0,000
400	6,988	8,969	0,000	0,000
400	11,526	10,917	0,609	3,580
400	6,114	6,303	0,000	0,000
400	7,359	7,862	0,000	0,000
400	7,104	7,546	0,000	0,000
400	8,642	9,620	0,000	0,000
800	3,284	8,648	0,000	0,000
800	5,139	11,747	0,000	0,000
800	4,362	13,144	0,000	0,000
800	3,524	19,357	0,000	0,000
800	3,918	16,027	0,000	0,000
800	4,131	12,646	0,000	0,000
800	4,659	12,428	0,000	0,000
800	4,802	15,518	0,000	0,000
800	4,281	16,559	0,000	0,000
800	5,061	13,488	0,000	0,000
800	4,824	23,164	0,000	0,000
800	8,131	12,754	0,000	0,000
800	4,384	8,757	0,000	0,000
800	3,353	8,559	0,000	0,000
800	3,249	9,401	0,000	0,000
800	4,110	5,707	0,000	0,000
800	4,111	12,725	0,000	0,000
800	2,769	9,419	0,000	0,000
800	3,704	9,893	0,000	0,000
800	6,174	10,133	0,000	0,000
800	4,271	10,678	0,000	0,000
800	3,211	8,338	0,000	0,000
800	4,617	10,724	0,000	0,000
800	7,015	11,302	0,000	0,000
800	5,582	10,072	0,000	0,000
800	3,680	11,030	0,000	0,000
800	5,847	7,082	0,000	0,000
800	4,575	10,201	0,000	0,000
800	6,783	8,583	0,000	0,000
800	3,209	9,595	0,000	0,000
800	4,121	13,549	0,000	0,000

NAcGlc	RI	BKG	RI-BKG	RI%
0	37,085	11,212	25,873	152,080
0	40,619	17,761	22,858	134,358
0	24,342	12,682	11,660	68,537
0	28,129	13,010	15,119	88,869
0	32,940	17,394	15,546	91,379
0	39,134	15,251	23,883	140,383
0	39,859	10,508	29,351	172,524
0	48,173	15,260	32,913	193,461
0	43,781	13,878	29,903	175,768
0	36,749	12,114	24,635	144,803
0	29,593	13,444	16,149	94,923
0	33,525	9,269	24,256	142,575
0	23,402	17,292	6,110	35,914
0	38,473	13,591	24,882	146,255
0	26,359	15,425	10,934	64,269
0	23,400	13,134	10,266	60,343
0	24,061	13,557	10,504	61,742
0	19,514	13,685	5,829	34,263
0	31,667	12,474	19,193	112,815

400	51,854	9,107	42,747	206,9893
400	40,409	8,889	31,52	152,626
400	46,264	8,917	37,347	180,8415
400	52,156	9,951	42,205	204,3649
400	44,653	9,776	34,877	168,8812
400	55,192	6,331	48,861	236,5945
400	47,129	7,13	39,999	193,683
400	34,375	8,273	26,102	126,391
400	54,061	31,945	22,116	107,09
400	50,342	34,001	16,341	79,12632
400	72,167	21,108	51,059	247,2377
400	34,071	6,275	27,796	134,5937
400	30,723	10,927	19,796	95,8561
400	41,843	16,858	24,985	120,9823

200	19,76	1,513	18,25	104,40
200	21,867	1,544	20,32	116,27
200	39,587	1,467	38,12	218,09
200	26,809	1,406	25,40	145,34
200	41,035	1,383	39,65	226,86
200	21,803	1,311	20,49	117,24
200	9,315	1,289	8,03	45,92
200	23,421	1,353	22,07	126,26
200	14,045	1,425	12,62	72,20
200	12,126	1,194	10,93	62,54
200	13,89	1,532	12,36	70,70
200	15,852	1,313	14,54	83,18
200	4,994	1,535	3,46	19,79
200	29,284	1,679	27,61	157,93
400	2,788	2,588	0,20	1,14
400	7,028	2,298	4,73	27,06
400	14,109	2,455	11,65	66,68
400	8,528	2,199	6,33	36,21
400	16,661	2,963	13,70	78,37
400	3,626	0,439	3,19	18,23
400	4,107	1,101	3,01	17,20
400	7,39	1,182	6,21	35,52
400	21,021	2,406	18,62	106,50
400	28,749	2,234	26,52	151,70
400	4,789	1,724	3,07	17,54
400	14,961	2,797	12,16	69,59
400	18,375	1,415	16,96	97,03
400	6,842	1,942	4,90	28,03
800	8,384	1,406	6,98	39,92
800	8,834	1,676	7,16	40,95
800	8,467	1,682	6,79	38,82
800	8,606	1,397	7,21	41,24
800	9,372	2,239	7,13	40,81
800	4,91	2,245	2,67	15,25
800	26,751	1,487	25,26	144,54
800	22,221	1,863	20,36	116,47
800	10,871	2,105	8,77	50,15
800	15,811	1,726	14,09	80,58
800	11,29	2,427	8,86	50,71
800	19,588	1,712	17,88	102,27
800	6,763	2,101	4,66	26,67
800	37,665	2,277	35,39	202,46
800	35,177	2,473	32,70	187,11
800	7,013	1,977	5,04	28,81
800	8,169	3,253	4,92	28,13
800	5,149	1,958	3,19	18,26
800	8,249	2,062	6,19	35,40
800	9,253	1,316	7,94	45,41
800	20,937	1,471	19,47	111,37
800	31,545	3,024	28,52	163,18
800	16,559	5,792	10,77	61,60
800	12,928	5,273	7,66	43,80
800	19,919	1,602	18,32	104,80
800	8	1,617	6,38	36,52
800	15,086	1,749	13,34	76,30
800	29,013	1,91	27,10	155,06
800	15,519	1,469	14,05	80,38
800	36,678	1,578	35,10	200,82
800	16,64	2,433	14,21	81,28
800	20,468	2,396	18,07	103,39
800	10,517	3,19	7,33	41,92
800	30,376	2,534	27,84	159,29

Man	IM CA	IM BKG	CA-BKG	RI%
0	17,941	1,18	16,76	72,27
0	14,896	1,56	13,34	57,50
0	20,267	1,105	19,16	82,62
0	11,695	1,155	10,54	45,45
0	12,994	1,099	11,90	51,29
0	18,422	1,117	17,31	74,62
0	25,79	1,451	24,34	104,95

100	28,908	3,004	25,90	56,27
100	19,145	2,5	16,65	36,16
100	26,421	3,497	22,92	49,80
100	30,734	3,799	26,94	58,51
100	28,746	3,672	25,07	54,47
100	22,454	3,335	19,12	41,53
100	23,845	3,596	20,25	43,98
100	26,755	4,179	22,58	49,04
100	22,416	2,319	20,10	43,65
100	23,512	2,759	20,75	45,08
100	29,714	4,854	24,86	54,00
100	25,787	3,286	22,50	48,88
100	21,963	3,252	18,71	40,64
100	22,749	4,677	18,07	39,26
100	25,337	4,168	21,17	45,98
100	31,241	3,5	27,74	60,26
100	18,839	3,139	15,70	34,10
100	32,216	5,378	26,84	58,30
100	13,553	3,466	10,09	21,91
100	18,107	2,18	15,93	34,60
100	14,533	1,945	12,59	27,34
100	14,567	1,679	12,89	28,00
100	17,509	4,852	12,66	27,49
100	20,585	4,25	16,34	35,48
100	22,119	3,624	18,50	40,17
100	24,868	3,727	21,14	45,92
100	21,419	4,114	17,31	37,59
200	8,486	1,966	6,52	14,16
200	7,868	2,311	5,56	12,07
200	2,794	1,563	1,23	2,67
200	7,61	2,133	5,48	11,90
200	4,994	2,432	2,56	5,57
200	8,318	2,336	5,98	12,99
200	2,865	2,033	0,83	1,81
200	5,669	2,66	3,01	6,54
200	2,47	1,457	1,01	2,20
200	6,245	1,83	4,42	9,59
200	4,081	1,476	2,61	5,66
200	6,21	2,212	4,00	8,68
200	6,113	1,209	4,90	10,65
200	5,834	1,684	4,15	9,01
200	4,072	1,27	2,80	6,09
200	6,112	2,336	3,78	8,20
200	6,061	2,4	3,66	7,95
200	4,419	1,494	2,93	6,35
200	4,91	1,464	3,45	7,49
200	3,765	1,369	2,40	5,20
200	6,245	1,205	5,04	10,95
200	6,1	1,525	4,58	9,94
200	3,74	1,237	2,50	5,44
200	3,587	1,851	1,74	3,77
400	1,497	1,146	0,35	0,76
400	1,637	1,822	0,00	0,00
400	1,161	0,908	0,25	0,55
400	1,016	1,509	0,00	0,00
400	1,059	1,689	0,00	0,00
400	0,886	1,025	0,00	0,00
400	1,007	1,19	0,00	0,00

50	7,708	4,868	2,840	47,974
50	5,652	7,005	0,000	0,000
50	6,196	4,975	1,221	20,626
50	5,541	6,428	0,000	0,000
50	4,648	5,337	0,000	0,000
50	8,058	6,184	1,874	31,656
50	5,321	6,547	0,000	0,000
50	8,525	6,636	1,889	31,910
50	7,869	6,430	1,439	24,308
50	6,949	6,257	0,692	11,690
50	6,742	6,338	0,404	6,825
50	8,098	7,199	0,899	15,186
50	11,298	7,295	4,003	67,620
50	7,756	6,950	0,806	13,615
50	13,213	6,811	6,402	108,145
50	5,773	3,748	2,025	34,207
50	13,243	7,487	5,756	97,233
50	9,424	4,267	5,157	87,114
50	11,728	5,062	6,666	112,605
50	7,909	4,876	3,033	51,235
50	11,320	5,321	5,999	101,337
50	7,452	6,823	0,629	10,625
50	13,480	7,294	6,186	104,496
50	10,036	7,302	2,734	46,184
50	18,844	13,322	5,522	93,280
50	9,711	9,720	0,000	0,000
50	15,301	6,560	8,741	147,656
50	14,157	8,187	5,970	100,847
100	7,030	5,816	1,214	20,507
100	6,030	5,123	0,907	15,321
100	8,816	5,019	3,797	64,140
100	15,235	7,058	8,177	138,129
100	9,160	9,715	0,000	0,000
100	14,123	7,661	6,462	109,159
100	14,605	6,722	7,883	133,163
100	9,938	6,138	3,800	64,191
100	16,070	6,868	9,202	155,444
100	13,399	6,384	7,015	118,500
100	14,298	7,392	6,906	116,659
100	6,651	4,433	2,218	37,467
100	5,444	4,928	0,516	8,716
100	5,875	4,830	1,045	17,653
100	5,544	4,587	0,957	16,166
100	5,273	4,973	0,300	5,068
100	5,886	4,854	1,032	17,433
100	5,939	5,385	0,554	9,358
100	5,679	4,025	1,654	27,940
100	5,606	4,632	0,974	16,453
100	9,615	5,208	4,407	74,445
100	5,800	5,196	0,604	10,203
100	6,763	4,201	2,562	43,278
100	6,991	5,454	1,537	25,964
100	4,840	4,653	0,187	3,159
100	4,633	4,200	0,433	7,314
100	5,704	5,333	0,371	6,267
100	9,540	5,359	4,181	70,627
100	8,229	2,930	5,299	89,513
100	5,015	2,282	2,733	46,167
100	6,378	2,796	3,582	60,508
100	6,282	4,162	2,120	35,812
100	7,782	2,659	5,123	86,540
100	8,126	7,818	0,308	5,203
100	6,900	2,893	4,007	67,688
100	6,376	3,277	3,099	52,349
100	6,696	2,657	4,039	68,228
100	8,236	2,476	5,760	97,300
100	6,301	2,286	4,015	67,823
100	9,390	2,336	7,054	119,159
200	7,028	5,419	1,609	27,180
200	12,664	5,391	7,273	122,858
200	8,572	4,886	3,686	62,265

0	25,754	10,718	15,036	88,381
0	27,857	10,324	17,533	103,058
0	29,745	16,240	13,505	79,382
0	35,528	11,916	23,612	138,790
0	20,954	13,927	7,027	41,304
0	26,458	11,345	15,113	88,833
0	23,126	10,543	12,583	73,962
0	28,813	8,090	20,723	121,809
0	23,001	10,673	12,328	72,463
0	19,632	10,200	9,432	55,441
0	27,145	12,248	14,897	87,564
0	25,824	12,803	13,021	76,537
0	21,857	10,246	11,611	68,249
0	22,820	11,032	11,788	69,289
0	27,721	8,890	18,831	110,688
0	21,960	8,853	13,107	77,042
0	23,953	8,440	15,513	91,185
0	24,447	11,717	12,730	74,826
0	25,818	12,247	13,571	79,770
0	13,369	6,564	6,805	39,999
0	25,669	6,735	18,934	111,293
0	26,077	6,704	19,373	113,873
0	21,843	8,980	12,863	75,608
0	21,544	9,416	12,128	71,288
0	18,200	7,997	10,203	59,973
0	22,247	10,144	12,103	71,141
0	16,027	9,573	6,454	37,936
0	21,812	7,360	14,452	84,948
0	24,194	7,204	16,990	99,866
0	19,008	6,704	12,304	72,322
0	25,090	5,960	19,130	112,445
0	22,995	10,920	12,075	70,976
0	30,730	7,656	23,074	135,628
0	27,018	7,545	19,473	114,461
0	27,082	8,613	18,469	108,560
0	31,690	9,522	22,168	130,302
0	41,976	10,661	31,315	184,068
0	19,917	8,016	11,901	69,953
0	29,828	8,987	20,841	122,502
0	42,151	11,738	30,413	178,766
0	33,542	11,045	22,497	132,236
0	36,308	13,054	23,254	136,686
0	29,012	9,760	19,252	113,162
0	29,909	11,213	18,696	109,894
0	27,159	10,875	16,284	95,716
0	30,385	8,713	21,672	127,387
0	24,817	7,977	16,840	98,985
0	26,729	8,663	18,066	106,191
200	15,995	7,546	8,449	49,663
200	18,032	11,167	6,865	40,352
200	15,695	9,051	6,644	39,053
200	23,525	6,700	16,825	98,896
200	16,317	6,451	9,866	57,992
200	17,647	9,833	7,814	45,930
200	13,247	7,296	5,951	34,980
200	17,066	9,566	7,500	44,085
200	23,246	8,795	14,451	84,942
200	26,289	9,704	16,585	97,486
200	27,693	8,935	18,758	110,259
200	21,224	8,157	13,067	76,807
200	17,319	7,950	9,369	55,070
200	29,053	11,691	17,362	102,053
200	15,825	9,484	6,341	37,272
200	16,370	6,166	10,204	59,979
200	19,227	8,319	10,908	64,117
200	17,226	11,069	6,157	36,191
200	19,754	9,192	10,562	62,083
200	27,227	9,263	17,964	105,591
200	17,346	11,463	5,883	34,580
200	18,005	11,059	6,946	40,828
200	20,770	11,094	9,676	56,875

0	15,103	1,297	13,81	59,53
0	34,569	1,484	33,09	142,66
0	14,138	0,489	13,65	58,85
0	15,875	1,945	13,93	60,06
0	20,7	1,872	18,83	81,18
0	11,954	1,578	10,38	44,74
0	20,754	1,861	18,89	81,46
0	50,282	2,711	47,57	205,12
0	33,745	1,545	32,20	138,84
0	13,889	0,578	13,31	57,39
0	22,582	1,208	21,37	92,16
0	14,618	1,033	13,59	58,58
0	11,554	1,565	9,99	43,07
0	11,569	1,34	10,23	44,11
0	23,41	1,023	22,39	96,53
0	33,797	0,963	32,83	141,57
0	26,289	0,954	25,34	109,24
0	10,437	1,078	9,36	40,35
0	21,082	0,836	20,25	87,30
0	8,763	1,164	7,60	32,77
0	37,612	1,234	36,38	156,86
0	16,769	1,149	15,62	67,35
0	29,258	1,343	27,92	120,36
0	11,666	1,115	10,55	45,49
0	25,676	0,895	24,78	106,85
0	39,99	1,282	38,71	166,90
0	18,783	1,742	17,04	73,48
0	25,57	2,058	23,51	101,38
0	12,614	2,737	9,88	42,59
0	21,087	2,11	18,98	81,83
0	14,029	2,346	11,68	50,37
0	32,361	2,698	29,66	127,90
0	56,725	2,782	53,94	232,59
0	33,573	3,201	30,37	130,96
0	33,655	2,61	31,05	133,86
0	29,996	2,314	27,68	119,36
0	11,177	3,027	8,15	35,14
0	13,892	2,485	11,41	49,18
0	25,12	1,968	23,15	99,83
0	46,437	3,573	42,86	184,82
0	20,731	3,009	17,72	76,41
0	9,836	3,021	6,82	29,39
0	16,517	3,006	13,51	58,26
0	16,476	3,002	13,47	58,10
0	23,276	1,756	21,52	92,79
0	24,742	3,614	21,13	91,10
0	36,92	3,432	33,49	144,39
0	41,23	2,566	38,66	166,71
0	63,491	6,917	56,57	243,94
0	59,866	5,125	54,74	236,03
0	52,024	5,227	46,80	201,78
0	41,031	5,574	35,46	152,88
0	55,864	3,74	52,12	224,75
0	28,138	8,195	19,94	85,99
0	11,069	2,234	8,84	38,09
0	35,684	2,027	33,66	145,12
0	17,196	2,599	14,60	62,94
50	13,443	0,062	13,38	57,70
50	16,572	0,058	16,51	71,21
50	8,521	0,684	7,84	33,79
50	8,225	0,878	7,35	31,68
50	23,085	1,206	21,88	94,34
50	8,154	1,093	7,06	30,45
50	18,245	0,983	17,26	74,43
50	13,029	1,065	11,96	51,59
50	16,26	1,134	15,13	65,22
50	37,808	0,858	36,95	159,32
50	27,675	1,075	26,60	114,69
50	10,082	0,965	9,12	39,31
50	18,757	1,043	17,71	76,38
50	38,912	1,146	37,77	162,84

200	6,871	6,873	0,000	0,000
200	13,953	5,377	8,576	144,869
200	12,413	6,751	5,662	95,645
200	7,433	5,317	2,116	35,744
200	7,495	5,870	1,625	27,450
200	13,264	5,737	7,527	127,149
200	8,064	4,928	3,136	52,974
200	5,721	5,186	0,535	9,037
200	16,456	7,448	9,008	152,167
200	6,642	5,473	1,169	19,747
200	10,951	7,534	3,417	57,721
200	7,591	7,026	0,565	9,544
200	7,917	7,709	0,208	3,514
200	6,178	6,365	0,000	0,000
200	8,035	7,132	0,903	15,254
200	13,272	11,649	1,623	27,416
200	11,346	9,570	1,776	30,001
200	7,149	2,970	4,179	70,593
200	12,115	8,967	3,148	53,177
200	4,467	3,842	0,625	10,558
200	7,668	5,201	2,467	41,673
200	5,026	3,201	1,825	30,829
200	11,157	8,620	2,537	42,856
200	4,082	3,416	0,666	11,250
200	14,442	6,063	8,379	141,541
200	4,923	4,768	0,155	2,618
200	4,798	5,178	0,000	0,000
200	6,468	3,793	2,675	45,187
200	6,081	4,376	1,705	28,802
200	5,849	4,015	1,834	30,981
200	7,261	4,573	2,688	45,407
200	5,792	4,026	1,766	29,832
400	8,892	3,750	5,142	86,861
400	8,414	5,839	2,575	43,498
400	6,589	5,282	1,307	22,078
400	12,447	7,811	4,636	78,313
400	5,878	4,899	0,979	16,538
400	5,852	4,908	0,944	15,946
400	8,632	8,128	0,504	8,514
400	3,267	3,295	0,000	0,000
400	7,645	3,990	3,655	61,742
400	4,387	5,742	0,000	0,000
400	5,187	6,727	0,000	0,000
400	3,919	4,915	0,000	0,000
400	9,096	5,016	4,080	68,921
400	7,729	5,241	2,488	42,028
400	10,328	5,602	4,726	79,833
400	8,049	6,610	1,439	24,308
400	5,883	5,134	0,749	12,652
400	9,288	8,770	0,518	8,750
400	7,572	6,629	0,943	15,930
400	8,250	8,590	0,000	0,000
400	10,060	6,454	3,606	60,914
400	5,231	4,534	0,697	11,774
400	6,453	6,162	0,291	4,916
400	12,214	8,009	4,205	71,032
400	11,365	6,816	4,549	76,843
400	7,951	6,970	0,981	16,571
400	7,115	7,052	0,063	1,064
400	6,460	6,037	0,423	7,145
400	7,870	8,020	0,000	0,000
400	7,562	9,359	0,000	0,000
400	6,664	6,968	0,000	0,000
400	10,927	7,312	3,615	61,066
400	6,315	2,348	3,967	67,012
400	8,938	5,936	3,002	50,711
400	4,559	4,161	0,398	6,723
400	9,077	4,476	4,601	77,722
400	7,242	3,417	3,825	64,613
400	7,379	4,536	2,843	48,025
400	8,642	5,077	3,565	60,221

200	19,060	10,141	8,919	52,425
200	18,712	9,119	9,593	56,387
400	12,732	4,600	8,132	47,799
400	10,990	4,507	6,483	38,107
400	17,502	7,073	10,429	61,301
400	16,017	5,866	10,151	59,667
400	20,657	9,002	11,655	68,507
400	18,528	7,539	10,989	64,593
400	18,601	8,647	9,954	58,509
400	19,150	8,239	10,911	64,134
400	18,133	9,509	8,624	50,691
400	15,565	7,988	7,577	44,537
400	16,565	7,976	8,589	50,486
400	16,838	8,807	8,031	47,206
400	16,146	10,117	6,029	35,438
400	15,316	8,149	7,167	42,127
400	17,244	8,350	8,894	52,278
400	11,943	7,200	4,743	27,879
400	14,206	7,902	6,304	37,055
400	18,393	11,255	7,138	41,957
400	17,218	7,527	9,691	56,963
400	20,916	8,603	12,313	72,375
400	16,310	8,741	7,569	44,490
400	15,788	9,477	6,311	37,096
400	13,004	8,577	4,427	26,022
400	17,235	10,340	6,895	40,528
400	17,152	10,266	6,886	40,476
800	10,426	9,193	1,233	7,248
800	15,196	10,316	4,880	28,684
800	16,087	11,494	4,593	26,997
800	10,628	9,479	1,149	6,754
800	5,679	12,021	0,000	0,000
800	12,030	10,094	1,936	11,380
800	7,913	13,700	0,000	0,000
800	16,248	11,233	5,015	29,478
800	24,466	13,772	10,694	62,859
800	24,478	23,976	0,502	2,951
800	18,868	15,044	3,824	22,477
800	21,839	15,309	6,530	38,383
800	23,619	13,305	10,314	60,625
800	28,342	22,048	6,294	36,996
800	16,809	10,768	6,041	35,509
800	15,780	8,997	6,783	39,870
800	17,363	6,798	10,565	62,100
800	13,158	6,523	6,635	39,000

Gal	RI	BKG	RI-BKG	RI%
0	42,915	4,513	38,402	167,162
0	39,957	2,552	37,405	162,822
0	36,092	5,212	30,880	134,419
0	27,120	6,067	21,053	91,643
0	28,937	4,353	24,584	107,013
0	32,143	3,571	28,572	124,372
0	32,805	3,783	29,022	126,331
0	53,870	5,367	48,503	211,131
0	40,904	6,247	34,657	150,860
0	41,614	5,428	36,186	157,516
0	43,696	7,158	36,538	159,048
0	36,065	9,731	26,334	114,630
0	37,297	6,909	30,388	132,277
0	46,197	7,851	38,346	166,918
0	35,676	8,219	27,457	119,519
0	47,154	9,385	37,769	164,406
0	45,699	8,205	37,494	163,209
0	37,990	7,530	30,460	132,591
0	38,982	9,165	29,817	129,792
0	44,030	10,100	33,930	147,695
0	42,055	10,479	31,576	137,449
0	53,660	8,190	45,470	197,928
0	51,363	6,648	44,715	194,642
0	30,309	4,286	26,023	113,277

50	17,822	1,084	16,74	72,17
50	13,53	0,977	12,55	54,13
50	8,756	0,783	7,97	34,38
50	7,54	1,161	6,38	27,51
50	11,749	0,846	10,90	47,01
50	18,176	1,501	16,68	71,90
50	12,885	1,609	11,28	48,62
50	17,824	1,547	16,28	70,18
50	34,014	1,447	32,57	140,42
50	28,733	1,376	27,36	117,96
50	53,911	1,061	52,85	227,88
50	21,959	1,802	20,16	86,91
50	18,292	1,616	16,68	71,90
50	21,591	2,171	19,42	83,74
50	24,348	2,273	22,08	95,18
50	22,998	1,689	21,31	91,88
50	27,158	1,832	25,33	109,20
50	33,154	1,466	31,69	136,63
50	9,094	1,237	7,86	33,88
50	30,168	2,126	28,04	120,91
50	42,273	3,216	39,06	168,41
50	30,853	2,33	28,52	122,99
50	22,977	2,382	20,60	88,80
50	13,506	1,349	12,16	52,42
50	14,254	2,521	11,73	50,59
50	15,032	2,273	12,76	55,01
50	8,808	1,683	7,13	30,72
50	28,207	2,323	25,88	111,61
50	18,345	2,51	15,84	68,28
50	36,838	1,352	35,49	153,01
50	10,619	1,829	8,79	37,90
50	14,507	1,28	13,23	57,03
50	11,737	1,314	10,42	44,94
50	33,094	1,4	31,69	136,66
50	32,335	2,028	30,31	130,68
50	43,812	1,6	42,21	182,01
50	47,266	1,566	45,70	197,05
50	13,391	1,386	12,01	51,76
50	36,071	1,54	34,53	148,89
50	41,711	1,79	39,92	172,13
50	9,762	1,249	8,51	36,71
50	13,43	1,437	11,99	51,71
100	32,842	2,408	30,43	131,23
100	45,439	2,7	42,74	184,28
100	31,386	2,151	29,24	126,06
100	17,903	1,911	15,99	68,95
100	27,163	2,013	25,15	108,44
100	25,344	1,881	23,46	101,17
100	15,965	1,941	14,02	60,47
100	8,336	1,316	7,02	30,27
100	36,935	1,146	35,79	154,32
100	13,792	1,144	12,65	54,54
100	15,243	1,467	13,78	59,40
100	13,253	2,276	10,98	47,33
100	17,179	2,115	15,06	64,95
100	43,499	2,195	41,30	178,10
100	53,428	3,1	50,33	217,01
100	27,24	2,072	25,17	108,52
100	29,957	2,586	27,37	118,02
100	33,806	1,471	32,34	139,42
100	8,999	1,173	7,83	33,74
100	25,957	1,523	24,43	105,36
100	59,967	2,1	57,87	249,51
100	18,145	2,148	16,00	68,98
100	58,392	5,88	52,51	226,42
100	46,67	2,03	44,64	192,48
100	20,04	2,758	17,28	74,52
100	17,035	2,477	14,56	62,77
100	10,579	2,279	8,30	35,79
100	30,14	3,379	26,76	115,39
100	15,118	2,402	12,72	54,83

Gal	RI	BKG	RI-BKG	RI%
0	22,382	8,432	13,950	149,154
0	21,508	8,015	13,493	144,268
0	23,600	6,682	16,918	180,889
0	20,449	9,659	10,790	115,367
0	9,615	6,040	3,575	38,224
0	9,709	5,804	3,905	41,753
0	16,132	6,525	9,607	102,719
0	12,766	9,393	3,373	36,064
0	15,977	6,006	9,971	106,611
0	12,637	4,820	7,817	83,580
0	15,803	7,409	8,394	89,749
0	16,346	6,693	9,653	103,211
0	11,921	5,541	6,380	68,215
0	18,556	6,490	12,066	129,011
0	14,193	4,580	9,613	102,783
0	15,074	5,002	10,072	107,691
0	15,242	5,811	9,431	100,837
0	14,179	4,273	9,906	105,916
0	16,358	5,007	11,351	121,366
0	13,344	5,933	7,411	79,239
0	13,709	7,015	6,694	71,573
0	20,903	6,891	14,012	149,817
0	22,826	7,793	15,033	160,734
0	10,333	6,880	3,453	36,920
0	19,995	9,516	10,479	112,042
0	12,108	5,272	6,836	73,091
0	11,658	5,481	6,177	66,045
0	13,162	4,755	8,407	89,888
0	13,297	4,763	8,534	91,246
0	12,967	4,846	8,121	86,830
0	15,063	6,119	8,944	95,630
0	22,467	7,462	15,005	160,435
0	13,095	4,517	8,578	91,717
0	18,860	5,455	13,405	143,327
0	14,429	5,074	9,355	100,024
0	12,826	6,837	5,989	64,035
50	19,975	8,129	11,846	126,658
50	22,229	6,057	16,172	172,912
50	12,514	6,738	5,776	61,757
50	16,270	8,144	8,126	86,884
50	15,232	5,960	9,272	99,137
50	22,371	5,923	16,448	175,863
50	18,789	9,951	8,838	94,497
50	20,050	10,386	9,664	103,328
50	22,857	11,480	11,377	121,644
50	20,961	11,244	9,717	103,895
50	16,731	10,661	6,070	64,901
50	24,070	9,047	15,023	160,627
50	20,576	10,918	9,658	103,264
50	23,791	12,721	11,070	118,361
50	21,930	15,727	6,203	66,323
50	22,873	11,618	11,255	120,339
50	19,628	9,512	10,116	108,161
50	20,784	9,016	11,768	125,824
50	16,157	8,335	7,822	83,633
50	17,010	6,240	10,770	115,154
50	18,728	5,831	12,897	137,896
50	17,966	9,610	8,356	89,343
50	20,217	8,775	11,442	122,339
50	20,063	8,271	11,792	126,081
50	15,887	8,269	7,618	81,452
50	15,830	8,091	7,739	82,746
50	16,576	8,130	8,446	90,305
50	14,963	6,814	8,149	87,130
50	17,187	6,786	10,401	111,208
50	20,031	9,324	10,707	114,480
100	24,632	11,500	13,132	140,408
100	24,242	10,122	14,120	150,972
100	12,569	6,169	6,400	68,429

0	23,766	4,046	19,720	85,840
0	23,453	2,295	21,158	92,100
0	15,243	2,882	12,361	53,807
0	24,894	5,156	19,738	85,918
0	15,333	2,477	12,856	55,961
0	17,837	2,084	15,753	68,572
0	14,546	2,781	11,765	51,212
0	13,537	2,126	11,411	49,671
0	14,326	3,333	10,993	47,852
0	16,654	3,064	13,590	59,156
0	32,633	1,633	31,000	134,941
0	13,344	1,741	11,603	50,507
0	40,344	1,188	39,156	170,444
0	12,527	3,525	9,002	39,185
0	15,938	2,669	13,269	57,759
0	31,163	3,314	27,849	121,225
0	11,462	3,787	7,675	33,409
0	16,386	2,061	14,325	62,356
0	18,724	3,478	15,246	66,365
0	13,865	4,694	9,171	39,921
0	20,533	2,990	17,543	76,364
0	17,891	4,825	13,066	56,876
0	16,924	3,168	13,756	59,879
0	18,865	3,561	15,304	66,617
0	21,331	4,162	17,169	74,736
0	13,112	3,531	9,581	41,706
0	15,574	3,393	12,181	53,023
0	16,971	3,737	13,234	57,607
0	21,624	5,423	16,201	70,522
0	23,649	3,160	20,489	89,187
0	19,076	3,626	15,450	67,253
0	19,432	3,067	16,365	71,236
0	19,564	3,328	16,236	70,674
0	15,393	2,849	12,544	54,603
0	16,921	1,865	15,056	65,538
0	17,087	1,453	15,634	68,054
0	19,090	1,380	17,710	77,091
0	19,247	1,363	17,884	77,848
0	16,313	1,479	14,834	64,572
0	18,059	2,148	15,911	69,260
0	15,306	2,310	12,996	56,571
0	23,454	1,839	21,615	94,089
0	20,599	2,669	17,930	78,048
0	20,479	2,977	17,502	76,185
0	18,891	3,106	15,785	68,711
0	22,223	2,763	19,460	84,708
0	23,612	2,363	21,249	92,496
0	28,341	2,493	25,848	112,515
0	35,218	3,208	32,010	139,338
0	32,632	1,403	31,229	135,938
0	23,178	2,211	20,967	91,268
0	20,551	2,243	18,308	79,694
0	35,758	1,502	34,256	149,114
0	30,611	2,977	27,634	120,289
0	41,917	2,507	39,410	171,549
0	32,909	2,535	30,374	132,216
0	32,440	2,646	29,794	129,692
0	27,690	4,319	23,371	101,733
0	25,613	3,593	22,020	95,852
0	24,657	3,910	20,747	90,311
0	28,620	3,511	25,109	109,298
0	33,412	4,721	28,691	124,890
200	37,973	3,912	34,061	148,266
200	29,441	5,274	24,167	105,198
200	33,335	4,588	28,747	125,134
200	34,421	3,048	31,373	136,565
200	38,439	3,063	35,376	153,990
200	32,408	3,290	29,118	126,749
200	23,725	3,239	20,486	89,174
200	32,223	1,100	31,123	135,477
200	28,596	5,036	23,560	102,555

100	11,838	2,632	9,21	39,69
100	36,003	2,182	33,82	145,83
100	54,983	2,159	52,82	227,77
100	16,319	2,831	13,49	58,16
100	24,522	1,266	23,26	100,28
100	23,401	2,818	20,58	88,75
100	16,692	1,658	15,03	64,82
200	37,13	1,61	35,52	153,16
200	14,938	1,637	13,30	57,35
200	18,8	1,035	17,77	76,60
200	19,58	1,371	18,21	78,51
200	19,592	1,143	18,45	79,55
200	8,563	1,207	7,36	31,72
200	41,196	1,198	40,00	172,46
200	20,208	0,994	19,21	82,85
200	8,815	0,982	7,83	33,77
200	34,694	1,125	33,57	144,74
200	16,69	1,201	15,49	66,79
200	14,896	1,295	13,60	58,65
200	32,397	3,587	28,81	124,22
200	7,143	2,002	5,14	22,17
200	14,159	2,007	12,15	52,40
200	13,102	1,479	11,62	50,12
200	39,564	2,005	37,56	161,95
200	39,261	1,971	37,29	160,79
200	32,422	1,9	30,52	131,61
200	14,192	1,715	12,48	53,80
200	14,612	1,6	13,01	56,11
200	16,561	1,86	14,70	63,39
200	15,721	1,301	14,42	62,18
200	10,472	1,325	9,15	39,44
200	10,637	1,06	9,58	41,29
200	15,25	1,883	13,37	57,64
200	11,139	1,42	9,72	41,91
200	20,306	1,151	19,16	82,59
200	17,937	1,147	16,79	72,40
200	20,75	1,499	19,25	83,01
200	13,691	1,179	12,51	53,95
200	9,818	1,112	8,71	37,54
200	12,942	1,366	11,58	49,91
200	9,381	1,309	8,07	34,81
200	12,249	1,139	11,11	47,90
200	11,554	1,135	10,42	44,92
200	36,305	1,091	35,21	151,84
200	15,141	1,116	14,03	60,47
200	9,771	1,038	8,73	37,66
200	9,841	1,3	8,54	36,83
200	35,953	1,467	34,49	148,70
200	18,559	1,574	16,99	73,24
200	31,068	1,797	29,27	126,21
200	19,922	1,87	18,05	77,84
200	33,338	1,196	32,14	138,59
200	16,728	1,081	15,65	67,47
200	19,583	1,599	17,98	77,54
200	10,339	1,602	8,74	37,67
200	13,523	1,506	12,02	51,82
200	30,614	1,647	28,97	124,90
200	8,844	1,016	7,83	33,75
200	11,852	1,437	10,42	44,91
200	26,417	1,641	24,78	106,83
200	17,679	1,697	15,98	68,91
200	8,446	2,021	6,43	27,70
200	13,199	1,692	11,51	49,62
200	4,869	1,029	3,84	16,56
400	18,264	1,242	17,02	73,40
400	42,885	1,434	41,45	178,73
400	23,659	1,416	22,24	95,91
400	13,109	1,267	11,84	51,06
400	9,901	1,122	8,78	37,85
400	12,048	1,152	10,90	46,98
400	22,81	1,155	21,66	93,37

100	18,267	6,186	12,081	129,171
100	19,309	6,727	12,582	134,528
100	17,653	8,032	9,621	102,868
100	21,104	6,551	14,553	155,602
100	27,445	8,701	18,744	200,412
100	24,988	10,484	14,504	155,078
100	20,987	8,112	12,875	137,660
100	31,779	14,709	17,070	182,514
100	18,785	7,395	11,390	121,783
100	18,219	9,449	8,770	93,769
100	13,884	6,207	7,677	82,083
100	20,596	5,829	14,767	157,890
100	18,379	5,520	12,859	137,489
200	14,188	7,255	6,933	74,128
200	15,119	8,288	6,831	73,038
200	14,554	8,044	6,510	69,605
200	9,931	4,866	5,065	54,155
200	10,566	5,362	5,204	55,642
200	10,935	5,279	5,656	60,474
200	9,625	4,925	4,700	50,253
200	11,186	4,580	6,606	70,632
200	9,348	4,933	4,415	47,206
400	14,411	5,683	8,728	93,320
400	9,928	5,805	4,123	44,083
400	11,566	3,760	7,806	83,462
400	16,131	5,860	10,271	109,818
400	20,192	8,292	11,900	127,236
400	13,129	7,430	5,699	60,934
400	11,262	5,664	5,598	59,854
400	19,138	7,942	11,196	119,708
400	15,743	6,961	8,782	93,898
400	15,252	7,262	7,990	85,430
400	22,410	7,795	14,615	156,265
400	13,821	7,605	6,216	66,462
400	18,386	6,299	12,087	129,235
400	13,338	6,528	6,810	72,813
400	13,912	5,145	8,767	93,737

NAcGal	RI	BKG	RI-BKG	RI%
0	22,382	8,432	13,950	149,154
0	21,508	8,015	13,493	144,268
0	23,600	6,682	16,918	180,889
0	20,449	9,659	10,790	115,367
0	9,615	6,040	3,575	38,224
0	9,709	5,804	3,905	41,753
0	16,132	6,525	9,607	102,719
0	12,766	9,393	3,373	36,064
0	15,977	6,006	9,971	106,611
0	12,637	4,820	7,817	83,580
0	15,803	7,409	8,394	89,749
0	16,346	6,693	9,653	103,211
0	11,921	5,541	6,380	68,215
0	18,556	6,490	12,066	129,011
0	14,193	4,580	9,613	102,783
0	15,074	5,002	10,072	107,691
0	15,242	5,811	9,431	100,837
0	14,179	4,273	9,906	105,916
0	16,358	5,007	11,351	121,366
0	13,344	5,933	7,411	79,239
0	13,709	7,015	6,694	71,573
0	20,903	6,891	14,012	149,817
0	22,826	7,793	15,033	160,734
0	10,333	6,880	3,453	36,920
0	19,995	9,516	10,479	112,042
0	12,108	5,272	6,836	73,091
0	11,658	5,481	6,177	66,045
0	13,162	4,755	8,407	89,888
0	13,297	4,763	8,534	91,246
0	12,967	4,846	8,121	86,830
0	15,063	6,119	8,944	95,630
0	22,467	7,462	15,005	160,435

200	23,092	3,878	19,214	83,637
200	21,109	5,694	15,415	67,101
200	28,518	5,044	23,474	102,181
200	41,406	6,204	35,202	153,232
200	30,461	4,874	25,587	111,379
200	21,751	4,633	17,118	74,514
200	30,631	6,382	24,249	105,555
200	15,134	5,424	9,710	42,267
200	22,549	5,782	16,767	72,986
200	27,482	2,173	25,309	110,169
200	31,675	4,290	27,385	119,205
200	20,345	5,044	15,301	66,604
200	19,243	4,045	15,198	66,156
200	14,319	5,266	9,053	39,407
200	13,624	4,888	8,736	38,027
200	24,340	3,877	20,463	89,074
200	25,504	5,695	19,809	86,227
200	26,195	3,201	22,994	100,092
200	22,591	3,050	19,541	85,061
200	28,181	3,896	24,285	105,711
200	26,061	6,498	19,563	85,157
200	26,685	5,111	21,574	93,910
200	19,111	3,971	15,140	65,904
200	28,078	3,917	24,161	105,171
200	15,814	2,954	12,860	55,979
200	21,626	4,406	17,220	74,958
200	18,999	4,853	14,146	61,577
200	27,217	3,397	23,820	103,687
200	20,179	1,961	18,218	79,302
200	26,354	3,882	22,472	97,819
200	14,966	4,029	10,937	47,608
200	17,528	2,959	14,569	63,418
200	18,817	3,966	14,851	64,646
200	16,543	3,640	12,903	56,166
200	14,555	3,745	10,810	47,055
200	23,763	3,243	20,520	89,322
200	21,714	3,638	18,076	78,684
200	15,718	3,453	12,265	53,389
200	17,108	3,740	13,368	58,190
200	12,480	3,378	9,102	39,620
200	20,131	4,383	15,748	68,550
200	22,674	4,317	18,357	79,907
200	13,125	5,464	7,661	33,348
200	19,224	4,577	14,647	63,758
200	18,624	4,489	14,135	61,529
200	34,321	6,763	27,558	119,958
200	26,472	5,673	20,799	90,537
200	34,236	4,817	29,419	128,059
200	27,612	6,775	20,837	90,702
200	21,492	4,336	17,156	74,679
200	24,461	6,335	18,126	78,901
200	31,946	8,703	23,243	101,175
200	18,476	7,980	10,496	45,688
200	18,697	6,839	11,858	51,617
200	28,356	6,178	22,178	96,540
400	22,265	6,926	15,339	66,770
400	21,776	6,624	15,152	65,956
400	20,843	3,533	17,310	75,349
400	26,616	5,526	21,090	91,804
400	24,773	4,868	19,905	86,645
400	25,523	5,264	20,259	88,186
400	29,491	6,718	22,773	99,130
400	26,674	4,174	22,500	97,941
400	19,762	3,688	16,074	69,969
400	19,452	5,781	13,671	59,509
400	27,721	5,250	22,471	97,815
400	26,975	4,494	22,481	97,859
400	18,571	5,174	13,397	58,316
400	21,701	7,375	14,326	62,360
400	30,602	6,761	23,841	103,779
400	21,742	5,643	16,099	70,078

400	14,547	1,728	12,82	55,27
400	22,044	1,593	20,45	88,18
400	42,317	1,04	41,28	177,98
400	5,986	0,845	5,14	22,17
400	12,443	1,216	11,23	48,41
400	12,512	1,295	11,22	48,37
400	10,288	1,406	8,88	38,30
400	16,145	2,015	14,13	60,93
400	22,119	2,624	19,50	84,06
400	50,298	2,036	48,26	208,10
400	49,336	1,97	47,37	204,23
400	26,113	2,001	24,11	103,97
400	12,801	2,294	10,51	45,30
400	24,378	2,67	21,71	93,60
400	22,245	2,348	19,90	85,79
400	15,005	2,648	12,36	53,28
400	23,676	2,949	20,73	89,37
400	42,262	2,559	39,70	171,19
400	11,726	2,765	8,96	38,64
400	46,18	2,299	43,88	189,21
400	22,566	2,404	20,16	86,93
400	31,884	2,589	29,30	126,31
400	13,724	2,737	10,99	47,37
400	8,746	2,571	6,18	26,63
400	14,616	1,784	12,83	55,33
400	38,426	1,979	36,45	157,15
400	12,456	2,81	9,65	41,59
400	32,883	3,108	29,78	128,38
400	8,573	2,73	5,84	25,19
400	7,598	2,173	5,43	23,39
400	11,158	2,411	8,75	37,72
400	11,945	2,069	9,88	42,58
400	16,136	2,417	13,72	59,15
400	13,961	1,515	12,45	53,66
400	10,356	1,606	8,75	37,73
400	11,122	1,406	9,72	41,89
400	17,153	1,888	15,27	65,82
400	51,61	1,961	49,65	214,08
400	18,405	2,16	16,25	70,05
400	31,475	2,343	29,13	125,61
400	32,755	2,342	30,41	131,14
400	31,201	2,002	29,20	125,90
400	56,406	2,022	54,38	234,49
400	30,363	1,814	28,55	123,10
400	27,38	2,323	25,06	108,04
400	28,05	1,914	26,14	112,69
400	49,76	1,747	48,01	207,02
400	21,207	2,057	19,15	82,57
400	14,302	1,901	12,40	53,47
400	18,593	1,917	16,68	71,90
400	54,728	1,065	53,66	231,39
400	14,214	1,795	12,42	53,55
400	32,182	1,704	30,48	131,42
400	46,297	1,369	44,93	193,72
400	30,49	1,173	29,32	126,41
400	18,219	2,153	16,07	69,27
400	23,763	1,826	21,94	94,59
400	8,683	1,67	7,01	30,24
400	9,485	1,797	7,69	33,15
400	12,383	1,626	10,76	46,38
400	20,972	2,041	18,93	81,63
800	16,76	1,252	15,51	66,87
800	10,111	1,322	8,79	37,90
800	21,666	1,113	20,55	88,62
800	14,522	1,68	12,84	55,37
800	16,691	1,565	15,13	65,22
800	32,728	0,74	31,99	137,93
800	28,305	1,091	27,21	117,34
800	8,962	0,996	7,97	34,35
800	17,025	1,404	15,62	67,35
800	27,389	1,562	25,83	111,36

0	13,095	4,517	8,578	91,717
0	18,860	5,455	13,405	143,327
0	14,429	5,074	9,355	100,024
0	12,826	6,837	5,989	64,035
50	16,694	6,208	10,486	112,117
50	11,982	6,829	5,153	55,096
50	14,467	4,758	9,709	103,809
50	15,347	7,772	7,575	80,992
50	18,191	6,438	11,753	125,664
50	19,966	6,784	13,182	140,943
50	17,284	8,646	8,638	92,358
50	11,180	5,504	5,676	60,688
50	12,574	5,287	7,287	77,913
50	15,179	4,695	10,484	112,096
50	11,796	4,676	7,120	76,128
50	11,175	4,991	6,184	66,120
50	18,367	9,156	9,211	98,485
50	13,373	6,700	6,673	71,348
50	10,276	4,171	6,105	65,275
50	13,123	5,250	7,873	84,179
50	14,729	9,193	5,536	59,191
50	15,861	6,127	9,734	104,077
50	11,983	5,836	6,147	65,724
50	18,306	8,069	10,237	109,455
50	17,065	4,690	12,375	132,314
50	14,428	8,090	6,338	67,766
50	14,982	4,838	10,144	108,460
50	17,887	7,216	10,671	114,095
100	9,941	4,923	5,018	53,653
100	14,936	5,479	9,457	101,115
100	11,350	4,384	6,966	74,481
100	11,807	4,658	7,149	76,438
100	13,141	3,300	9,841	105,221
100	14,013	4,136	9,877	105,606
100	15,054	6,327	8,727	93,310
100	11,835	3,254	8,581	91,749
100	10,660	2,784	7,876	84,211
100	13,835	3,847	9,988	106,792
100	12,132	2,573	9,559	102,206
100	10,542	2,473	8,069	86,274
100	12,746	7,606	5,140	54,957
200	22,015	8,864	13,151	140,611
200	14,918	5,402	9,516	101,746
200	21,004	9,694	11,310	120,927
200	17,844	7,697	10,147	108,492
200	14,419	7,453	6,966	74,481
200	11,021	4,734	6,287	67,221
200	11,305	6,645	4,660	49,825
200	17,974	8,477	9,497	101,543
200	14,045	5,223	8,822	94,325
200	23,618	5,774	17,844	190,789
200	18,297	4,417	13,880	148,406
200	18,280	4,244	14,036	150,074
200	19,120	4,190	14,930	159,633
200	24,302	3,697	20,605	220,310
200	17,307	9,711	7,596	81,217
400	12,286	5,264	7,022	75,080
400	14,055	3,904	10,151	108,535
400	11,686	4,827	6,859	73,337
400	12,233	5,548	6,685	71,477
400	13,533	4,952	8,581	91,749
400	15,556	6,039	9,517	101,756
400	10,572	5,078	5,494	58,742
400	16,097	7,093	9,004	96,271
400	13,598	6,406	7,192	76,897
400	11,786	4,788	6,998	74,823
400	10,571	4,415	6,156	65,820
400	10,023	4,181	5,842	62,463
400	9,697	3,611	6,086	65,072
400	9,869	3,308	6,561	70,151
400	16,707	6,844	9,863	105,456

400	25,101	7,860	17,241	75,049
400	26,701	3,621	23,080	100,466
400	20,139	4,948	15,191	66,126
400	21,176	3,305	17,871	77,791
400	17,955	3,681	14,274	62,134
400	34,978	5,352	29,626	128,960
400	17,695	5,639	12,056	52,479
400	18,327	5,088	13,239	57,629
400	35,197	1,437	33,760	146,955
400	32,624	5,761	26,863	116,933
400	24,713	4,686	20,027	87,176
400	16,054	3,585	12,469	54,277
400	22,228	3,891	18,337	79,820
400	29,946	3,103	26,843	116,846
400	27,071	4,950	22,121	96,291
400	27,277	4,701	22,576	98,272
400	21,021	4,399	16,622	72,355
400	21,622	4,673	16,949	73,778
400	19,866	3,247	16,619	72,342
400	24,897	5,290	19,607	85,348
400	17,515	6,180	11,335	49,341
400	25,995	6,408	19,587	85,261
400	25,249	4,478	20,771	90,415
400	16,846	5,387	11,459	49,880
400	28,705	5,754	22,951	99,904
400	15,761	5,867	9,894	43,068
400	23,717	4,290	19,427	84,565
400	33,033	7,071	25,962	113,011
400	26,153	6,913	19,240	83,751
400	33,708	10,777	22,931	99,817
400	32,845	8,431	24,414	106,273
400	28,276	5,609	22,667	98,668
400	30,733	4,423	26,310	114,526
400	27,766	3,355	24,411	106,260
400	25,998	8,524	17,474	76,063
400	20,333	6,672	13,661	59,466
800	22,605	2,445	20,160	87,755
800	16,507	1,630	14,877	64,759
800	22,015	2,888	19,127	83,259
800	21,152	3,182	17,970	78,222
800	18,708	2,971	15,737	68,502
800	22,605	2,769	19,836	86,345
800	19,318	2,933	16,385	71,323
800	21,581	3,344	18,237	79,385
800	20,220	3,077	17,143	74,622
800	21,445	1,848	19,597	85,305
800	10,651	3,256	7,395	32,190
800	12,278	3,817	8,461	36,830
800	9,751	3,022	6,729	29,291
800	10,072	2,630	7,442	32,395
800	12,751	1,760	10,991	47,843
800	10,072	1,936	8,136	35,416
800	9,817	2,002	7,815	34,018
800	11,596	1,392	10,204	44,417
800	13,087	1,543	11,544	50,250
800	10,877	2,154	8,723	37,971
800	17,440	2,122	15,318	66,678
800	23,208	2,355	20,853	90,772
800	18,239	2,301	15,938	69,377
800	17,732	2,265	15,467	67,327
800	12,184	3,474	8,710	37,914
800	21,343	3,063	18,280	79,572
800	15,126	3,305	11,821	51,456
800	18,864	3,572	15,292	66,565
800	23,168	4,282	18,886	82,210
800	20,699	5,233	15,466	67,323
800	19,827	2,896	16,931	73,700
800	18,208	3,199	15,009	65,333
800	18,434	4,405	14,029	61,067
800	17,244	3,928	13,316	57,964
800	20,500	3,980	16,520	71,911

800	9,484	1,163	8,32	35,88
800	18,05	2,851	15,20	65,54
800	44,958	4,504	40,45	174,43
800	12,4	4,495	7,91	34,08
800	38,767	3,652	35,12	151,41
800	20,868	3,635	17,23	74,31
800	31,144	3,655	27,49	118,53
800	49,117	3,239	45,88	197,82
800	27,047	3,358	23,69	102,14
800	14,978	3,577	11,40	49,16
800	33,125	3,209	29,92	128,99
800	20,018	3,276	16,74	72,19
800	34,018	2,736	31,28	134,88
800	25,523	3,199	22,32	96,26
800	19,854	3,335	16,52	71,23
800	28,003	2,885	25,12	108,30
800	16,375	2,418	13,96	60,18
800	10,05	1,608	8,44	36,40
800	13,099	3,094	10,01	43,14
800	5,026	1,929	3,10	13,35
800	42,86	1,844	41,02	176,85
800	11,592	2,899	8,69	37,48
800	21,87	3,386	18,48	79,70
800	25,325	3,75	21,58	93,03
800	26,073	2,236	23,84	102,78
800	22,979	4,13	18,85	81,27
800	15,5	3,075	12,43	53,57
800	12,867	1,773	11,09	47,84
800	9,253	2,158	7,10	30,59
800	13,985	2,223	11,76	50,72
800	17,194	1,04	16,15	69,65
800	13,785	2,705	11,08	47,77
800	6,23	1,513	4,72	20,34

Fru	IM CA	IM BKG	CA-BKG	RI%
0	17,941	1,18	16,76	72,27
0	14,896	1,56	13,34	57,50
0	20,267	1,105	19,16	82,62
0	11,695	1,155	10,54	45,45
0	12,994	1,099	11,90	51,29
0	18,422	1,117	17,31	74,62
0	25,79	1,451	24,34	104,95
0	15,103	1,297	13,81	59,53
0	34,569	1,484	33,09	142,66
0	14,138	0,489	13,65	58,85
0	15,875	1,945	13,93	60,06
0	20,7	1,872	18,83	81,18
0	11,954	1,578	10,38	44,74
0	20,754	1,861	18,89	81,46
0	50,282	2,711	47,57	205,12
0	33,745	1,545	32,20	138,84
0	13,889	0,578	13,31	57,39
0	22,582	1,208	21,37	92,16
0	14,618	1,033	13,59	58,58
0	11,554	1,565	9,99	43,07
0	11,569	1,34	10,23	44,11
0	23,41	1,023	22,39	96,53
0	33,797	0,963	32,83	141,57
0	26,289	0,954	25,34	109,24
0	10,437	1,078	9,36	40,35
0	21,082	0,836	20,25	87,30
0	8,763	1,164	7,60	32,77
0	37,612	1,234	36,38	156,86
0	16,769	1,149	15,62	67,35
0	29,258	1,343	27,92	120,36
0	11,666	1,115	10,55	45,49
0	25,676	0,895	24,78	106,85
0	39,99	1,282	38,71	166,90
0	18,783	1,742	17,04	73,48
0	25,57	2,058	23,51	101,38
0	12,614	2,737	9,88	42,59

400	14,301	5,732	8,569	91,620
400	10,358	3,086	7,272	77,753
400	9,802	6,226	3,576	38,235
400	11,287	3,526	7,761	82,981
400	9,457	4,541	4,916	52,562
400	16,649	8,353	8,296	88,701
400	16,258	7,501	8,757	93,630
400	12,723	4,732	7,991	85,440
400	14,878	4,565	10,313	110,267
400	10,947	4,331	6,616	70,739
400	13,434	4,187	9,247	98,870
400	9,665	5,112	4,553	48,681
400	14,193	5,065	9,128	97,597
400	15,829	7,501	8,328	89,044

800	13,296	4,985	8,311	36,177
800	16,757	3,792	12,965	56,436
800	18,277	2,308	15,969	69,512
800	21,241	3,585	17,656	76,856
800	9,002	3,630	5,372	23,384
800	16,095	5,047	11,048	48,091
800	19,391	4,165	15,226	66,278
800	23,096	6,021	17,075	74,326
800	28,171	5,703	22,468	97,802
800	19,550	6,087	13,463	58,604
800	16,017	7,035	8,982	39,098
800	16,343	5,993	10,350	45,053
800	30,527	5,933	24,594	107,056
800	12,302	5,934	6,368	27,720
800	18,663	4,516	14,147	61,581
800	14,313	5,313	9,000	39,176
800	10,916	5,622	5,294	23,044
800	9,989	3,698	6,291	27,384
800	15,515	4,277	11,238	48,918
800	16,104	3,945	12,159	52,927
800	12,787	4,966	7,821	34,044

NAcGal	RI	BKG	RI-BKG	RI%
0	42,915	4,513	38,402	167,162
0	39,957	2,552	37,405	162,822
0	36,092	5,212	30,880	134,419
0	27,120	6,067	21,053	91,643
0	28,937	4,353	24,584	107,013
0	32,143	3,571	28,572	124,372
0	32,805	3,783	29,022	126,331
0	53,870	5,367	48,503	211,131
0	40,904	6,247	34,657	150,860
0	41,614	5,428	36,186	157,516
0	43,696	7,158	36,538	159,048
0	36,065	9,731	26,334	114,630
0	37,297	6,909	30,388	132,277
0	46,197	7,851	38,346	166,918
0	35,676	8,219	27,457	119,519
0	47,154	9,385	37,769	164,406
0	45,699	8,205	37,494	163,209
0	37,990	7,530	30,460	132,591
0	38,982	9,165	29,817	129,792
0	44,030	10,100	33,930	147,695
0	42,055	10,479	31,576	137,449
0	53,660	8,190	45,470	197,928
0	51,363	6,648	44,715	194,642
0	30,309	4,286	26,023	113,277
0	23,766	4,046	19,720	85,840
0	23,453	2,295	21,158	92,100
0	15,243	2,882	12,361	53,807
0	24,894	5,156	19,738	85,918
0	15,333	2,477	12,856	55,961
0	17,837	2,084	15,753	68,572
0	14,546	2,781	11,765	51,212
0	13,537	2,126	11,411	49,671
0	14,326	3,333	10,993	47,852
0	16,654	3,064	13,590	59,156
0	32,633	1,633	31,000	134,941
0	13,344	1,741	11,603	50,507
0	40,344	1,188	39,156	170,444
0	12,527	3,525	9,002	39,185
0	15,938	2,669	13,269	57,759
0	31,163	3,314	27,849	121,225
0	11,462	3,787	7,675	33,409
0	16,386	2,061	14,325	62,356
0	18,724	3,478	15,246	66,365
0	13,865	4,694	9,171	39,921
0	20,533	2,990	17,543	76,364
0	17,891	4,825	13,066	56,876
0	16,924	3,168	13,756	59,879
0	18,865	3,561	15,304	66,617

0	21,087	2,11	18,98	81,83
0	14,029	2,346	11,68	50,37
0	32,361	2,698	29,66	127,90
0	56,725	2,782	53,94	232,59
0	33,573	3,201	30,37	130,96
0	33,655	2,61	31,05	133,86
0	29,996	2,314	27,68	119,36
0	11,177	3,027	8,15	35,14
0	13,892	2,485	11,41	49,18
0	25,12	1,968	23,15	99,83
0	46,437	3,573	42,86	184,82
0	20,731	3,009	17,72	76,41
0	9,836	3,021	6,82	29,39
0	16,517	3,006	13,51	58,26
0	16,476	3,002	13,47	58,10
0	23,276	1,756	21,52	92,79
0	24,742	3,614	21,13	91,10
0	36,92	3,432	33,49	144,39
0	41,23	2,566	38,66	166,71
0	63,491	6,917	56,57	243,94
0	59,866	5,125	54,74	236,03
0	52,024	5,227	46,80	201,78
0	41,031	5,574	35,46	152,88
0	55,864	3,74	52,12	224,75
0	28,138	8,195	19,94	85,99
0	11,069	2,234	8,84	38,09
0	35,684	2,027	33,66	145,12
0	17,196	2,599	14,60	62,94
50	34,647	1,773	32,87	141,75
50	41,209	1,446	39,76	171,45
50	14,985	1,635	13,35	57,56
50	10,998	1,307	9,69	41,79
50	30,992	1,35	29,64	127,81
50	15,083	1,072	14,01	60,41
50	19,758	1,442	18,32	78,98
50	8,63	1,399	7,23	31,18
50	16,315	1,473	14,84	64,00
50	11,755	1,124	10,63	45,84
50	11,849	1,112	10,74	46,30
50	6,263	0,916	5,35	23,06
50	6,511	1,117	5,39	23,26
50	16,901	1,349	15,55	67,06
50	7,501	1,21	6,29	27,13
50	8,028	1,66	6,37	27,46
50	34,68	2,081	32,60	140,56
50	19,723	1,994	17,73	76,44
50	31,979	2,01	29,97	129,22
50	12,404	1,593	10,81	46,62
50	20,558	1,516	19,04	82,11
50	49,049	1,192	47,86	206,35
50	24,657	2,097	22,56	97,27
50	24,314	1,539	22,78	98,20
50	22,499	0,932	21,57	92,99
50	17,644	1,048	16,60	71,56
50	22,918	1,642	21,28	91,74
50	18,303	1,432	16,87	72,74
50	32,762	1,552	31,21	134,57
50	15,679	1,112	14,57	62,81
50	11,272	1,258	10,01	43,18
50	16,542	1,224	15,32	66,05
50	9,109	1,155	7,95	34,30
50	24,027	2,387	21,64	93,31
50	40,64	1,735	38,91	167,75
50	45,789	2,128	43,66	188,26
50	16,35	1,854	14,50	62,50
50	17,139	1,635	15,50	66,85
50	35,435	1,254	34,18	147,38
50	11,197	2,076	9,12	39,33
50	16,571	1,653	14,92	64,32
50	43,624	1,832	41,79	180,20
50	9,976	1,441	8,54	36,80

0	21,331	4,162	17,169	74,736
0	13,112	3,531	9,581	41,706
0	15,574	3,393	12,181	53,023
0	16,971	3,737	13,234	57,607
0	21,624	5,423	16,201	70,522
0	23,649	3,160	20,489	89,187
0	19,076	3,626	15,450	67,253
0	19,432	3,067	16,365	71,236
0	19,564	3,328	16,236	70,674
0	15,393	2,849	12,544	54,603
0	16,921	1,865	15,056	65,538
0	17,087	1,453	15,634	68,054
0	19,090	1,380	17,710	77,091
0	19,247	1,363	17,884	77,848
0	16,313	1,479	14,834	64,572
0	18,059	2,148	15,911	69,260
0	15,306	2,310	12,996	56,571
0	23,454	1,839	21,615	94,089
0	20,599	2,669	17,930	78,048
0	20,479	2,977	17,502	76,185
0	18,891	3,106	15,785	68,711
0	22,223	2,763	19,460	84,708
0	23,612	2,363	21,249	92,496
0	28,341	2,493	25,848	112,515
0	35,218	3,208	32,010	139,338
0	32,632	1,403	31,229	135,938
0	23,178	2,211	20,967	91,268
0	20,551	2,243	18,308	79,694
0	35,758	1,502	34,256	149,114
0	30,611	2,977	27,634	120,289
0	41,917	2,507	39,410	171,549
0	32,909	2,535	30,374	132,216
0	32,440	2,646	29,794	129,692
0	27,690	4,319	23,371	101,733
0	25,613	3,593	22,020	95,852
0	24,657	3,910	20,747	90,311
0	28,620	3,511	25,109	109,298
0	33,412	4,721	28,691	124,890
200	44,671	3,638	41,033	178,614
200	32,260	6,467	25,793	112,277
200	9,845	2,600	7,245	31,537
200	30,531	4,106	26,425	115,026
200	37,267	5,606	31,661	137,819
200	31,259	6,191	25,068	109,118
200	32,043	4,660	27,383	119,198
200	30,516	7,247	23,269	101,291
200	16,323	5,076	11,247	48,956
200	32,449	6,036	26,413	114,972
200	15,557	5,809	9,748	42,432
200	30,989	4,990	25,999	113,170
200	29,084	6,689	22,395	97,485
200	24,795	4,516	20,279	88,272
200	31,510	7,453	24,057	104,721
200	34,256	7,290	26,966	117,383
200	33,119	7,661	25,458	110,819
200	27,884	8,867	19,017	82,779
200	43,052	7,583	35,469	154,396
200	40,556	7,092	33,464	145,668
200	42,192	9,031	33,161	144,349
200	26,261	10,979	15,282	66,523
200	26,391	8,845	17,546	76,377
200	43,966	6,876	37,090	161,449
200	28,603	6,622	21,981	95,680
200	43,725	6,832	36,893	160,595
200	21,016	4,013	17,003	74,015
200	13,397	3,065	10,332	44,975
200	15,077	4,812	10,265	44,684
200	25,744	4,341	21,403	93,165
200	25,911	4,277	21,634	94,174
200	14,202	3,779	10,423	45,369
200	7,864	4,071	3,793	16,509

50	22,131	1,902	20,23	87,22
50	8,749	1,786	6,96	30,02
50	28,845	1,182	27,66	119,28
50	46,852	1,605	45,25	195,10
50	40,415	2,231	38,18	164,64
50	49,292	2,313	46,98	202,56
50	58,277	1,868	56,41	243,23
50	59,296	1,875	57,42	247,59
50	39,535	2,066	37,47	161,56
50	39,71	4,426	35,28	152,14
50	12,04	1,271	10,77	46,43
50	17,666	1,344	16,32	70,38
50	30,268	1,689	28,58	123,23
50	14,57	1,662	12,91	55,66
50	9,24	1,046	8,19	35,33
50	40,717	1,203	39,51	170,38
100	11,568	1,148	10,42	44,93
100	20,507	0,99	19,52	84,15
100	18,936	1,228	17,71	76,35
100	17,526	1,227	16,30	70,28
100	12,45	1,424	11,03	47,54
100	23,351	2,483	20,87	89,98
100	17,602	1,066	16,54	71,30
100	9,754	1,56	8,19	35,33
100	7,743	1,299	6,44	27,79
100	8,492	0,5	7,99	34,46
100	19,395	0,842	18,55	80,00
100	17,644	1,093	16,55	71,36
100	17,008	1,924	15,08	65,04
100	30,959	4,121	26,84	115,72
100	31,229	2,669	28,56	123,15
100	20,008	1,989	18,02	77,69
100	31,457	2,549	28,91	124,65
100	33,626	2,543	31,08	134,02
100	24,81	1,295	23,52	101,39
100	23,749	1,824	21,93	94,54
100	21,501	2,125	19,38	83,55
100	33,213	1,361	31,85	137,34
100	27,849	2,402	25,45	109,72
100	33,635	2,128	31,51	135,85
100	26,527	2,027	24,50	105,64
100	20,616	1,634	18,98	81,85
100	40,529	1,683	38,85	167,50
100	40,637	1,902	38,74	167,02
100	21,907	1,985	19,92	85,90
100	15,844	2,335	13,51	58,25
100	37,714	2,083	35,63	153,63
100	28,402	2,327	26,08	112,43
100	7,877	2,139	5,74	24,74
100	24,643	3,314	21,33	91,97
100	11,84	1,361	10,48	45,18
100	9,155	0,974	8,18	35,27
100	48,605	5,386	43,22	186,35
100	33,419	8,117	25,30	109,10
100	17,745	3,13	14,62	63,02
100	21,259	3,238	18,02	77,70
100	20,073	2,273	17,80	76,75
100	24,236	3,31	20,93	90,23
100	10,282	2,898	7,38	31,84
100	12,622	2,4	10,22	44,08
100	30,504	1,632	28,87	124,49
100	22,69	2,031	20,66	89,08
100	9,663	2,62	7,04	30,37
100	20,617	1,473	19,14	82,55
100	19,658	1,497	18,16	78,31
100	18,423	1,472	16,95	73,09
100	19,407	1,292	18,12	78,11
100	27,576	2,921	24,66	106,31
100	7,649	1,225	6,42	27,70
100	8,881	1,292	7,59	32,72
100	6,655	1,421	5,23	22,57

200	23,428	4,677	18,751	81,621
200	15,065	4,463	10,602	46,150
200	25,340	3,955	21,385	93,087
200	18,065	4,014	14,051	61,162
200	16,321	4,546	11,775	51,257
200	24,285	5,216	19,069	83,008
200	32,044	5,376	26,668	116,085
200	27,273	6,738	20,535	89,390
200	37,122	4,644	32,478	141,374
200	25,236	4,507	20,729	90,232
200	7,058	4,790	2,268	9,875
200	11,095	6,495	4,600	20,022
200	22,515	5,159	17,356	75,548
200	14,064	5,029	9,035	39,330
200	6,146	4,756	1,390	6,051
200	19,837	6,161	13,676	59,530
200	16,077	6,151	9,926	43,206
200	12,009	4,257	7,752	33,746
200	24,073	3,009	21,064	91,692
200	11,726	4,183	7,543	32,832
200	26,952	4,371	22,581	98,293
200	23,749	4,699	19,050	82,922
200	11,591	5,692	5,899	25,677
200	35,576	2,783	32,793	142,747
200	25,667	2,962	22,705	98,834
200	30,961	7,271	23,690	103,122
200	13,413	6,215	7,198	31,333
200	38,693	7,337	31,356	136,492
200	21,705	7,015	14,690	63,944
200	46,858	7,631	39,227	170,754
200	24,719	8,363	16,356	71,196
200	40,891	9,782	31,109	135,414
200	20,574	3,262	17,312	75,360
200	36,852	6,327	30,525	132,876
200	35,963	6,746	29,217	127,181
200	43,906	9,931	33,975	147,893
200	17,590	5,742	11,848	51,574
200	41,726	8,599	33,127	144,200
200	35,131	7,051	28,080	122,232
200	34,571	7,143	27,428	119,393
200	51,670	7,760	43,910	191,138
200	21,836	2,817	19,019	82,790
200	28,442	6,528	21,914	95,392
200	41,701	7,256	34,445	149,936
400	43,847	3,679	40,168	174,851
400	30,978	4,295	26,683	116,152
400	20,201	5,012	15,189	66,117
400	36,845	4,985	31,860	138,683
400	12,013	5,482	6,531	28,431
400	35,593	3,093	32,500	141,470
400	32,759	4,679	28,080	122,231
400	10,301	5,592	4,709	20,497
400	29,580	4,060	25,520	111,088
400	2,577	4,815	0,000	0,000
400	36,725	4,376	32,349	140,814
400	25,912	5,030	20,882	90,898
400	40,328	5,315	35,013	152,410
400	27,626	5,350	22,276	96,966
400	36,763	4,741	32,022	139,390
400	23,216	5,568	17,648	76,821
400	26,848	6,691	20,157	87,741
400	25,935	6,777	19,158	83,393
400	28,874	5,043	23,831	103,733
400	13,570	5,417	8,153	35,488
400	17,404	2,796	14,608	63,589
400	30,433	3,871	26,562	115,621
400	24,102	4,821	19,281	83,928
400	12,769	4,991	7,778	33,857
400	24,941	4,429	20,512	89,287
400	14,445	4,341	10,104	43,980
400	10,391	5,156	5,235	22,786

100	28,555	3,248	25,31	109,12
200	26,914	1,817	25,10	108,21
200	60,056	2,847	57,21	246,67
200	63,843	2,437	61,41	264,77
200	22,419	2,424	20,00	86,21
200	26,286	2,663	23,62	101,86
200	68,753	2,39	66,36	286,15
200	21,657	2,462	19,20	82,77
200	38,056	2,55	35,51	153,10
200	40,635	1,754	38,88	167,65
200	11,352	1,832	9,52	41,05
200	20,686	2,033	18,65	80,43
200	15,076	1,058	14,02	60,44
200	21,324	1,461	19,86	85,65
200	36,194	1,844	34,35	148,11
200	16,161	1,828	14,33	61,80
200	26,784	2,706	24,08	103,82
200	17,548	1,838	15,71	67,74
200	15,841	1,658	14,18	61,15
200	12,111	1,672	10,44	45,01
200	13,967	1,69	12,28	52,94
200	11,003	1,558	9,45	40,73
200	40,557	1,631	38,93	167,84
200	26,837	2,469	24,37	105,07
200	13,903	2,417	11,49	49,53
200	42,594	1,785	40,81	175,96
200	28,916	3,829	25,09	108,17
200	19,815	3,777	16,04	69,15
200	38,136	2,628	35,51	153,10
200	14,82	1,883	12,94	55,78
200	25,081	2,65	22,43	96,72
200	37,031	3,354	33,68	145,21
200	28,896	3,552	25,34	109,28
200	49,379	3,979	45,40	195,76
200	28,458	3,632	24,83	107,05
200	23,277	1,96	21,32	91,92
200	20,668	2,317	18,35	79,13
200	18,476	3,593	14,88	64,17
200	17,462	3,811	13,65	58,86
200	20,39	3,704	16,69	71,95
200	15,541	2,697	12,84	55,38
200	56,999	1,061	55,94	241,19
200	34,488	2,245	32,24	139,03
200	15,615	2,252	13,36	57,62
200	13,498	2,192	11,31	48,75
200	20,74	2,303	18,44	79,50
200	18,81	2,248	16,56	71,41
200	16,142	2,147	14,00	60,34
200	55,804	2,771	53,03	228,67
200	12,337	3,145	9,19	39,63
200	27,277	2,367	24,91	107,41
200	29,86	1,782	28,08	121,07
200	42,664	1,729	40,94	176,50
200	43,426	2,111	41,32	178,14
200	29,75	2,177	27,57	118,89
200	22,591	2,43	20,16	86,93
200	31,657	1,385	30,27	130,53
200	67,45	3,38	64,07	276,26
200	36,84	3,355	33,49	144,38
200	19,681	2,627	17,05	73,53
200	41,942	2,9	39,04	168,34
200	14,678	2,521	12,16	52,42
200	15,021	2,085	12,94	55,78
200	18,642	1,316	17,33	74,71
200	51,146	1,348	49,80	214,72
200	29,342	2,315	27,03	116,54
200	41,17	2,125	39,05	168,36
200	47,053	2,305	44,75	192,95
200	12,366	2,912	9,45	40,76
200	24,349	2,357	21,99	94,83
200	19,26	1,578	17,68	76,24

400	22,341	4,876	17,465	76,024
400	13,209	4,389	8,820	38,392
400	26,961	3,619	23,342	101,607
400	16,567	6,202	10,365	45,117
400	28,284	5,375	22,909	99,721
400	23,641	6,646	16,995	73,979
400	21,752	4,976	16,776	73,024
400	26,527	9,673	16,854	73,366
400	21,312	7,297	14,015	61,005
400	19,369	5,043	14,326	62,359
400	30,996	4,676	26,320	114,568
400	30,213	5,907	24,306	105,802
400	27,235	4,335	22,900	99,684
400	21,537	3,624	17,913	77,975
400	20,622	4,078	16,544	72,016
400	28,267	3,397	24,870	108,257
400	23,228	3,403	19,825	86,298
400	20,191	2,718	17,473	76,058
400	19,811	4,486	15,325	66,710
400	36,037	7,288	28,749	125,144
400	34,371	8,505	25,866	112,594
400	35,985	10,189	25,796	112,290
400	36,172	8,406	27,766	120,866
400	39,586	9,222	30,364	132,173
400	29,567	9,430	20,137	87,654
400	25,445	5,369	20,076	87,391
400	13,153	7,833	5,320	23,158
400	20,189	7,583	12,606	54,872
800	39,246	6,483	32,763	142,615
800	42,700	6,100	36,600	159,318
800	26,077	6,462	19,615	85,382
800	28,978	6,374	22,604	98,394
800	10,221	6,023	4,198	18,274
800	20,983	5,734	15,249	66,380
800	13,993	6,977	7,016	30,540
800	35,649	8,412	27,237	118,561
800	18,909	4,986	13,923	60,607
800	22,156	5,806	16,350	71,169
800	14,142	7,226	6,916	30,103
800	15,769	5,294	10,475	45,597
800	18,049	6,095	11,954	52,035
800	23,256	3,714	19,542	85,066
800	19,248	5,727	13,521	58,855
800	22,432	7,684	14,748	64,198
800	25,364	7,214	18,150	79,004
800	18,691	5,038	13,653	59,430
800	19,705	5,977	13,728	59,757
800	28,166	6,397	21,769	94,760
800	21,761	4,856	16,905	73,587
800	27,354	5,057	22,297	97,056
800	38,817	7,853	30,964	134,784
800	20,486	4,856	15,630	68,038
800	33,583	6,817	26,766	116,510
800	35,533	8,298	27,235	118,551
800	25,799	4,695	21,104	91,866
800	21,635	5,649	15,986	69,586
800	13,098	6,150	6,948	30,242
800	14,026	6,724	7,302	31,785
800	29,707	6,206	23,501	102,299
800	25,965	4,793	21,172	92,160
800	25,830	5,375	20,455	89,040
800	22,159	6,037	16,122	70,180
800	24,125	3,862	20,263	88,204
800	14,003	5,743	8,260	35,956
800	29,625	5,621	24,004	104,487
800	20,020	7,056	12,964	56,431
800	18,456	3,183	15,273	66,484
800	3,826	6,312	0,000	0,000
800	9,640	5,125	4,515	19,652
800	8,573	6,872	1,701	7,403
800	11,043	5,205	5,838	25,412

200	10,798	2,3	8,50	36,64
200	16,788	2,423	14,37	61,94
200	11,878	1,545	10,33	44,55
200	62,052	1,744	60,31	260,04
200	17,647	2,794	14,85	64,04
200	13,959	2,215	11,74	50,64
200	19,453	2,117	17,34	74,75
400	12,513	1,777	10,74	46,29
400	21,363	1,609	19,75	85,18
400	9,721	1,51	8,21	35,40
400	17,294	1,533	15,76	67,96
400	17,767	1,757	16,01	69,03
400	14,675	1,471	13,20	56,93
400	13,486	1,397	12,09	52,13
400	15,98	1,403	14,58	62,85
400	24,107	1,624	22,48	96,94
400	17,754	2,125	15,63	67,39
400	16,773	2,27	14,50	62,53
400	23,971	2,912	21,06	90,80
400	27,838	2,087	25,75	111,03
400	9,82	1,914	7,91	34,09
400	18,923	1,992	16,93	73,00
400	21,805	2,379	19,43	83,76
400	23,99	1,893	22,10	95,28
400	46,339	2,893	43,45	187,33
400	24,143	2,062	22,08	95,21
400	27,628	2,513	25,12	108,29
400	33,971	4,077	29,89	128,90
400	44,265	4,208	40,06	172,72
400	19,138	2,849	16,29	70,24
400	14,733	4,036	10,70	46,12
400	22,364	3,455	18,91	81,53
400	49,685	4,034	45,65	196,84
400	41,726	3,45	38,28	165,04
400	21,378	3,64	17,74	76,48
400	47,847	3,496	44,35	191,23
400	17,991	3,491	14,50	62,52
400	26,222	3,434	22,79	98,26
400	20,639	3,198	17,44	75,20
400	23,06	3,494	19,57	84,37
400	49,787	3,081	46,71	201,39
400	28,865	3,558	25,31	109,12
400	44,448	3,586	40,86	176,19
400	17,897	3,121	14,78	63,71
400	18,181	3,219	14,96	64,51
400	43,885	4,57	39,32	169,52
400	24,086	4,718	19,37	83,51
400	16,523	4,098	12,43	53,57
400	24,551	4,392	20,16	86,92
400	17,085	3,926	13,16	56,74
400	17,163	3,09	14,07	60,68
400	31,465	3,536	27,93	120,42
400	18,144	3,447	14,70	63,37
400	25,268	3,785	21,48	92,63
400	14,451	3,549	10,90	47,01
400	13,119	3,401	9,72	41,90
400	19,317	2,893	16,42	70,82
400	32,514	3,656	28,86	124,43
400	17,428	3,203	14,23	61,34
400	20,135	2,476	17,66	76,14
400	13,36	3,427	9,93	42,83
800	14,386	1,715	12,67	54,64
800	12,597	1,785	10,81	46,62
800	18,828	1,539	17,29	74,55
800	21,407	2,248	19,16	82,61
800	30,718	1,987	28,73	123,88
800	10,912	1,753	9,16	39,49
800	14,974	1,8	13,17	56,80
800	12,448	2,399	10,05	43,33
800	10,415	1,739	8,68	37,41
800	13,104	1,865	11,24	48,46
800	27,730	5,348	22,382	97,427
800	19,723	6,025	13,698	59,624
800	15,469	5,861	9,608	41,822
800	25,282	5,415	19,867	86,480
800	28,888	5,009	23,879	103,946
800	35,887	5,708	30,179	131,369
800	28,579	6,556	22,023	95,863
800	12,145	4,484	7,661	33,347
800	18,190	6,644	11,546	50,260
800	10,190	4,670	5,520	24,028
800	23,225	5,706	17,519	76,260
800	18,058	8,439	9,619	41,871
800	14,824	6,632	8,192	35,658
800	7,440	4,252	3,188	13,878
800	6,654	4,732	1,922	8,367

800	15,759	1,838	13,92	60,02
800	13,15	2,14	11,01	47,47
800	16,29	1,578	14,71	63,44
800	10,136	2,069	8,07	34,78
800	11,564	2,061	9,50	40,98
800	12,025	2,166	9,86	42,51
800	12,258	1,925	10,33	44,55
800	8,865	1,938	6,93	29,87
800	6,88	2,15	4,73	20,39
800	34,04	3,219	30,82	132,89
800	26,608	3,308	23,30	100,47
800	30,893	3,508	27,39	118,08
800	30,07	3,681	26,39	113,78
800	32,761	3,556	29,21	125,93
800	18,194	3,315	14,88	64,16
800	16,765	3,076	13,69	59,02
800	45,535	3,617	41,92	180,74
800	17,456	4,19	13,27	57,20
800	34,526	4,457	30,07	129,65
800	15,747	3,111	12,64	54,48
800	13,269	3,289	9,98	43,03
800	20,95	3,953	17,00	73,29
800	17,204	2,431	14,77	63,70
800	13,895	4,505	9,39	40,49
800	16,235	3,397	12,84	55,36
800	26,632	2,364	24,27	104,64
800	13,663	3,443	10,22	44,07
800	21,583	3,206	18,38	79,24
800	12,642	4,027	8,62	37,15
800	10,816	3,068	7,75	33,41
800	11,599	3,609	7,99	34,45
800	14,257	4,755	9,50	40,97
800	12,127	3,118	9,01	38,85
800	31,099	3,236	27,86	120,14
800	16,898	3,395	13,50	58,22
800	16,177	3,1	13,08	56,39
800	41,001	3,459	37,54	161,87
800	15,736	3,103	12,63	54,47
800	38,299	3,215	35,08	151,28
800	14,412	2,403	12,01	51,78
800	19,644	3,388	16,26	70,09
800	26,885	3,266	23,62	101,84
800	28,218	2,698	25,52	110,04
800	12,825	3,79	9,04	38,96
800	21,352	2,412	18,94	81,67
800	41,84	2,082	39,76	171,43
800	13,806	2,509	11,30	48,71
800	15,301	2,43	12,87	55,50
800	45,49	3,702	41,79	180,18
800	25,636	4,76	20,88	90,01
800	21,012	3,768	17,24	74,35
800	30,448	2,789	27,66	119,26
800	10,617	2,227	8,39	36,18

NacGal	IM CA	IM BKG	CA-BKG	RI%
0	36,273	1,696	34,58	267,05
0	27,665	2,298	25,37	195,92
0	15,555	1,235	14,32	110,60
0	6,865	2,71	4,16	32,09
0	31,221	1,955	29,27	226,03
0	23,379	7,664	15,72	121,37
0	5,776	1,968	3,81	29,41
0	12,604	1,588	11,02	85,08
0	8,93	1,303	7,63	58,91
0	7,261	2,198	5,06	39,10
0	5,294	3,221	2,07	16,01
0	3,89	1,504	2,39	18,43
50	7,174	1,575	5,60	43,24
50	12,403	2,061	10,34	79,87
50	10,104	1,856	8,25	63,70
50	3,274	1,613	1,66	12,83

50	10,051	2,419	7,63	58,94
50	5,564	1,341	4,22	32,62
50	3,811	1,387	2,42	18,72
50	7,377	2,066	5,31	41,02
50	12,845	1,243	11,60	89,61
50	18,292	2,383	15,91	122,87
50	14,48	1,828	12,65	97,72
50	10,597	3,021	7,58	58,51
50	15,949	1,271	14,68	113,36
50	16,631	1,354	15,28	117,99
50	11,794	2,612	9,18	70,92
50	12,47	2,746	9,72	75,10
50	7,172	1,854	5,32	41,07
50	8,85	1,965	6,89	53,18
50	10,367	2,449	7,92	61,15
50	6,626	2,628	4,00	30,88
50	9,754	1,773	7,98	61,64
50	9,179	1,752	7,43	57,36
50	13,865	1,56	12,31	95,04
50	11,509	1,608	9,90	76,47
50	17,37	2,831	14,54	112,29
50	9,686	3,512	6,17	47,68
50	10,748	3,159	7,59	58,61
50	6,629	3,287	3,34	25,81
50	8,157	3,194	4,96	38,33
50	8,699	4,865	3,83	29,61
50	7,615	4,151	3,46	26,75
100	7,244	1,109	6,14	47,38
100	5,733	2,676	3,06	23,61
100	5,617	2,834	2,78	21,49
100	3,903	2,992	0,91	7,04
100	6,061	3,453	2,61	20,14
100	41,676	3,829	37,85	292,31
100	10,558	3,224	7,33	56,64
100	20,719	4,068	16,65	128,60
100	33,042	4,862	28,18	217,64
100	9,67	3,712	5,96	46,02
100	7,115	2,62	4,50	34,72
200	11,42	2,16	9,26	71,52
200	9,515	2,437	7,08	54,67
200	20,306	1,538	18,77	144,95
200	22,731	1,413	21,32	164,65
200	7,719	2,595	5,12	39,57
200	29,45	3,43	26,02	200,96
200	12,779	3,454	9,33	72,02
200	5,605	3,407	2,20	16,98
200	7,224	3,482	3,74	28,90
200	20,019	1,922	18,10	139,77
200	4,677	3,406	1,27	9,82
200	4,551	1,343	3,21	24,78
200	10,957	1,052	9,91	76,50
400	14,039	1,993	12,05	93,04
400	6,935	1,276	5,66	43,71
400	4,208	2,099	2,11	16,29
400	5,834	2,363	3,47	26,81
400	16,188	2,212	13,98	107,94
400	6,245	2,292	3,95	30,53
400	9,257	2,312	6,95	53,64
400	9,44	6,752	2,69	20,76
400	5,793	3,45	2,34	18,10
400	5,624	3,55	2,07	16,02
400	4,144	2,206	1,94	14,97
400	4,255	3,404	0,85	6,57
400	13,245	3,705	9,54	73,68
400	13,382	1,879	11,50	88,84
400	14,415	2,551	11,86	91,63
400	17,881	2,55	15,33	118,41
400	10,596	2,398	8,20	63,32
400	7,42	2,87	4,55	35,14
400	16,973	1,911	15,06	116,33
400	15,921	2,011	13,91	107,43

400	14,843	3,015	11,83	91,35
400	11,805	2,993	8,81	68,06
400	9,644	1,891	7,75	59,88
400	5,094	1,463	3,63	28,04
800	16,155	4,164	11,99	92,61
800	18,564	2,496	16,07	124,10
800	11,428	5,078	6,35	49,04
800	17,764	4,77	12,99	100,36
800	6,626	1,242	5,38	41,58
800	7,061	4,75	2,31	17,85
800	13,257	5,629	7,63	58,91
800	14,013	4,936	9,08	70,10
800	11,556	5,227	6,33	48,88
800	17,737	4,793	12,94	99,97
800	6,761	4,385	2,38	18,35
800	11,312	5,084	6,23	48,10

Nacglu	IM CA	IM BKG	CA-BKG	RI%
0	36,273	1,696	34,58	267,05
0	27,665	2,298	25,37	195,92
0	15,555	1,235	14,32	110,60
0	6,865	2,71	4,16	32,09
0	31,221	1,955	29,27	226,03
0	23,379	7,664	15,72	121,37
0	5,776	1,968	3,81	29,41
0	12,604	1,588	11,02	85,08
0	8,93	1,303	7,63	58,91
0	7,261	2,198	5,06	39,10
0	5,294	3,221	2,07	16,01
0	3,89	1,504	2,39	18,43
50	5,007	2,635	2,37	18,32
50	16,705	2,039	14,67	113,27
50	39,78	4,463	35,32	272,77
50	50,907	5,283	45,62	352,37
50	31,028	4,176	26,85	207,39
50	16,458	4,104	12,35	95,41
50	7,284	4,02	3,26	25,21
50	6,551	3,087	3,46	26,75
50	5,339	2,346	2,99	23,12
50	7,583	1,89	5,69	43,97
50	13,988	3,102	10,89	84,08
100	5,534	1,642	3,89	30,06
100	7,73	2,023	5,71	44,08
100	4,068	1,896	2,17	16,78
100	4,392	1,744	2,65	20,45
100	13,373	2,499	10,87	83,98
100	11,819	3,233	8,59	66,31
100	9,396	2,328	7,07	54,59
100	4,584	2,461	2,12	16,40
100	24,769	3,019	21,75	167,98
100	16,454	2,449	14,01	108,17
100	6,935	1,924	5,01	38,70
100	12,942	2,51	10,43	80,57
100	8,204	3,362	4,84	37,40
100	7,793	4,102	3,69	28,51
100	8,37	3,436	4,93	38,11
100	11,355	5,393	5,96	46,05
200	6,797	3,753	3,04	23,51
200	4,722	3,115	1,61	12,41
200	7,467	3,174	4,29	33,16
200	10,159	2,5	7,66	59,15
200	6,623	3,028	3,60	27,77
200	10,725	1,563	9,16	70,76
200	5,038	3,168	1,87	14,44
200	7,073	2,157	4,92	37,97
200	7,828	2,846	4,98	38,48
200	18,119	2,853	15,27	117,90
200	16,119	3,078	13,04	100,72
200	16,952	2,592	14,36	110,91
200	4,264	2,06	2,20	17,02
200	7,628	1,675	5,95	45,98

200	4,957	2,433	2,52	19,49
200	7,578	1,705	5,87	45,36
200	9,109	5,38	3,73	28,80
200	14,392	5,742	8,65	66,81
200	9,827	1,962	7,87	60,74
200	9,713	4,123	5,59	43,17
200	13,64	2,781	10,86	83,87
200	40,117	4,405	35,71	275,82
200	28,961	1,839	27,12	209,47
200	33,627	5,354	28,27	218,36
400	15,222	3,528	11,69	90,32
400	24,576	2,626	21,95	169,53
400	10,161	3,163	7,00	54,05
400	15,323	2,31	13,01	100,50
400	10,991	2,904	8,09	62,46
400	4,888	2,028	2,86	22,09
400	12,542	3,019	9,52	73,55
400	10,198	2,02	8,18	63,16
400	36,752	2,334	34,42	265,82
400	32,803	1,971	30,83	238,13
400	19,38	2,501	16,88	130,36
400	32,542	3,83	28,71	221,75
400	10,388	2,919	7,47	57,69
400	30,934	2,085	28,85	222,81
800	4,973	1,227	3,75	28,93
800	10,654	1,019	9,64	74,41
800	7,508	1,325	6,18	47,75
800	9,29	1,95	7,34	56,69
800	7,017	2,237	4,78	36,92
800	9,139	2,319	6,82	52,67
800	12,883	3,06	9,82	75,87
800	5,481	1,714	3,77	29,09
800	7,925	2,364	5,56	42,95
800	4,477	1,339	3,14	24,24
800	11,198	1,404	9,79	75,64
800	16,108	2,272	13,84	106,86
800	7,101	2,695	4,41	34,03
800	9,501	2,784	6,72	51,88
800	6,242	2,296	3,95	30,48
800	31,7	2,336	29,36	226,79
800	22,332	1,563	20,77	160,41
800	36,659	4,519	32,14	248,23
800	9,447	4,146	5,30	40,94
800	9,718	1,965	7,75	59,88
800	3,3	0,597	2,70	20,88
800	10,184	2,226	7,96	61,46
800	3,025	2,227	0,80	6,16
800	4,453	2,057	2,40	18,51
800	12,342	1,888	10,45	80,74
800	5,353	2,332	3,02	23,33
800	5,627	1,632	4,00	30,85
800	14,629	2,471	12,16	93,90
800	7,905	1,613	6,29	48,60
800	14,933	1,847	13,09	101,07
800	21,575	2,161	19,41	149,94
800	13,163	2,791	10,37	80,11
